# A Photomechanical Film in which Liquid Crystal Design Shifts the Absorption into the Visible Light Range

**DOI:** 10.1002/advs.202302692

**Published:** 2023-09-03

**Authors:** Sven Schultzke, Nikolai Scheuring, Pim Puylaert, Matthias Lehmann, Anne Staubitz

**Affiliations:** ^1^ University of Bremen Institute for Analytical and Organic Chemistry Leobener Straße 7 D‐28359 Bremen Germany; ^2^ University of Bremen MAPEX Center for Materials and Processes Bibliothekstraße 1 D‐28359 Bremen Germany; ^3^ University of Würzburg Institute of Organic Chemistry Am Hubland D‐97074 Würzburg Germany; ^4^ University of Bremen Institute for Inorganic Chemistry and Crystallography Leobener Straße 7 D‐28359‐ Bremen Germany

**Keywords:** actuator, azo compounds, fluorinated azobenzene, liquid crystalline polymer network, liquid crystals, photomechanical effect, visible light actuation

## Abstract

Liquid crystalline polymer networks (LCN) with azobenzene monomers bend reversibly under UV‐light irradiation, combining photomechanical and photothermal effects. However, the harmful nature of UV‐light limits their use in biology and soft robotics. Although visible light‐absorbing tetra‐*ortho*‐fluoro‐substituted azobenzenes exist, liquid crystalline monomers have never been prepared. Previously, such azobenzenes were added as photoactive additives (up to 10%) to otherwise passive liquid crystalline polymer networks. In this work, a molecular design of a liquid crystalline, polymerizable azobenzene switchable by visible light is presented. The monomer assembles in a highly fluid nematic phase, but polymerizes in a layered smectic C phase. The films are produced solely from the monomer without additional liquid crystalline components and are actuated with visible light. Bending experiments in air and under water differentiate photomechanical and photothermal effects. Remarkably, a 60 µm splay aligned film maintains its deformation for hours, slowly reverting over days. Monomer liquid crystallinity is characterized using differential scanning calorimetry (DSC), wide‐angle X‐ray scattering (WAXS), and polarized optical microscopy (POM); polymer films are analyzed using WAXS and DSC on a homogeneously aligned film. The synthetic procedure is high yielding and polymer film fabrication is scalable, which enables the use of safe and efficient photomechanical LCNs in soft robotics, engineering and biology.

## Graphical abstract

A photomechanical film is prepared that can be switched with visible light. This is made possible by the design of an azobenzene nematic liquid crystalline monomer with not only tetra‐*ortho*‐fluoro substitution, but also an extended rigid core. The longer excitation wavelength leads to a high biocompatibility, but also deeper penetration depths and thus good bending behavior of the polymer film.

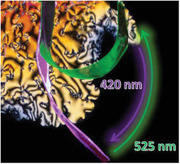

## 1 Introduction

In soft robotics, soft materials are used, which allow for new actuation principles, or different types of grippers or movement compared to those that can be achieved with hard components only.^[^ ^1^ ^]^ This greatly expands the number of tasks that can be performed with conventional rigid‐bodied robots. Soft robots can often imitate the complex natural movements better^[^ ^2^ ^]^ and may be superior in the context of our human environment,^[^ ^3^ ^]^ providing gripping,^[^ ^4^ ^]^ locomotion,^[^ ^5^ ^]^ artificial muscles,^[^ ^1^ ^]^ or oscillation.^[^ ^6^ ^]^

To achieve this ambitious goal, at present the most often used actuation design is using soft, but non‐functional materials, which are then moved pneumatically or hydraulically.^[^ ^7^ ^]^ Such solutions typically require that the robot is tethered to an air or liquid supply. A more intuitive approach is using an intrinsically functional movable material, for which bioinspired designs are becoming increasingly attractive.^[^ ^8^ ^]^ Such stimuli‐responsive materials can be actuated by changes in their environment.^[^ ^9^ ^]^ A specific subgroup of these materials comprises photoresponsive polymer films, which incorporate light‐sensitive molecules into their structure. When illuminated, these films undergo a noticeable macroscopic deformation.^[^ ^10^ ^]^ However, the reverse movement can be difficult to control. This controlled reversibility is crucial for maintaining precise control over an actuation that enables repetitive movements or movements that are controlled in their direction and amplitude as would be required for a robot. This means that the desideratum for a photoresponsive film is that actuation in both directions is controlled by defined wavelengths of illumination and little uncontrolled thermal actuation.

For such a reversible photo response, the most investigated molecular switch is azobenzene and its derivatives.^[^ ^11^ ^]^ Upon irradiation with light, azobenzene undergoes a *E*‐ to *Z*‐isomerization resulting in a geometrical change: The distance between the carbon atoms at the *para*‐position is 9 Å in the planar, thermally stable *E*‐isomer and is reduced to 5.5 Å in its non‐planar, metastable *Z*‐isomer.^[^ ^12^ ^]^ By incorporating azobenzenes as mesogens of liquid crystals and attaching polymerizable, flexible linkers, it is possible to prepare light‐switchable liquid crystalline elastomers or the more densely cross‐linked liquid crystalline polymer networks (LCNs) from these monomers.^[^ ^13^ ^]^

In photoswitchable LCNs, combination of light‐induced processes leads to a macroscopically observable bending effect, although the precise underlying mechanisms are still a subject of debate:

All mechanisms are based on the fact that the polymer films are highly anisotropic in their morphology, irrespective of which alignment of the mesogens is present. In addition, they all take into account that the light illuminates the film from one side and that the extent of the photoreaction of the azobenzenes depends on a) their alignment with respect to the incident light; b) the penetration depth of the light, i.e., into what depth of the film does isomerization take place. However, the matter is complicated by the fact that the illumination of azobenzenes may lead to a substantial heating of the polymer film, because first, not all azobenzene molecules isomerize, but some will merely follow a vibrational relaxation and second, even the azobenzenes that do isomerize can thermally relax to the *E*‐isomer. Furthermore, as there are no azobenzene monomers LCs, in which the spectra of the *E* and *Z* isomers are fully separated, in all cases under constant illumination, a photostationary state (PSS) will be reached that continues to produce heat. Heating polymers leads to changes in volume and modulus (among other parameters) and thus separating different effects is not always possible.

The first and most simple mechanism considers the substantial geometry change of the azobenzenes upon photo‐isomerization.^[^ ^14^ ^]^ If the azobenzenes are parallel to the plane of the film (homogeneous alignment), then the isomerization of the first few layers of azobenzene will give rise to a shortening and thickening of these layers, causing overall contraction and bending toward the light source. Conversely, in azobenzenes arranged perpendicularly to the plane of the film (homeotropic), the isomerization will cause a sidewise expansion, leading to a bending away from the light. In this way, a molecular movement is translated to a macroscopically observable movement.

However, later experiments suggested that the mechanism might be more complex:^[^ ^15^ ^]^ the basic hypothesis that the geometrical change leads to a change in volume (or density) in the illuminated layers still holds. However, not only is the *E* to *Z* isomerization thought to increase the volume, but also the reverse, *Z* to *E* isomerization. This takes into account that not only do the isomers themselves have different spatial requirements (a thermodynamic point), but also the molecular movement itself requires space (a kinetic point). This work argues that the thermal expansion (a photothermal effect) as a result of heating by the thermal relaxation of the *Z*‐isomer would be insufficient to explain their results. This is also borne out by earlier work,^[^ ^16^ ^]^ in which the thermal expansion for thin (15 µm) azobenzene doped films (2–20%) was measured by conventional heating rather than photothermal heating and comparing the obtained values. Although the polymer did heat up to ca. 60 °C, (with a very high illumination power (1 W cm‐^2^)), thermal expansion alone could not explain the results.

An ingenious setup to separate the two effects (photothermal and photomechanical) was proposed by Finkelmann and co‐workers.^[^ ^17^ ^]^ They used a set‐up to measure the mechanical stress as a function of irradiation or non‐irradiation (i. e. darkness) for samples in which the azobenzene was part of the cross‐linker and for others, in which it was pendant. By using an IR filter to remove any irradiative thermal heating they could quantify and separate a quite substantial thermal contribution to the stress in the film. Moreover, they concluded that the most relevant parameter for strong actuation is cross‐linking the azobenzene into the LCN rather than using it as a side chain, which led to less actuation.

Another technique that is often used to separate the photothermal from the photomechanical effect is to perform the bending experiments under water, because water can serve as a heat sink. However, the interactions between water and LCNs are complex and because not all reports include these measurements under consideration of all potential experimental parameters, our understanding of the detailed effects of water or other media is still only rudimentary.^[^ ^18^ ^]^ In this context, the emergence of photo‐switchable liquid crystalline hydrogels is worth mentioning, which are materials specifically adapted to underwater actuation with soft materials.^[^ ^19^ ^]^

Irrespective of whether a photomechanical and / or photothermal effect is the most important for a light‐bendable polymer film, an intriguing question remained of how this macroscopic movement of azobenzene LCNs could occur below the glass transition temperature. This was addressed by an IR spectroscopic study that was able to prove that on the molecular level that the effective temperature could reach well beyond 200 °C, while the material in bulk stayed much cooler.^[^ ^20^ ^]^ This heating was highly heterogeneous and led to the postulate that essentially, this local photothermal effect may be disguised as an order‐disorder effect or geometrical effect in experiments that observe purely macroscopical parameters. Photosoftening that is not merely a simple thermal effect was also experimentally shown by nanoindentation experiments.^[^ ^21^ ^]^ Importantly, whatever the mechanisms in the particular examples are, the materials bend upon irradiation with light.^[^ ^22^ ^]^

In addition to these different mechanisms, there is substantial variety in film fabrication, leading to different geometries, in particular film thicknesses and different morphologies. In general, twisted nematic (TN) or splay arrangements have been shown to generate the highest bending effect.^[^ ^23^ ^]^ Film thicknesses vary greatly between 2^[^ ^24^ ^]^ and 48 µm,^[^ ^25^ ^]^ but thicknesses ≈20 to 30 µm are most common.^[^ ^26^ ^]^ This is a limitation, because although thinner films bend more easily, they are more fragile and less useful in actuators.

For photomechanical polymers, certain basic robotic movements have been described.^[^ ^26^, ^27^ ^]^ However, the photo sensitive molecules used in this first generation of smart material require harmful UV‐light or blue light (blue light can also be harmful for organisms. See for example ref. [28, 29]) for their activation (360 – 440 nm).^[^ ^30^ ^]^ Avoiding the necessity of UV‐light for actuation is a required step if one wishes to use this material for a broader range of applications especially at the interface of robots and living beings. In addition, two technical aspects favor visible light as a desirable stimulus: First, the depth of penetration for longer wavelengths is significantly larger: This parameter is influenced by the absorption coefficient and the scattering of the light. In general, for shorter wavelengths, more scattering occurs compared to Vis or NIR light, resulting in less penetration depth.^[^ ^31^ ^]^ Second, linearly polarized light (LPL) can be generated by using linear polarizer, which is more accessible in the visible range^[^ ^32^, ^33^ ^]^ and enables photoalignment (Weigert effect).^[^ ^34^ ^]^ In combination with the deeper penetration depth of visible light, higher photomechanical and photoalignment effects might be observed. Despite these compelling advantages and recent reports^[^ ^32c–f^ ^]^ of photoactive molecules that do switch with visible light, the number of photomechanical materials based on such compounds is low: One attempt was to use tetra‐*ortho*‐fluorinated azobenzene derivatives, which are known for their absorbance in the visible range.^[^ ^32^, ^35^ ^]^ However, upon incorporation of these molecules into a polymer, the photomechanical effect vanished:^[^ ^36^ ^]^ The problem was the loss of liquid‐crystallinity in the solid. A way to circumvent this problem has been to use tetra‐*ortho‐*fluorinated azobenzenes merely as co‐monomers with loadings of up to 10%.^[^ ^36^, ^37^ ^]^ In this way, the liquid crystallinity of the LCN is imparted by a conventional LCN mixture, whereas the photoactivity in the visible range can be attributed to photoswitching of the azobenzene. Other successful attempts to move the absorption of switchable LCNs into the visible range have been described. A thin film of a thickness of 12 µm with incorporated azotolane moieties was also switchable with visible light. While the bending motion (436 nm) was achieved within a few seconds, the unbending motion with light >577 nm was only after a few minutes without being able to fix the geometry for a longer time.^[^ ^38^ ^]^ Further work in this area led to bending with even longer wavelengths using upconversion.^[^ ^26^, ^39^ ^]^ However, this approach necessitated a bilayer configuration, in which the controlled unbending had to be sacrificed.

In this work, we present a significant advancement by introducing a novel approach to the design of visible light switchable monomers for photomechanical liquid crystalline networks (LCNs). Our innovation lies in the reintroduction of liquid crystallinity to a tetra‐*ortho*‐fluorinated azobenzene monomer. Unlike previous methods, our newly designed molecule possesses both liquid crystalline properties and the ability to undergo switching with visible light. Notably, this monomer can be polymerized within a liquid crystal cell (LC) without the need for passive liquid crystalline phase forming co‐monomers, which typically limit azobenzene concentrations below 10%. The resulting films exhibit a remarkable reversible bending movement on a macroscopic scale. Because of the actuation wavelength of 425 nm, absorption and scattering of this light in water is low and thus, underwater actuation is also possible.

## 2 Liquid Crystal Design of *Ortho*‐Fluorinated Azobenzene

In order to create an intrinsically liquid crystalline monomer capable of absorbing within the visible range of the spectrum, several design principles were taken into account for this study.

First, it was crucial to ensure a high selectivity for each photostationary state by maximizing the separation of the absorption bands (π→ π* and/or n→ π*‐absorption) of the two isomers.

Second, the metastable isomer's half‐life time needed to be as long as possible to ensure reliable actuation without the occurrence of uncontrollable thermal relaxation reactions. Ideally, all actuation should be photomechanical rather than photothermal. Before polymerization, the monomers were to be aligned in a hybrid aligned nematic (HAN) cell to enable a subsequent anisotropic bending.^[^ ^40^ ^]^

Therefore, the design of a photomechanical LCN requires a molecule, which is reversibly switchable with visible light; which has a long thermal half‐life time; which is a liquid crystal, and which also contains polymerizable units. This has been attempted previously using monomers based on the tetra‐*ortho*‐fluorinated azobenzene, no formation of LC phases with the pure monomer were reported^[^ ^37a,c^ ^]^

Previous investigations on fluorinated terphenyl systems with similar structural characteristics to our target molecule have demonstrated the presence of nematic phases.^[^ ^41^ ^]^ The elongation of the azobenzene core with a benzoic acid ester motif has also been used before in an LC monomer designed for polymerization.^[^ ^42^ ^]^ Because of the lack of fluorine atoms, this LC does not switch with visible light, but with 365 nm. This monomer mixture shows a smectic C' (SmC') and SmC phase, which can be polymerized into a SmC' or rectangular columnal phases.

**Figure 1:**
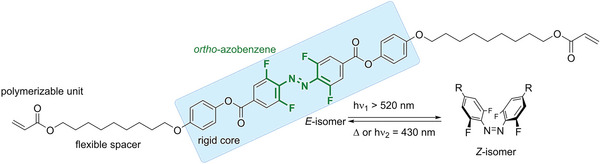
Design of an azobenzene switchable with visible light and being a liquid crystal.

These reports encouraged us to combine tetra‐*ortho*‐fluorinated azobenzenes with a benzoic acid ester motif, reintroducing liquid crystallinity by expanding the rigid core along with a polymerizable acrylate linked by a long aliphatic spacer (**Figure** **1**).

### 2.1 Synthetic Strategy

**Scheme 1:**
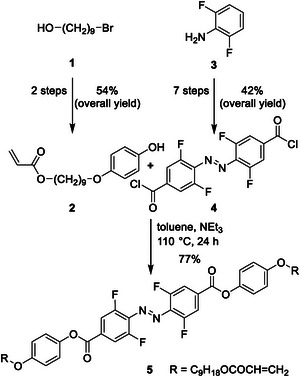
Key steps of the synthesis of (*E*)−4,4′‐(diazene‐1,2‐diyl)bis(3,5‐difluorobenzoic acid) (**5**) via the di‐acyl chloride as reactive intermediate. Compound **4** was synthesized in 7 steps starting with 2,6‐difluoroaniline (**3**). The second building block **2** was prepared over two steps from 9‐bromononanol (**1**). Details see Supporting Information.

The synthesis process (**Scheme** **1**) followed a highly efficient convergent strategy utilizing two large fragments (**2** and **4**). Building block **2** was easily obtained with an overall yield of 54% through standard transformations (for detailed information, see Supporting Information). Initially, the coupling fragment containing the azobenzene was envisioned as a di‐carboxylic acid. However, a conventional Steglich esterification yielded the target molecule **5** with only a 20% yield. Consequently, we synthesized the more reactive di‐acyl chloride azobenzene derivative. The crucial convergent step involved joining both fragments through an esterification reaction, resulting in the formation of monomer **5** with a yield of 77%.

### 2.2 Switching Properties

**Figure 2:**
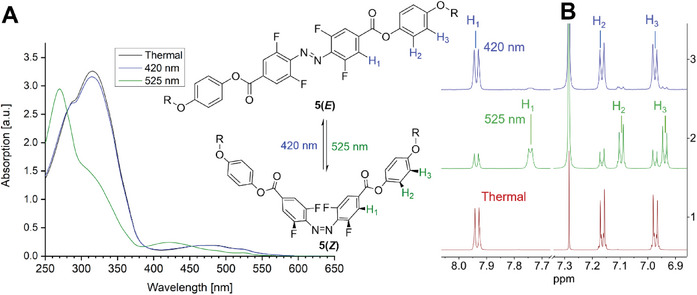
A) Switching properties of **5** determined by UV–vis spectroscopy in CHCl_3_ (conc.  =  0.16 µL mol^−1^)). B) The quantification of the switching determined via ^1^H NMR spectroscopy in CDCl_3_ (conc. = 0.28 µL mol^−1^).

The switching properties were analyzed using UV‐analysis in combination with NMR spectroscopy to quantify the *E* / *Z* ratios of the photostationary states (PSS). Compound **5** was irradiated with different wavelengths (420 nm (33 mW cm^−2^), 490 nm (28 mW cm^−2^), 525 nm (20 mW cm^−2^), 565 nm (21 mW cm^−2^) for one minute for the UV–vis spectroscopic study and five minutes for the NMR spectroscopic study (**Figure** **2**, further information in the Supporting Information).

The required irradiation times for achieving the respective (PSS) were dependent on the sample concentration and the intensity of irradiation. The UV spectra exhibited well‐separated n→π* absorption bands (Δ = 49 nm) due to the combined influence of fluorine atoms and the electron‐withdrawing effect of the *para*‐positioned phenyl benzoate motif.^[^ ^32c^ ^]^ The π→π* transition of the *Z*‐isomer was observed at a wavelength of 316 nm in cyclohexane (315 nm in chloroform), while the n‐π* transition reached a maximum of 471 nm in cyclohexane (482 nm in chloroform). Regarding the *Z*‐isomer, the n→π* transition took place at 422 nm in cyclohexane (421 nm in chloroform), but the π→π* transition overlapped with that of the *E*‐isomer. The *E*‐isomer's n→π* transition could be induced using green light (< 525 nm) to isomerize the tetra‐*ortho*‐fluorinated azobenzene monomer **5** into its *Z*‐isomer (70% yield, maximum n→π*‐absorption at 421 nm). Subsequently, violet light (420 nm) facilitated the transformation of the molecules back to the *E*‐isomer (93% yield, maximum n→π* absorption at 470 nm).

The *E*/*Z* ratios of the photostationary states in CDCl_3_ and benzene‐*d_6_* were determined through complementary NMR spectroscopic analysis (see **Table** **1**, Figure 2). Upon irradiation with green light, the proton signals of the aromatic ring (designated as H_1_, H_2_, and H_3_) exhibited highfield shifts, indicating the formation of the metastable *Z*‐isomer of compound **5**. Green light irradiation at a wavelength of 525 nm resulted in the highest enrichment of the *Z*‐isomer (70%), whereas violet light at 420 nm led to the highest enrichment of the *E*‐isomer (93%). The *E* / *Z* ratio and the UV–vis spectra were largely independent of the solvent.

**Table 1:** Amount of *E* and *Z* isomers in the PSS determined by NMR spectroscopy after irradiation of *E*‐**5** with different wavelengths in the solvents chloroform‐*d* (conc. = 0.28 µL mol^−1^) and benzene‐*d_6_* (conc. = 0.26 µL mol^−1^).

	Chloroform‐*d*		Benzene‐*d_6_*	
	*E*‐**5**	*Z*‐**5**	*E*‐**5**	*Z*‐**5**
Thermal	100%	0%	100%	0%
420 nm	93%	7%	92%	8%
490 nm	53%	47%	57%	43%
525 nm	30%	70%	30%	70%
565 nm	39%	61%	35%	65%

The thermal half‐life time (t_1/2_) of Z‐**5** was determined by UV–vis spectroscopy to be 49 d (25 °C, toluene). This value is in accordance with a similar azobenzene in the literature: (diethyl 4,4′‐(diazene‐1,2‐diyl)‐bis(3,5‐difluorobenzoate): t_1/2_ (60 °C, MeCN): 14 h, **5**: t_1/2_ (60 °C, toluene) = 12 h. (Figure S3, Supporting Information).^[^ ^35^ ^]^

### 2.3 Liquid Crystal Analysis

**Figure 3:**
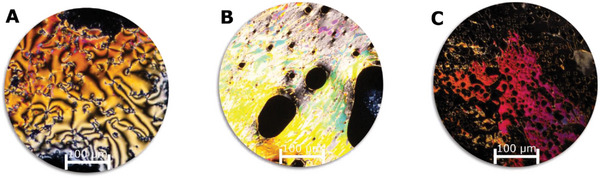
A) POM image of **5** at 130 °C between two glass slides showing nematic Schlieren textures. B) Strong birefringence of **5** after mechanical shearing of the homeotropic aligned phase (completely black domain; not shown) C) After the mechanical shearing the homeotropic alignment restores within a few seconds. The image shows the fading out of the strong birefringence. All images were taken through crossed polarizer.

The thermotropic properties of **5** were studied by means of polarization optical microscopy (POM), differential scanning calorimetry (DSC) and a wide‐angle X‐ray scattering (WAXS). The DSC heating scan of **5**, in the range of −20 °C to 170 °C at a scan rate of 5 K min^−1^, showed a large endothermic phase transition at 119.8 °C with a large transition enthalpy of 99.1 kJ mol ^‐1^, which can be attributed to the melting of the material (**Figure** **3**A). At 152.3 °C a second phase transition occurred with a transition enthalpy of only 0.1 kJ mol^−1^. This indicates the existence of a liquid‐crystalline phase of low positional order. The cooling scan revealed a similar phase transition at 152.5 °C, i.e., no hysteresis was observed. The liquid crystal can be subsequently supercooled and recrystallized at 100.9 °C. Thus, a typical hysteresis of ≈20 °C is determined for the melting.

The POM analysis yielded further insight into the nature of the liquid‐crystalline phase. Compound **5** reveals a highly fluid phase with a characteristic Schlieren texture. This points to a nematic order in the LC phase (Figure 3A).

**Figure 4:**
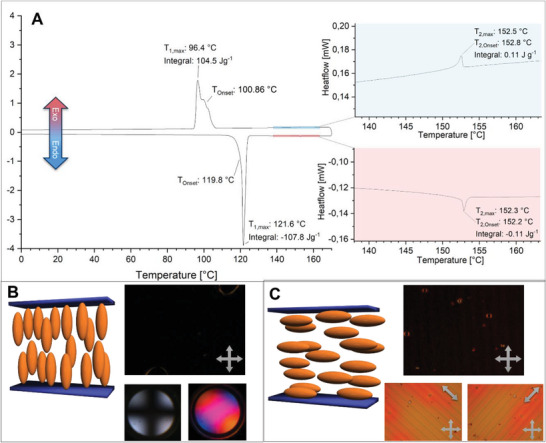
A) The DSC shows the first cycle (5 K min^−1^, 6.0481 mg) in the temperature range −40 °C to 170 °C after annealing the sample at 120 °C for one hour in an isothermal program to ensure full *E*‐isomerization. B) The image is taken from the POM at 130 °C after annealing the sample without light irradiation. The molecule showed a spontaneous homeotropic alignment between two glass slides. Consocopy confirmed the uniaxial, optically positive nature of the phase. C) Between an ITO coated glass slide with planar alignment layers, a planar alignment could be enforced. The sample was turned by 45° relative to the polarizers to show the highest birefringence and thus the alignment of the nematic director.

To achieve a uniform self‐assembly of the mesogens, the sample was allowed to equilibrate at 130 °C in the dark for 60 min. This treatment resulted in a homeotropic alignment. Subsequent conoscopy confirmed the uniaxial, optically positive nature of the nematic liquid crystal (**Figure** **4**B). Mechanical shearing of the homeotropic texture caused strong birefringence originating from the induced planar alignment of the molecules (Figure 3B). However, the homeotropic alignment was rapidly restored (Figure 3C). In an indium tin oxide (ITO) coated cell with planar alignment layers, a planar alignment of **5** could be enforced. Rotating the sample out of the plane (45°) of the crossed analyzer/polarizer position, resulted in the highest birefringence (Figure 4C).

**Figure 5:**
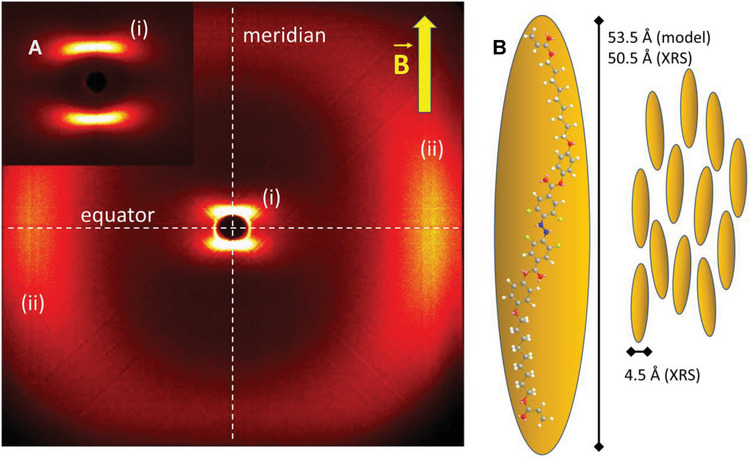
A) WAXS pattern recorded in the liquid crystal phase of compound **5** at 148 °C. The liquid crystal was aligned in the magnetic field (yellow arrow). The small angle signals i) are aligned with the meridian and the wide‐angle signals ii) with the equator. Inset: Signals at small angles recorded at a larger sample‐detector distance in order to avoid the influence of the beam stop. This demonstrates the quality of the alignment. B) The molecular long axis is aligned parallel with the magnetic field. The XRS length is in good agreement with the length of the fully extended mesogen.

WAXS studies were performed in the liquid crystal phase at 148 °C using ring magnets (1 Tesla) to induce uniform alignment (**Figure** **5**). Only two broad signals could be observed at small and wide angles respectively, which correspond to distances of 50.3 and 4.5 Å. The pattern showed that the molecular long axes are aligned with the magnetic field, which is reasonable since the diamagnetic ring current of the aromatic rings are turned out of the field, minimizing the energy of the system. The correlation lengths were calculated by the Scherer formula^[^ ^43^ ^]^ based on the full‐width‐at‐half‐maximum of the X‐ray signals and amount to 2.8 molecular lenghts for the small angle and 6.5 for the wide angle signal. These values are indicative of a short range positional order like in a nematic phase. Consequently, along the director, the material has a liquid like correlation, which increases slightly in the transverse assembly. The XRS distance can be attributed to the molecular length, which is 53.5 Å for a model of a fully extended calamitic mesogen **5** and 4.5 Å for the average molecular breadth. Thus, the data are in full agreement with an optically positive nematic phase.

The wide‐angle X‐ray scattering (WAXS) data may also indicate the possibility of a SmA (smectic A) phase. The SmA phase is the second lowest ordered liquid crystal (LC) phase following the nematic phase. Optically, the SmA phase exhibits positive birefringence for rod‐shaped mesogens, resulting in conoscopic images similar to those observed in a homeotropically aligned nematic phase. It is expected to align in a magnetic field in a manner identical to the nematic phase. The transition enthalpy associated with the SmA phase is also very small. However, it is worth noting that Schlieren texture, a characteristic feature, is not reported for SmA phases; instead, it is observed in SmC phases or nematic phases. Considering the WAXS data and the uniaxial nature of the phase, which is inconsistent with a SmC phase, the most plausible option is a nematic phase. Furthermore, the relatively large full‐width‐at‐half‐maximum indicates a very small correlation length, resembling that of a liquid. In particular, for this molecule, the correlation length associated with its long axis is short.

**Figure 6:**
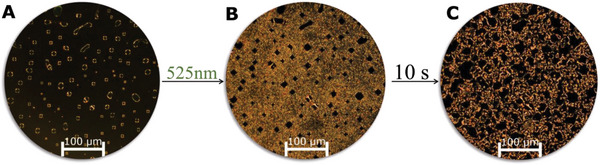
A) POM Image of **5** at 130 °C at the homeotropically state between two glass slides. B) The sample was irradiated with green light (525 nm) for 20 s, during which time yellow‐orange sparkles appeared. C) The transition of **5** without irradiation by thermal relaxation toward its original state. All images were taking through crossed polarizer.

When the nematic phase at 130 °C was irradiated with light of 525 nm under the POM, to switch the molecule into the *Z*‐isomer, the birefringence was lost; the Z‐enriched sample was isotropic at this temperature. After turning off the light source, colorful sparkles appeared, which we assign to events of thermal relaxation of the *Z*‐isomer to the *E*‐isomer leading to the immediate reorientation of **5** (**Figure** **6**).

### 2.4 Single Crystal X‐Ray Diffraction

In addition to the liquid crystalline properties of **5**, it was desirable to understand the structure in the crystal on a molecular level. Unfortunately, it was impossible to grow single crystals of **5**; therefore, a structurally similar molecule, **6**, was prepared. In this molecule, the flexible chain length was shortened which facilitated the growth of single crystals and no acrylate groups were present (For the synthetic details see the Supporting Information). This derivative **6** proved to be liquid‐crystalline as well with similar properties to **5** (melting transition at 99 °C, nematic to isotropic transition at 240 °C, for details see the Supporting Information). Given that the rigid part of compound **6** is identical to that of compound **5**, the crystal structure of compound **6** serves as a suitable model for assessing the intermolecular interactions of monomer **5**.

The geometry for the parent azobenzene is planar with a N=N bond length of 1.242 Å, a N=N bond geometry of 180 ° (C‐N=N‐C Ψ dihedral angle), a C‐N=N angle of 114.1 (2x) and a C‐C‐N=N dihedral angle Φ of 0°.^[^ ^12^ ^]^ The tetra‐*ortho* fluorination of azobenzene changes the geometry of the C‐N=N angle (124.6° and 125.3°) and the dihedral angle Φ (40.5°), but only slightly the Ψ dihedral angle (179.2°). This results in the phenyl‐rings remaining parallel to each other.^[^ ^44^ ^]^ Larger substituents in the *ortho*‐position are reported to change both dihedral angles.^[^ ^45^ ^]^

Surprisingly, the tetra‐*ortho*‐fluorinated azobenzene **6** shows changes in both Φ and Ψ dihedral angles: The Ψ dihedral angle was 170.2° and the Φ dihedral angle was uncommonly high with 59.8°. This is very unusual for a tetra‐*ortho*‐fluorinated azobenzene and results in highly twisted phenyl‐rings (60.5 °, measured by the angle of planes through the phenyl‐rings.) around the azo‐bridge. The N=N bond is elongated to 1.255 Å and a C‐N=N angle of 112.2° and 112.7° has been observed.

**Figure 7:**
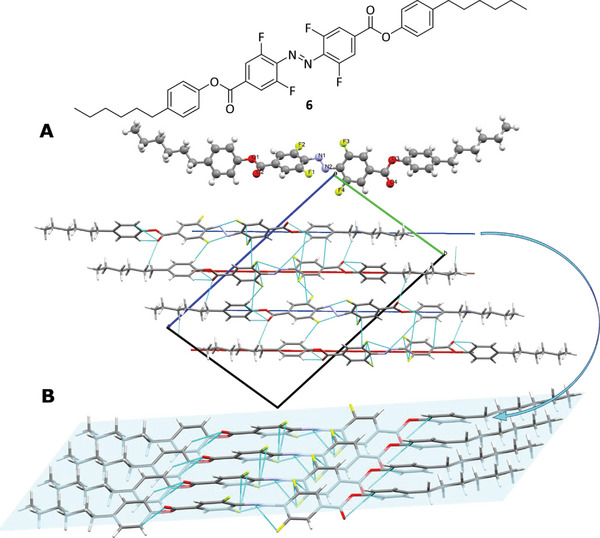
Molecular structure of derivative **6** with its in‐plane A) and out‐of‐plane B) inter‐molecular interaction.

Within the crystal structure, the aligned molecules are packed densely within one layer (colored in blue, **Figure** **7**). The interaction between the layers is different to the interaction within the layer. Between the layers, an azo‐group stacks on top of an ester‐group leading to a stair‐wise stacking (Figure 4). While it is difficult to transfer specific features within the crystal packaging to the mesogen order and alignment in the liquid crystal phase, these observations in the crystal are similar to the tilt of mesogens in SmC phases, which were reported also for the fluorine substituted terphenyl as the low temperature phase below their nematic temperature interval.^[^ ^41^ ^]^ The tilt angle in the crystal of **6** was measured at ca. 35° (see Supporting Information for details, and see below for the morphology of the polymer film measured by WAXS). Moreover, the crystal structure confirms that a complete parallel packing with the cores at the same lateral position is sterically impossible, but it allows the formation of a high temperature nematic phase with a wide temperature interval of such compounds. Therefore, it becomes clear that the preferred alignment of such mesogens is parallel to the molecular long axes, as expected for such calamitic mesogens.

## 3 Liquid‐Crystalline Polymer

### 3.1 Preparation of the Thin Film

There are several types of LC cells that can be used for the alignment of LC monomers: Both top and bottom can be homeotopically or homogeneously aligned. Alternatively, a hybrid aligned nematic (HAN) type LC‐cell can be used, which leads to a splay alignment or a twisted nematic (TN) morphology can be used. For optimal bending results, a splay alignment or twisted nematic alignment is best, because it has been shown that the mechanical work in such morphologies is much increased compared to purely homogeneously aligned films.^[^ ^46^ ^]^ In addition, splay or TN alignments have the advantage that the film can bend both toward the light, but also away from it:^[^ ^47^ ^]^ The planar alignment (Side **Pl**) of the mesogens leads to a contraction of the mesogen upon irradiation resulting in a bending toward the light source.^[^ ^46^, ^47^ ^]^ On the other side of the polymer, the mesogens are aligned hometropically (Side **Ho**) resulting in an expansion upon isomerization and a bending away from the light source.^[^ ^46^, ^47^ ^]^ The overall splay‐bend alignment within the cell also increases the anisotropic bending effect.^[^ ^40^, ^46^ ^]^

In addition, a splay alignment leads to better control of the bending motion than other twisted nematic and uniform director distribution arrangements,^[^ ^23^ ^]^ because of the suppression of saddle inducing deformations and giving higher bending angles.

However, for the optical and structural analysis of the film, the additional dimension of depth anisotropy introduced into the film by a splay alignment adds much complexity and a homogeneous arrangement was produced as a simplified model of the film.

For the hybrid aligned nematic (HAN) type LC‐cells, two polyimide spin‐coated glass substrates were used; one of them was rubbed over a velvet cloth for planar alignment, whereas the second one was left untreated. For the homogeneous alignment, both were rubbed over the velvet cloth. The two glass substrates were spaced by thin films of PTFE stripes of different thicknesses. Subsequently, the glass substrates were glued together with UV‐acrylate adhesive Norland 65. A mixture prepared of **5** and the photoinitator (phenyl‐bis‐(2,4,6‐trimethylbenzoyl)‐phosphine oxide (BAPO), 3 mol%) were dissolved in DCM and the solvent subsequently removed in vacuo. The cell was placed on a heating stage; the dried mixture was melted (isotropic phase at 155 °C) and allowed to be sucked into the LC cell by capillary forces in the dark. Then, the cell was slowly cooled (0.5°C min^‐1^) to 130 °C to reach the nematic phase. Subsequently, polymerization was initiated by using linearly polarized 420 nm light from an LED source, with the polarizer orthogonal to the planar alignment of the LC cell. In addition, a PMMA slide was used as a filter so that the photocatalyst could be activated, while avoiding photoisomerization.^[I]^ After 24 h the cell was opened and the film was extracted by sliding a razor blade underneath, which had been rinsed with hot water to reduce friction between the blade and the polymer during extraction (for further details see the Supporting Information).

The films were dried and their thickness was measured using an electronic cantilever. The splay aligned films had thicknesses of 40 µm (denoted **P_planar,40_**), 60 µm (denoted **P_planar,60_**), and 105 µm (denoted **P_planar,105_**) and for the homogenous alignment, films with the thicknesses of 31 µm (**P_planar,31_)**, 49 µm (**P_planar,49_),** and 80 µm (**P_planar,80_**) were produced.

### 3.2 Structural and Optical Characterizations of the Polymer Films

**Figure 8:**
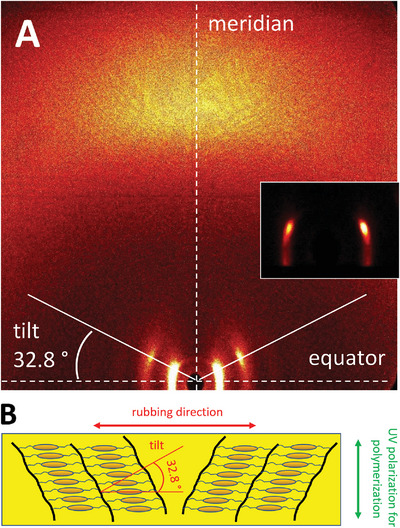
A) WAXS diffraction pattern of a 49 µm thick film (**P_planar,49_**)^i)^ at 25 °C. Inset: The intensities of the small angle reflections at the left and right are different since they originate from different domains of synclinic SmC phases. B) Schematic for the film orientation for the WAXS experiment, rubbing and alignment direction as well as the direction of the polarized UV light used for the polymerization. The red lines show the tilt of the mesogens versus the layer normal. ^i)^ The other planar aligned films showed the same pattern.

The planar homogeneously aligned films served as models that would allow the morphological assessment of the new LCN films, now entirely composed of the azobenzene. The films (**P_planar,31_)**, (**P_planar,49_),** and (**P_planar,80_)** were analyzed using wide‐angle X‐ray scattering (WAXS) measurements (**Figure** **8**). The obtained patterns stem from distinct synclinic SmC phases that manifest in varying domains within the films to different extents. Consequently, it is not uncommon for the intensities of the left and right signals to exhibit disparities. Within the SmC phases, the aromatic rings exhibit enhanced packing efficiency, giving rise to a subtle π‐π signal. Remarkably, the WAXS signal aligns precisely along the bisecting line, orthogonal to the rubbing direction, offering a plausible explanation for its well‐defined position.

Whereas the LC phase of the monomer was nematic, the polymerization process significantly augmented the level of order within the film; it is now smectic C. It is reasonable to presume that the molecular structure closely resembles that of the crystalline state of the model compound **6**; in this crystal structure, a tilt angle of ca. 35° was found which is very close to the tilt angle of ca. 32° found in the polymer prepared from **5**. The layer thickness for this LC morphology was now 46 Å and the correlation length 366 Å corresponding to 7.9 molecular units compared to 2.8 units in the monomer. Measurements at higher temperatures (93 °C) did not significantly change the WAXS pattern; the correlation length dropped only slightly to 7.7 molecular units (see Supporting Information for the diffraction pattern). Therefore, it can be concluded that the smectic C phase is preserved even at higher temperatures.

To understand the thermal properties of the polymer, a differential scanning calorimetry (DSC) measurement was performed on the **P_splay,105_** film, because it was the thickest and thus gave the best signal to noise ratio. The measurement used a heating rate of 10 K/min for the temperature range −40 °C to 170 °C. We observed a glass transition temperature with an onset temperature of 60 °C, with some amount of enthalpy relaxation (see Supporting Information^[^ ^48^ ^]^).

### 3.3 Photochemical Bending

For the photochemical bending experiments, a polymer film, **P_splay,60_**, was employed. The film was precisely cut into a piece measuring 1.5 cm x 0.2 cm x 60 µm. The piece was then vertically fixed, ensuring the side with homogeneous alignment faced the light source. The use of relatively large films was preferred for two reasons: first, the ability to easily bend thick films is uncommon (as discussed in the introduction), and second, for potential applications in soft robotics, a minimum size requirement is necessary. To facilitate underwater actuation, the same experimental setup was utilized, with the polymer immersed in a quartz cuvette filled with water (measuring 5 cm x 5 cm x 10 cm). Two light sources were utilized for the experiments: an LED emitting green light at 525 nm and another emitting blue light at 420 nm. These LEDs were focused on the polymer strip to induce bending. The intensity of light reaching the polymer could be adjusted between 40 and 200 mW cm^−2^, depending on the collimator's focus. This range was a compromise between exposure strength and area since the LED beams were not wide enough to fully irradiate the sample with both optimal focus and intensity.

### 3.4 Photochemical Bending in Air

**Figure 9:**
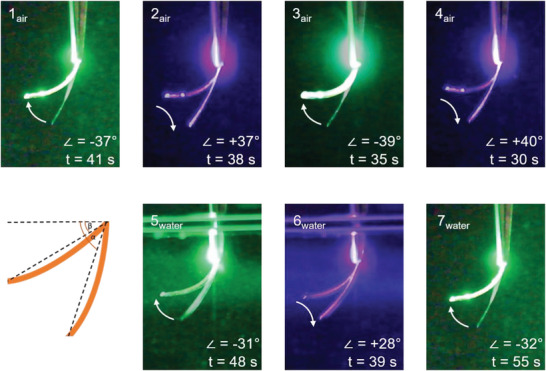
Analysis of the bending angles of the **P_splay,60_** film in air (top row) and in water (bottom row) depending on the illumination wavelength (525 or 420 nm). The images are overlayed stills from the video Video S3 (Supporting Information). The angle given in the images are the amplitude change between both angles (∠ = starting angle – ending angle; see schematic for its definition) and the sign indicated the change upon illumination; the time indicates the duration of illumination required to reach this stage.

Exposure of the **P_splay,60_** film to green light (525 nm) caused bending in air, resulting in a final bending angle amplitude of 40° at a rate of 1°s (see **Figure** **9**). The ultimate bending angle was primarily influenced by the geometry and intensity of irradiation. By adjusting the lamp's position during the film bending process, even a U‐shaped configuration could be achieved (Figure S18, Supporting Information). Importantly, the bending behavior, driven by the *E*‐*Z* isomerization of the tetra‐*ortho*‐fluorinated azobenzene monomer, could be completely reversed by illuminating the film with blue light (420 nm). However, if the bent film was left in the dark at 23 °C, its shape only relaxed by 25° within 3 days. This characteristic is particularly advantageous for applications requiring precise control over movement solely through light manipulation, as it circumvents the largely uncontrollable thermal relaxation that occurs as a background reaction. In contrast, most azobenzene‐based liquid crystal networks (LCN) and liquid crystal elastomers (LCE) fully relax within 24 h. The polymer film **P_splay,105_** showed only a slow bending motion with a small amplitude, which is presumably due to the relatively low illumination power in combination with the film thickness.^[^ ^49^ ^]^ The temperature change caused by the illumination was measured using an IR camera (see Supporting Information). Illumination with 525 nm led to a temperature increase to 40 °C, and 420 nm caused heating to 33 °C. Some heat development during illumination is to be expected due to thermal relaxation (see introduction), and molecular local heat may be higher,^[^ ^20^ ^]^ but at least macroscopically, this points to a relatively small photothermal effect.

### 3.5 Bending under Water

Water exerts a profound influence on the behavior of light passing through. In particular, higher energy radiation experiences scattering^[^ ^50^ ^]^ and backscattering^[^ ^51^ ^]^ phenomena, which diminish as the wavelength increases. However, at ≈600 nm and beyond, absorption by water significantly intensifies.^[^ ^52^ ^]^ These contrasting tendencies establish a distinct optical window for water, spanning roughly 400 to 500 nm. Consequently, photoswitches reliant on shorter ultraviolet (UV) wavelengths are less suitable for underwater switching. However, the actuation wavelength of the new polymer aligns perfectly with this window. An additional noteworthy characteristic of water is its cooling effect, whereby its natural heat convection transfer coefficient surpasses that of air by a factor of 100 to 200.^[^ ^53^ ^]^ Consequently, it is reasonable to anticipate isothermal conditions in an underwater environment. This supposition finds support in the analyses conducted by Schenning and colleagues,^[^ ^54^ ^]^ who investigated the bending of photomechanical liquid crystal networks in both air and water. Their findings confirm the presence of photomechanical rather than photothermal effects when actuation occurs underwater. To explore underwater switching, a polymer film with a thickness of 60 µm and splay alignment was immersed in water at 21 °C within an elongated glass cuvette, with the homeogeneous side facing the respective light sources (see Figure 9 and Video [Link], [Link], Supporting Information). Illumination with collimated 525 nm light, propagating through water for a distance of ≈ 3 cm, induced bending toward the light at a rate of 0.65° s^−1^. The final bending angle reached 32°, which slightly falls short of the bending rate and angle observed in air (as described above). The bent shape of the polymer film remained intact until illuminated with 420 nm light (collimated source, 40 (low focused beam) up to 200 mW cm^−2^ (high focused beam) (intensity measured max. 120 mW cm^−2^ for 420 nm and max. 140 mW cm^−2^ for 525 nm, traversing water for ≈ 3 cm). Upon exposure to this light, the polymer film returned to its original shape, demonstrating complete reversibility.

However, when subjected to UV light, no discernible bending occurred. This underpins the importance of the wavelength dependent depth of penetration in different media.

Furthermore, a large bending motion was also observed by irradiation with orange light (590 nm). These experiments underscore the remarkable utility of tetrafluorinated LC photoswitches in the film for underwater actuation and provide evidence for a genuinely photomechanical and non‐photothermal effect in water, with only a minor contribution from photothermal effects in air.

It is known that the macroscopically observed relaxation of an azobenzene containing LCN can be very different from the actual half‐life time of the azobenzenes.^[^ ^15^ ^]^ Therefore, the thermal relaxation of the *Z*‐tetrafluoroazobenzene in the polymer film **P_planar,31_** was measured by UV–vis spectroscopy (For the spectra see the Supporting Information). Because the films consist solely of the azobenzene monomer, they are highly absorbing and only the thinnest film could be measured (for photographic images of the films, as well as the effect of a polarizer see Supporting Information). In addition, only a homogenous alignment was possible, because of the intransparence of the splay aligned films. Because the amount of switched monomer will be a function of the penetration depth of light, the local dielectric constant in the polymer film will have a depth profile. Also, the stiffness of the polymer will have a depth profile. Therefore, the thermal decay of the monomer is unlikely to follow a first‐order kinetic and a thermal half‐life cannot be given with any precision. From the decay profile, we can merely estimate that the half‐time is roughly 17 h.

In this respect, this new LCN is unusual: It has been reported that the thermal *Z* to *E* isomerization is much slower than the mechanical deformation (hours vs seconds).^[^ ^15^ ^]^ However, in our case, thermal relaxation appears to be somewhat faster, although not by orders of magnitude. It also has to be considered that the macroscopic relaxation was observed with the **P_splay,60_** film, whereas the UV–vis measurement was performed with the **P_planar,31_** film.

The splay LCN films reported in this work **P_splay,60_** and **P_splay,105_** are thicker in comparison to the UV‐light actuated analogs. This limitation of thickness is a direct function of the wavelength penetration depth of light in matter. This is a function of absorbance, but also scattering. In azobenzene, isomerization to the *Z*‐isomer is typically affected by irradiating in the UV range, exciting the allowed π‐π* transition which has a large extinction coefficient (2.6 × 10^4^ M^−1^cm^−1^). Due to this and the fact that light with shorter wavelengths scatters more, it was postulated that in azobenzene containing thin polymer films, most photons are absorbed within a thickness of >1 µm for UV‐light irradiation.^[^ ^26b^ ^]^ However, in tetra‐*ortho* fluorinated azobenzene, the longer wavelength n‐ π* transitions are well separated and have a higher absorption coefficient so that they can be used instead for the isomerization. The longer wavelength combined with the lower extinction coefficient for the n‐π absorbance (1 M^−1^cm^−1^),^[^ ^32c^ ^]^ may lead to a deeper penetration depth of the green light, which may enable bending the motion in thicker samples. Indeed, the UV–vis spectra for the polymer film **P_planar,31_** showed that the film was intransparent in the UV‐region, but spectra were observable in the range from 400–700 nm (see Supporting Information).

**Figure 10:**
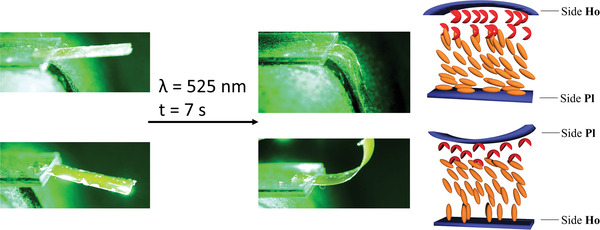
Photobending of **P_Splay,40_** in air. The polymer stripe was clamped between two microscope slides. Pictures taken from the video after 7 s of irradiation with the schematic representation of the actuation within the material on the right. For a video of the switching process see supporting video Video S4 (Supporting Information).

Because this work eventually aims at introducing visible light switchable LCNs to the area of soft robotics, in addition to the narrow polymer film strips, a larger piece of a splay aligned polymer film **P_Splay,40_** with dimensions of 2.5 cm × 1.5 cm 40 µm was prepared. This film could be bent within 7 s to an angle of 90 ° with a remarkably low light intensity of 10 mW cm^−2^ (**Figure** **10**, Video S4, Supporting Information). During illumination with green light for 30 s, the temperature in the film, observed with an IR camera, increased to 37.4 °C and cooled down within seconds after illumination (see Supporting Information).

## 4 Conclusion

A novel synthetic design for the first tetra‐*ortho*‐fluorinated liquid crystal azobenzene monomer capable of reversible switching solely with visible light in both directions has been introduced. This was made possible through the tetra‐*ortho* fluorination of the azobenzene combined with the expansion of the rigid core of the mesogen. The monomer exhibits a nematic phase over a wide temperature range (32 K), as confirmed by comprehensive studies employing DSC, POM, and WAXS techniques. The monomer was polymerized in a hybrid nematic aligned liquid crystal cell, resulting in films with splay alignment and thicknesses of 40, 60, and 105 µm. These films exhibit a glass transition temperature of 60 °C. WAXS measurements performed on a model film with homogeneous planar alignment revealed that polymerization increased the molecular order, transitioning to a smectic C phase. The splay aligned film with a thickness of 60 µm demonstrated reversible photomechanical actuation when irradiated alternately with violet and green light. Remarkably, despite the film's significant thickness and the relatively low irradiation power ranging from 40 to 200 mW cm^−^^2^, the bending occurred with substantial changes in angle (40°) and at high speed (1° s^−1^). This can be attributed to the longer wavelengths of the light sources penetrating deeply into the film, the high monomer concentration (100%), and the dense cross‐linking. Additionally, the bent shape exhibited a thermal shape stability over several days. Underwater conditions only slightly affected the bending angle and rate, suggesting that for a thickness of 60 µm, the photothermal effect is minimal, and the bending process is primarily driven by photomechanical mechanisms.

To summarize, this study presents a robust photomechanical liquid crystal network (LCN) capable of actuation with green light, avoiding the use of potentially harmful UV light. This material holds significant potential for applications in biosafe soft robotics that require precise control. Moreover, the synthesis process offers high yields, while film preparation is straightforward, ensuring the accessibility of this material.

## 5 Experimental Section

### General Synthetic and Analytical Methods

For all equipment, syntheses, fabrication processes, images and spectra see the Supporting Information.

### Preparation of the LCN

In a glass vial, **5** and the photocatalyst (3 wt.%) were dissolved in DCM. The solvent was removed in vacuo. The polymerizations were performed on a Linkam LTS‐420 heating stage in between PI coated glass slides. The glass slides were spin‐coated with a 5 wt.% PI (P84, Elsinger) solution in NMP and dried for 1 h at 100 °C and another 24 h at 200 °C in a drying oven. One of the glass slides were rubbed with a velvet cloth along one axis before use. A stripe of a 10, 30, or 50 µm thin PTFE film was used as a spacer to form the LC cell. The LC cell was glued together by the UV‐curing acrylate adhesive NOA61 from Norland Optical Adhesive. The monomer mixture was inserted into the LC by capillary force at 155 °C (isotropic phase). Subsequently, the temperature was slowly lowered (0.5 K min^−1^) to the nematic phase (130 °C). This temperature was held for 30 min. Afterward, a LED with a wavelength of 420 nm was used to irradiate the LC cell through a linear polarizer orthogonally to the planar alignment of the LC cell for 12 h to activate the photocatalyst without switching the azobenzene. Additionally, a PMMA slide was placed between the light source and the LC cell to block irradiation in the UV‐range. Then, the temperature was raised to 155 °C again and hold for 15 min. The lamp was turned off and the temperature hold for another 15 min, before cooling down to room temperature and carefully opening the LC cell using a razor blade. To facilitate the extraction of the thin film, the razor blade and the LC cell were periodically immersed in hot water. The final thickness of the films was measured using a digital caliper

Further information can be found in the supporting information: equipment, syntheses, spectra, crystallographic data, further images.

(*E*)−4,4′‐(Diazene‐1,2‐diyl)bis(3,5‐difluorobenzoyl chloride) (**4**)


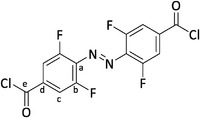


This reaction was performed under inert conditions.

In an oven dried flask, (*E*)−4,4′‐(diazene‐1,2‐diyl)bis(3,5‐difluorobenzoic acid) (**S7**) (1.03 g, 3.00 mmol, 1.00 equiv.) was dissolved in excess SOCl_2_ (5 mL). Then, a single drop of dry DMF was added and the reaction mixture heated up to 80 °C for 17 h. Afterward, the solvent was removed in vacuo by adding a cooling trap to the apparatus. The crude product was transferred to the glovebox and crystallized in dry toluene at −20°C. After filtration, the dark red product **4** was obtained in 96% (1.09 g, 2.88 mmol, 96%) yield.

**^1^H NMR** (600 MHz, CDCl_3_) *δ* = 7.86 (d, ^3^ *J* = 8.4 Hz, 4H, *H*‐c) ppm.

**^13^C{^1^H} NMR** (151 MHz, CDCl_3_) *δ* = 165.92 (*C*‐e), 155.06 (dd, ^2^ *J* = 265.0, ^4^ *J* = 3.8 Hz, *C*‐b), 136.05 (t, ^4^ *J* = 9.1 Hz, *C*‐d), 135.54 (t, ^3^ *J* = 10.4 Hz, *C*‐a), 115.68 (dd, ^2^ *J* = 22.1, ^4^ *J* = 4.4 Hz, *C*‐c).

**^19^F NMR** (565 MHz, CDCl_3_) δ = −117.46 (d, ^3^ *J* = 8.4 Hz, *F*‐b) ppm.

**HRMS** (EI, 70 eV) *m/z* (%): [M]^+^ calcd for [C_14_H_4_N_2_O_4_F_4_]^+^ 377.95801; found 377.95805 (10), 86.1 (100).

**IR** (ATR): *ṽ* = 1746 (m), 1698 (m), 1684 (w), 1576 (s), 1558 (m), 1540 (w), 1473 (w), 1431 (m), 1418 (m), 1396 (w), 1328 (w), 1260 (w), 1123 (m), 1054 (s), 1004 (s), 891 (m), 878 (w), 807 (m), 772 (w), 701 (m) cm^−1^.

(*E*)−4,4′‐(Diazene‐1,2‐diyl)bis(3,5‐difluorobenzoic acid) (**5**)


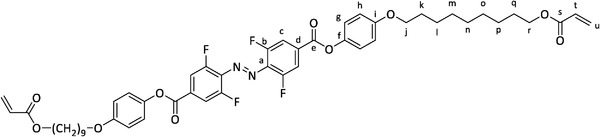


This reaction was performed under inert conditions.

In a glovebox, (*E*)−4,4′‐(diazene‐1,2‐diyl)bis(3,5‐difluorobenzoyl chloride) (**4**) (342 mg, 1 mmol, 1 equiv.) and 9‐(4‐hydroxyphenoxy)nonyl acrylate **(2)** (674 mg, 2.20 mmol, 2.20 equiv.) and triethylamine (1.0 mL) were dissolved in toluene (30 mL). The reaction was heated to 110 °C for 24 h. Then, the solvent was removed in vacuo and the crude product was directly purified by column chromatography (eluent gradient: Hexane → DCM) to afford **5** as an orange solid. (707 mg, 0.769 mmol, 77%)

**^1^H NMR** (600 MHz,CDCl_3_) *δ* = 7.91 (d, ^3^ *J* = 9.3 Hz, 4H, *H*‐c), 7.14 (d, ^3^ *J* = 9.0 Hz, 4H, *H*‐g), 6.95 (d, ^3^ *J* = 9.0 Hz, 4H, *H*‐h), 6.40 (dd, ^2^ *J* = 17.4, ^4^ *J* = 1.5 Hz, 2H, *H*‐u), 6.13 (dd, ^3^ *J* = 17.4, ^3^ *J* = 10.4 Hz, 2H, *H*‐t), 5.82 (dd, ^3^ *J* = 10.4, ^4^ *J* = 1.5 Hz, 2H, *H*‐u), 4.16 (t, ^3^ *J* = 6.7 Hz, 4H, *H*‐j), 3.97 (t, ^3^ *J* = 6.5 Hz, 4H, *H*‐r), 1.83 – 1.77 (m, 4H, *H*‐q), 1.71 – 1.65 (m, 4H, *H*‐k), 1.48‐1.46 (m, 4H, *H*‐p), 1.37 (s, 16H, *H*‐l,m,n,o).

**^13^C{^1^H} NMR** (151 MHz,CDCl_3_) *δ* = 166.51 (*C*‐s), 162.84 (*C*‐e), 157.43 (*C*‐f), 155.24 (d, ^1^ *J* = 263.4 Hz, *C*‐b), 143.88 (*C*‐i), 134.71 (t, ^2^ *J* = 10.0 Hz, *C*‐a), 133.13 (t, ^3^ *J* = 9.5 Hz, *C*‐d), 130.61 (*C*‐u), 128.80 (*C*‐t), 122.21 (*C*‐g), 115.36 (*C*‐h), 114.65 (dd, ^2^ *J* = 21.7, ^4^ *J* = 4.0 Hz, *C*‐c), 68.56 (*C*‐r), 64.83 (*C*‐j), 29.57 (*C*‐q), 29.43 (*C*‐l,m,n,o), 29.37 (*C*‐k), 29.32 (*C*‐l,m,n,o), 28.75 (*C*‐l,m,n,o), 26.16 (*C*‐l,m,n,o), 26.05 (*C*‐p).

**^19^F NMR** (565 MHz, CDCl_3_) δ = −118.92 (d, ^3^ *J* = 9.3 Hz, *F*‐b).

**HRMS** (ESI) *m/z* (%): [M+Na]^+^ calcd for [C_50_H_54_F_4_N_2_NaO_10_]^+^ 941.36068; found 941.36095.

**IR** (ATR): *ṽ* = 2923 (w), 1710 (m), 1575 (w), 1501 (w), 1473 (m), 1434 (w), 1336 (m), 1295 (m), 1204 (s), 1184 (s), 1110 (m), 1048 (m), 1017 (m), 988 (m), 968 (m), 958 (m), 912 (w), 898 (w), 827 (m), 812 (s), 773 (m), 886 (m), 752 (m), 725 (w) cm^−1^.

**Mp**: Cr120N153I

**R** *f*: 0.5 (*n*‐hexane:DCM, 1:1).

[CCDC 2 224 454 contains the supplementary crystallographic data for this paper. These data can be obtained free of charge from The Cambridge Crystallographic Data Centre via www.ccdc.cam.ac.uk/data_request/cif.]

## Conflict of Interest

The authors declare no conflict of interest.

## Supporting information

Supporting Information

Supplemental Video 1

Supplemental Video 2

Supplemental Video 3

Supplemental Video 4

Supplemental Video 5

## Data Availability

The data that support the findings of this study are available in the supplementary material of this article.
