# Supplementary material for: A Photomechanical Film in which Liquid Crystal Design Shifts the Absorption into the Visible Light Range
Source: Adv Sci (Weinh). 2023 Sep 3;10(30):2302692. doi: 10.1002/advs.202302692 (PMC10602558; doi:10.1002/advs.202302692)
Supplement: Supplementary file 1 — Supporting Information [file ADVS-10-2302692-s002.pdf]

ADVANCED SCIENCE
Open Access

Supporting Information

for *Adv. Sci.*, DOI 10.1002/advs.202302692

A Photomechanical Film in which Liquid Crystal Design Shifts the Absorption into the Visible Light Range

*Sven Schultzke, Nikolai Scheuring, Pim Puylaert, Matthias Lehmann and Anne Staubitz**

Supporting Information

A Photomechanical Film in which Liquid Crystal Design Shifts the Absorption into the Visible Light Range

Sven Schultzke [1,2], Nikolai Scheuring [3], Dr. Pim Puylaert [4], Prof. Matthias Lehmann [3],

Prof. Anne Staubitz [1,2,]*

Sven Schultzke, Prof. Anne Staubitz

[1] University of Bremen, Institute for Analytical and Organic Chemistry,

Leobener Straße 7, D-28359 Bremen, Germany

[2] University of Bremen, MAPEX Center for Materials and Processes,

Bibliothekstraße 1, D-28359 Bremen, Germany

[3] University of Würzburg, Institute of Organic Chemistry,

Am Hubland, D-97074 Würzburg, Germany

[4] University of Bremen, Institute for Inorganic Chemistry and Crystallography,

Leobener Straße 7, D-28359-Bremen, Germany

E-mail: staubitz@uni-bremen.de

* Correspondence: staubitz@uni-bremen.de; Tel.: +49421/21863210

Contents

1. General Information 2

1.2 Reagents 5

1.3 Solvents 5

2. Analytical Data 7

2.1 Switching Properties of **5** 7

2.1.1 UV-Vis Analysis: Qualitative Switching Behavior of **5** 7

2.1.2 Switching Properties of **5** 8

2.1.3 UV-Vis Spectra After the Irradiation of 5 with Different Wavelengths in Chloroform and Cyclohexane 9

2.1.4 NMR Analysis: Quantitative Switching Behavior of **5** 10

2.2 Liquid Crystal Analysis 11

2.2.1 DSC: Phase-Transitions of **5** up to 170 °C 11

2.2.2 TGA: Thermal Stability of **5** at the Polymerization Temperature (130 °C) 12

2.2.3 TGA: Thermal Stability and Decomposition of **5** 12
2.2.4 DSC: Phase-Transition of **S8** up to 200 °C 13
2.2.5 DSC: Phase-Transition of **6** up to 250 °C 14
2.2.6 Crystal Structure Analysis of **6** 15
2.3 Analysis of the Polymer 18
2.3.1 DSC: Phase-Transition of $\mathbf{P_{Splay,105}}$ up to 200 °C 18
2.3.2 WAXS Measurements of the Homogeneously Aligned Polymer Film $\mathbf{P_{Planar,49}}$ 19
2.3.3 UV-Vis Analysis with Different Wavelength Irradiation of $\mathbf{P_{Planar,31}}$ 20
2.3.4 Photochemical Bending of $\mathbf{P_{Splay,60}}$ by Irradiation 20
2.3.5 Bending of $\mathbf{P_{Splay,60}}$ and Relaxation in the Dark 22
2.3.6 UV-Vis Analysis: Relaxation of $\mathbf{P_{Planar,31}}$ after Irradiation with 525 nm 22
2.3.7 UV-Vis Analysis: Relaxation of $\mathbf{P_{Planar,31}}$ after Irradiation with 525 nm Determined at 440 nm 23
2.3.8 IR-Analysis: Thermal Heating during Irradiation of $\mathbf{P_{Splay,40}}$ and $\mathbf{P_{Splay,60}}$ 24
3.1. Comparison of the Transparency Depending on the Alignment and Thickness 26
4. Syntheses 28
4.1 9-Bromononyl acrylate (**S1**) 28
4.2 9-(4-hydroxyphenoxy)nonyl acrylate **(2)** 28
4.3 4-Bromo-2,6-difluoroaniline (**S2**)[5] 29
4.4 4-Amino-3,5-difluorobenzonitrile (**S3**) [6] 29
4.5 4-Amino-3,5-difluorobenzoic acid (**S4**)[6] 30
4.6 Ethyl 4-amino-3,5-difluorobenzoate (**S5**)[6] 30
4.7 (*E*)-4,4'-(Diazene-1,2-diyl)bis(3,5-difluorobenzoic acid) (**S6**)[9] 31
4.8 (*E*)-4,4'-(Diazene-1,2-diyl)bis(3,5-difluorobenzoic acid) (**S7**)[6] 32
4.9 (*E*)-4,4'-(Diazene-1,2-diyl)bis(3,5-difluorobenzoyl chloride) (4) 32
4.10 (*E*)-4,4'-(Diazene-1,2-diyl)bis(3,5-difluorobenzoic acid) (**5**) 33
4.11 Bis(9-(acryloyloxy)nonyl) 4,4'-(diazene-1,2-diyl)(*E*)-bis(3,5-difluorobenzoate) (**S8**) 34
4.12 Bis(4-hexylphenyl) 4,4'-(diazene-1,2-diyl)(E)-bis(3,5-difluorobenzoate) (**6**) 34
5. References 35
6. ^{1}H, ^{13}C{^{1}H} and ^{19}F NMR Spectra of the Purified Compounds 36
7. Emission spectra of the LED´s 54
8. Crystallographic Analysis 58

1. General Information

For reactions under inert conditions, a nitrogen filled glovebox (Pure Lab^{HE} from Inert, Amesbury, MA USA) and standard Schlenk techniques were used.

All glassware was dried in an oven at 200 °C for several hours prior to use. NMR tubes were dried in an oven at 110 °C for several hours prior to use.

NMR spectra were recorded on a Bruker Avance Neo 600 (Bruker BioSpin, Rheinstetten, Germany) (600 MHz (^{1}H), 151 MHz (^{13}C{^{1}H}), 565 MHz (^{19}F) at 298 K. All ^{1}H NMR and ^{13}C{^{1}H} NMR spectra were referenced to the residual proton signals of the solvent (^{1}H) or the solvent itself (^{13}C{^{1}H}). ^{19}F NMR spectra were referenced internally against trichlorofluoromethane. The exact assignment of the peaks was performed by two-dimensional NMR spectroscopy such as ^{1}H COSY, ^{13}C{^{1}H} HSQC and ^{1}H/^{13}C{^{1}H} HMBC when possible.

High-resolution EI mass spectra were recorded on a MAT 95XL double-focusing mass spectrometer from Finnigan MAT (Thermo Fisher Scientific, Waltham, MA, USA) at an ionization energy of 70 eV. Samples were measured by a direct or indirect inlet method with a source temperature of 200 °C. High-resolution ESI and APCI mass spectra were measured by a direct inlet method on an Impact II mass spectrometer from Bruker Daltonics (Bruker Daltonics, Bremen, Germany).

IR spectra were recorded on a Nicolet i510 FT-IR spectrometer from Thermo Fisher Scientific (Thermo Fisher Scientific, Waltham, MA, USA) with a diamond window in an area from 500 – 4000 cm^{-1} with a resolution of 4 cm^{-1}. All samples were measured 16 times against a background scan.

Melting points were recorded on a Büchi Melting Point M-560 (Büchi, Essen, Germany) and are reported corrected.

UV-vis spectra were recorded with a resolution of 0.5-0.1 nm on a UV-2700 spectrometer from Shimadzu (Shimadzu, Kyoto, Japan) with a double monochromator. In all cases, chloroform (spectroscopy grade), toluene (spectroscopy grade) or cyclohexane (spectroscopy grade) were used as a solvent.

For the UV switching experiments LED lamps with 340 nm, 365 nm (Ocean Optics USB 4000, Sahlmann Photochemical Solutions, Bad Segeberg, Germany) full width at half maximum (FWHM) (10 nm, 1.0 W), 443 nm, 470 nm, 525 nm, 565 nm and 627 nm LED lamp (Ocean Optics USB 4000, Sahlmann Photochemical Solutions, Bad Segeberg, Germany, FWHM 19 nm, 0.9 W) were used, while a constant distance towards the cuvettes of 1 cm was ensured. The samples were measured immediately after irradiation. Additionally, a 420 nm (1.0 W) and a 525 nm LED (1.0 W) from EPILED was built in our laboratories. These additional LEDs were matching the absorbance of the azobenzene investigated in this paper and could be run for several hours for polymerization.

To facilitate the photochemical bending of the polymers, we employed LED modules provided by Thorlabs (Bergkirchen, Germany). These modules were powered by a UPLED power supply and focused using a collimator. The following high-power LED modules were utilized: M365L3 (365 nm), M415L4 (415 nm), M530L4 (530 nm), M590L4 (590 nm), and MWWHL4 (3000 K, "warm white"). The light intensity was measured using an ILT2400 hand-held light meter manufactured by International Light Technologies, Inc., (Peabody, USA). For linear polarization of the light, we used high contrast polarizing films (400-700 nm) obtained from Edmund Optics (York, UK). The polarization direction was determined using a polarization tester also provided by Edmund Optics.

Thermal analyses were performed on a standalone Mettler Toledo DSC 3+ STAR (Mettler-Toledo, Columbus, OH, USA) or a Mettler Toledo TGA/DSC 3+ System (Mettler-Toledo, Columbus, OH, USA), where 40 μL aluminum crucibles were used. In DSC experiments, pierced lids were used. A standard measurement of an azobenzene derivative included an isothermal treatment at 100 °C to for 60 min before running the thermal analysis. This ensured a full conversion to the *Z*-isomer of the azobenzene derivative. All analysis were run from -40° C up to the desired temperature. Thermo analytical data were analyzed with the STARe software (Version 16.00, Mettler-Toledo, Columbus, OH, USA) by Mettler Toledo and further plotted with the Origin Pro software. IR measurements were realized and recorded with a FLIR ONE Pro infrared camera. (Teledyne FLIR Systems, OR, USA).

WAXS investigations were performed on a Bruker Nanostar (Detector Vantec2000, Microfocus copper anode X-ray tube Incoatec). The compound was transferred to Mark capillaries, which were sealed and glued into a metal sample holder, where the liquid crystal phase was uniform aligned using ring magnets (1 Tesla). The XRS data were evaluated using the program Datasqueeze[1] with silver behenate as a calibration standard. The correlation lengths were estimated using the Scherrer formula.[2] The studies of optical textures of the mesophases were realized with a Nikon Eclipse LV100Pol optical polarizing microscope equipped with a Linkam LTS420 heating stage and a Linkam T95-HS system controller. Single crystal X-ray diffraction data were collected at 100 K using an open flow nitrogen stream on a Bruker Venture D8 diffractometer (Bruker, Karlsruhe, Germany) with a Photon 100 detector in shutterless mode with Mo-Kα (0.7107 Å) radiation. All structures were solved by intrinsic phasing and refined based on F^2 by use of the SHELX program package, as implemented in OLEX2[3] All non-hydrogen atoms were refined using anisotropic displacement parameters. Hydrogen atoms attached to carbon atoms were included in geometrically calculated positions using a riding model. The crystal was obtained by slow evaporation of an *n*-hexane/dichloromethane mixture at 20 °C.

Polymerization was performed on a Linkam LTS-420 heating stage in between PI coated glass slides. The glass slides were spin-coated with a 5 wt% PI solution in NMP and dried for 1h at 100 °C and another 24 h at 200 °C in a dry oven.
For splay alignment: One of the glass slides was rubbed along a one axis using a velvet cloth before use.
For homogeneous alignment: Both glass slides were rubbed along an axis using a velvet cloth before use and placed in an antiparallel configuration to avoid disturbance due possible pre-tilts of the mesogen.
For homeotropic alignment: No rubbing was performed, or an untreated clean glass substrate could be used.
To form the LC cell, a stripe of a 10, 30, or 50 µm thin PTFE film was used as a spacer. The LC cell was then glued together using the UV-curing acrylate adhesive NOA61 from Norland Optical Adhesive. The monomer mixture was introduced into the LC cell through capillary action in the isotropic phase (155 °C). Subsequently, the temperature was slowly decreased at a rate of 0.5 K/min until reaching the polymerization temperature (130 °C). This temperature was maintained for 30 minutes to allow mesogen alignment.
Afterwards, a LED with a wavelength of 420 nm was used to irradiate the LC cell through a linear polarizer that was orthogonal to the planar alignment. In this way, the photocatalyst could be activated without inducing azobenzene switching. Additionally, a PMMA slide was placed between the light source and the LC cell to block UV-range irradiation.
The final thickness of the films was measured using a digital caliper type 6012/1 (DIN 863, ±1 µm) from Richter Messwerkzeuge (95469 Speichersdorf, Germany). The alignment direction was assessed using a linear polarizer.

Thin layer chromatography (TLC) was performed using TLC Silica gel 60 F_{254} from Merck (Merck, Darmstadt, Germany) and the compounds were visualized by exposure to UV light at a wavelength of 254 nm. Column chromatography was performed manually using silica gel 60 (0.015-0.040 mm) from Merck (Merck, Darmstadt, Germany). The samples were applied via dry load with Celite® 503 (Macherey-Nagel, Düren, Germany) as column material. If stated, Celite® 503 was used as filtration aid.

The use of abbreviations follows the conventions from the ACS Style guide.[4]

1.2 Reagents

All chemicals were commercially available and used without purification unless stated otherwise.

Table S1. List of supplier and purity of used chemicals.

Reagent	Supplier, Purity	Comments
Acryloyl chloride	Acros Organics, 96%	stab. with 400 ppm phenothiazine
Bis[2,6-difluoro-3-(1-hydropyrrol-1-yl)phenyl]titanocene; 97%	Abcr, 97%	
9-Bromo-1-nonanol	Sigma-Aldrich, 95%	
N-Bromosuccinimide	Sigma Aldrich, 98%	
Copper (I) cyanide	TCI, >98.0%	
1,8-Diazabicyclo[5.4.0]undec-7-ene	Apollo Scientific, 99.7 (GC)	
4-Ethylaniline	Sigma-Aldrich, 98%	
d-Glucose	Sigma-Aldrich, 99.5%	
Hydroquinone	TCI, >99.0%	
N,N´-Dicyclohexycarbodiimide	Alfa Aesar, 99%	
4-Nitrobenzoic acid	Alfa Aesar, 99%	
Magnesium sulfate	VWR, 99.2%	
Oxone, monopersulfate	Abcr, -	
Potassium iodide	Abcr, 99.5%	
Polyimide Powder P84	Ensinger	Sample provided by Ensinger
Sodium carbonate	Chem Solute, 99.8%	
Sodium chloride	CarlRoth, >99.5%	
Sodium hydroxide	VWR, 98.6%	
Thionyl chloride	Acros Organics 99.7%	
Triethyl amine	Fluorochem, 99% (anhydrous)	degassed, stored in a glovebox

1.3 Solvents

All solvents for purification and extraction were used as received. All solvents used for synthesis under inert conditions were dried by a solvent purification system (SPS) from Inert Technologies.

Table S2. List of supplier and purity of used solvents.

Solvent	Supplier and Purity	Comments
Acetic Acid	Merck ≥99.8%	
Acetone	Sigma Aldrich	
Acetonitrile	Fisher Scientific 99.5%	
Aq. Ammonia (25%)	Merck, 25% aq. puriss. p.a.	
Benzene-d_6	Eurisotop, 99.5%	
Chloroform	VWR, 99.0% + 0.6 % EtOH (stab.)	
Chloroform-d_1	Euroisotop 99.8%	
Cyclohexane	Merck ≥99.5% (GC)	
DCM	Merck ≥99.9% (GC)	
DMF	Acros Organics 99.8%	Extra dry, AcroSeal™
DMSO	VWR technical grade	
DMSO-d_6	Sigma Aldrich, 99.9%	

Ethanol	VWR ≥99.8%	absolute
Ethyl acetate	Merck >99.5% (GC)	
Hydrochloric acid (37%)	Merck, fuming >37.0%	
Methanol	VWR >99.8% (GC)	
n-Hexane	Merck, >95%	
N-Methyl-2-pyrrolidone	Fisher Scietific, 99.5%	Extra dry, AcroSeal™
Sulfuric acid	Merck 95%	
Tetrahydrofuran	VWR HPLC grade	anhydrous from SPS, stored in a glovebox
Tetrahydrofuran	Fisher Scientific ≥99.8%	
Toluene	Merck >99.7% (GC)	
Toluene	Merck >99.7% (GC)	anhydrous from SPS, stored in a glovebox
Water	deionized	

2. Analytical Data

2.1 Switching Properties of **5**

2.1.1 UV-Vis Analysis: Qualitative Switching Behavior of **5**

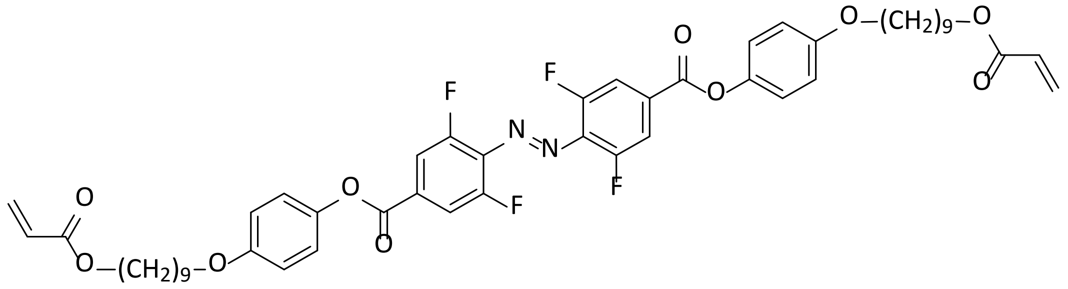

5

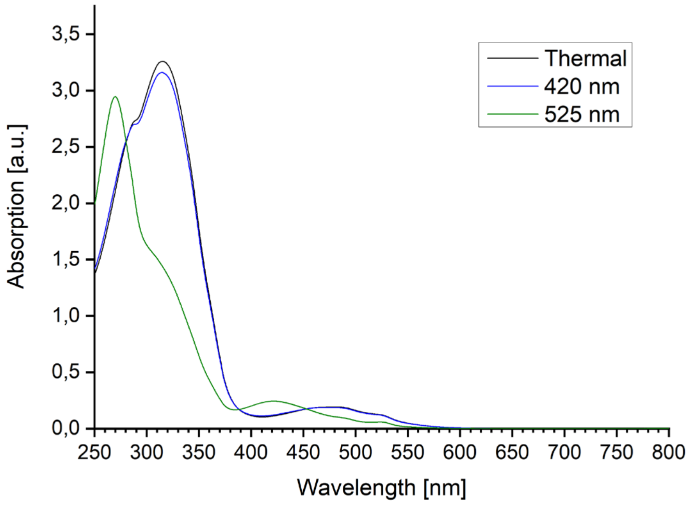

Figure S1. UV-vis spectrum of **5** in chloroform after thermal treatment and irradiation with green and violet light respectively.

2.1.2 Switching Properties of **5**

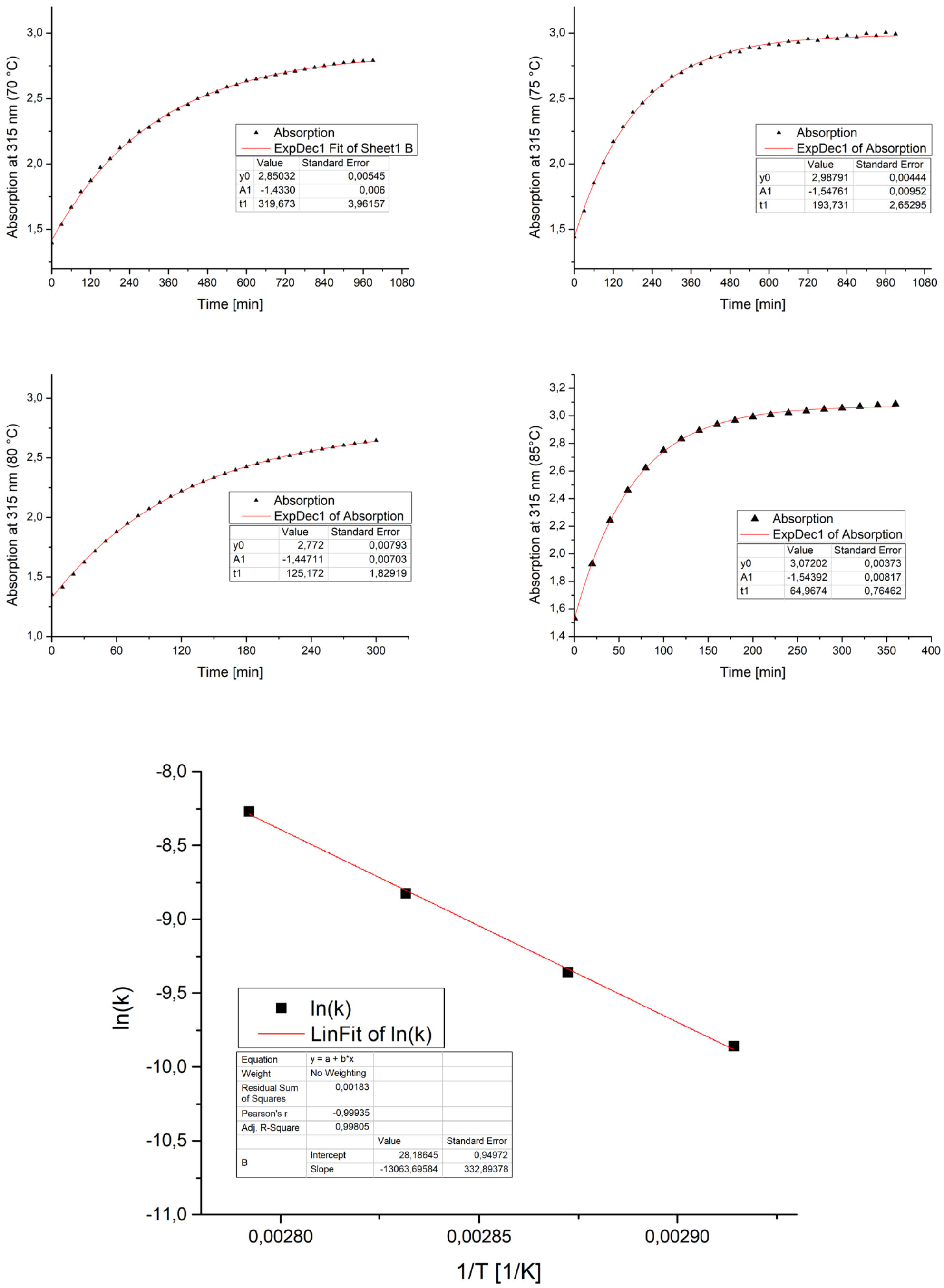

Figure S2. Half-life time measurement of **5** after irradiation with green light (525 nm) with a concentration of 120 µmol mL^{-1} in toluene. The half-life time for the thermal relaxation was determined at 85 °C, 80 °C, 75 °C and 70 °C by an exponential fit for the absorption at 315 nm. The half-life time for **5** at 25°C was then estimated by an Arrhenius-Plot based on the measurements at higher temperatures. The rate constant *k* was 1.63*10^{-7}s^{-1}, which translates to a half-life time $t_{1/2}$ of 49 d at 25 °C.

2.1.3 UV-Vis Spectra After the Irradiation of 5 with Different Wavelengths in Chloroform and Cyclohexane

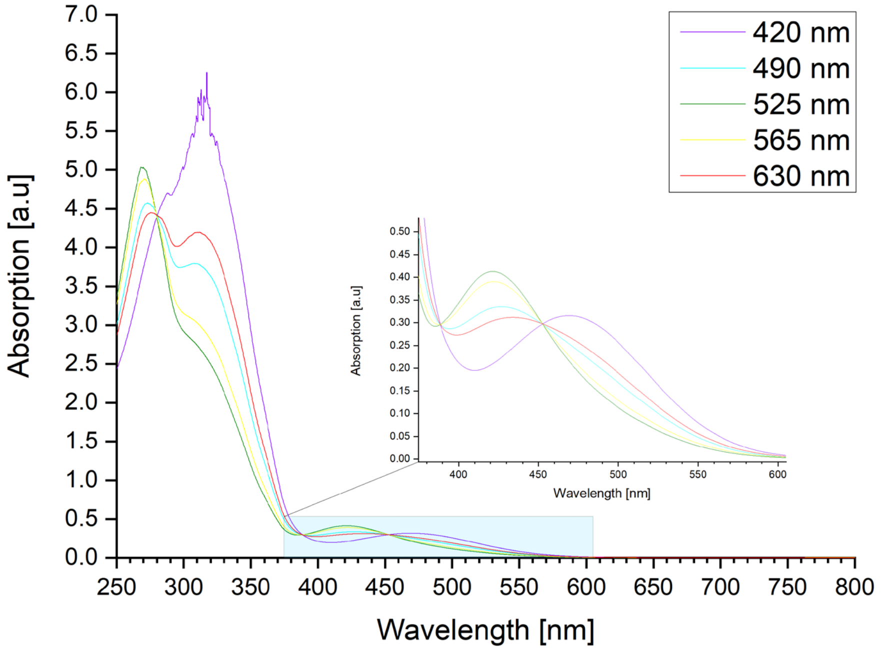

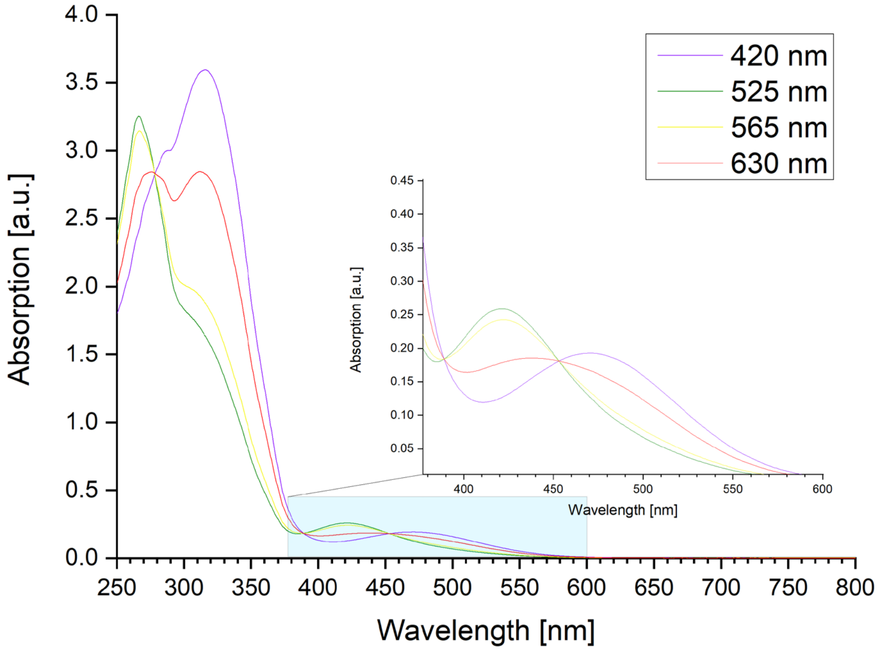

Figure S3. UV-vis spectrum of **5** in chloroform (top) and cyclohexane (bottom) after irradiation with different wavelengths for 1 min. Every illumination experiment was performed with a solution that had been brought into the PSS beforehand by illuminating it with 420 nm.

2.1.4 NMR Analysis: Quantitative Switching Behavior of **5**

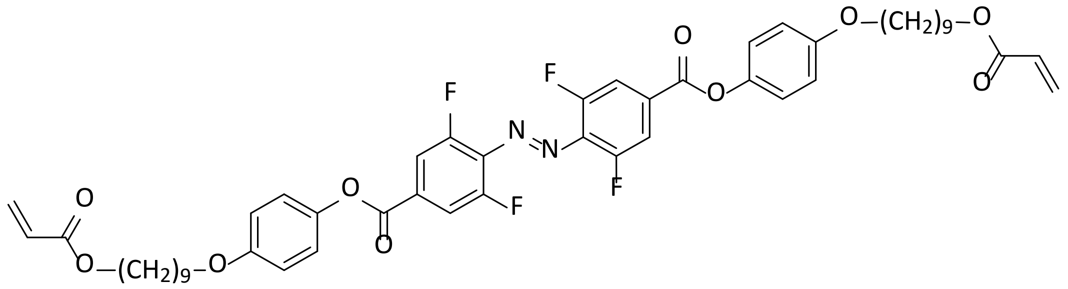

5

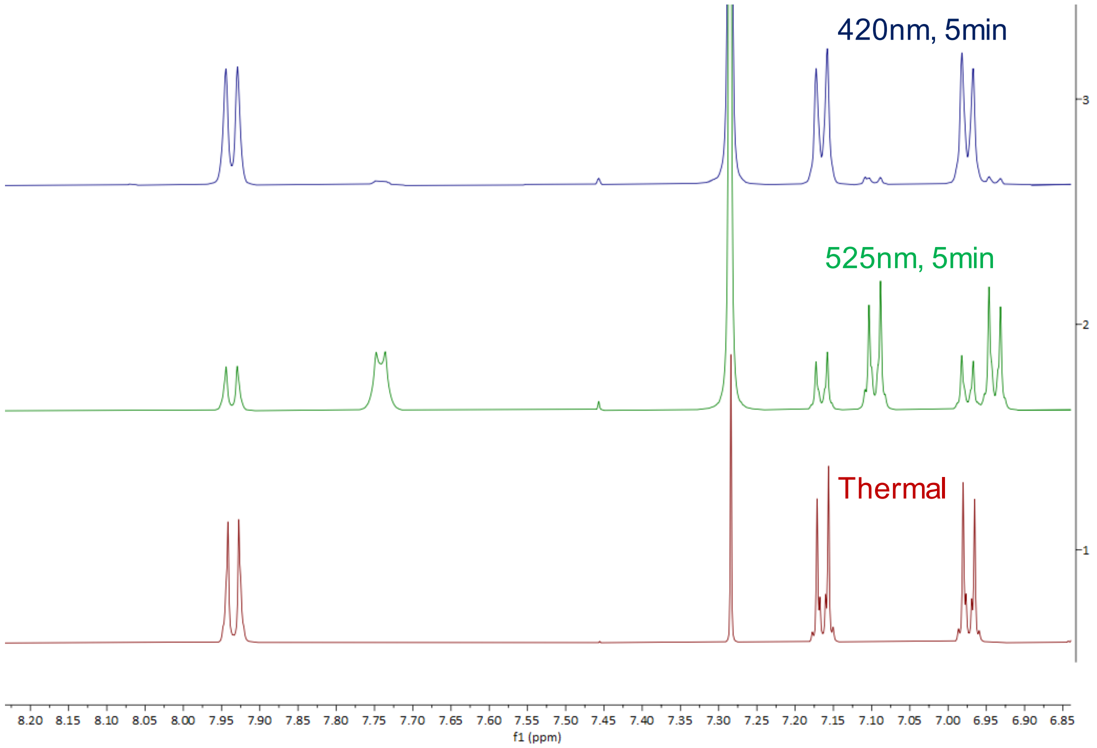

Figure S4. NMR spectroscopic study of the irradiation of **5** in $CDCl_3$ after thermal treatment* (red spectrum), after irradiation with green light (525 nm) and violet light (420 nm) for 5 min each.

Table S3. Summary of all NMR spectroscopic studies of the irradiation of **5**, after irradiation with different wavelengths in the solvents chloroform-*d* and benzene-d_6. The % values for the *E* and *Z* isomer were obtained by the integration of the aromatic proton signal.

	Chloroform-*d*		Benzene-d_6	
	E	*Z*	*E*	*Z*
**Thermal**	100%	0%	100%	0%
**420 nm**	93%	7%	92%	8%
**490 nm**	53%	47%	57%	43%
**525 nm**	30%	70%	30%	70%
**565 nm**	39%	61%	35%	65%

*Thermal treatment: Heating **5** up to 120°C for 24 h before dissolving the compound in the NMR solvent.

2.2 Liquid Crystal Analysis

2.2.1 DSC: Phase-Transitions of **5** up to 170 °C

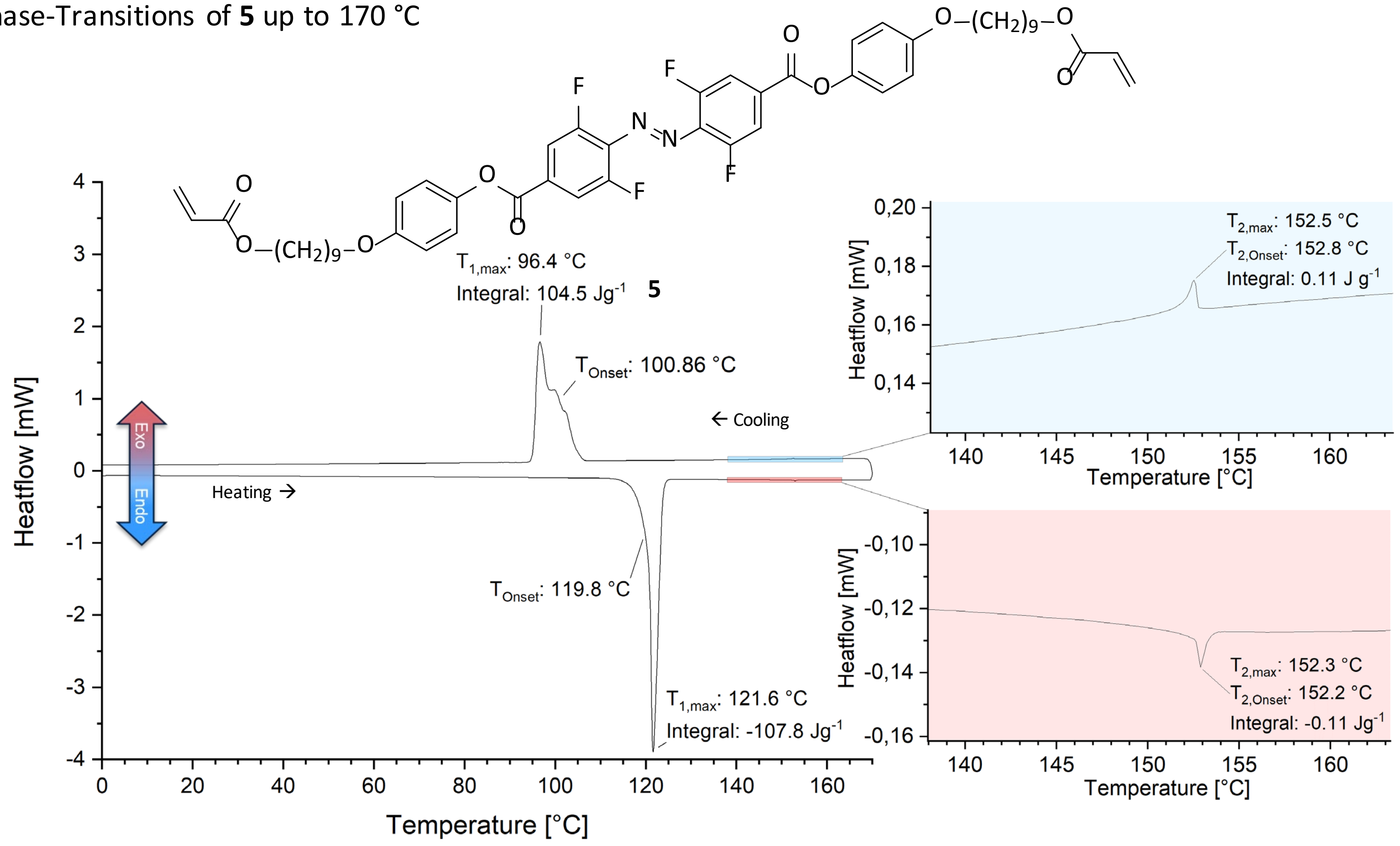

Figure S5. DSC curve of **5** (6.0481 mg) with a heating rate of 5 K/min for the temperature range -40 °C to 170°C. Right: Zoomed in area of the nematic to isotropic phase transition.

2.2.2 TGA: Thermal Stability of **5** at the Polymerization Temperature (130 °C)

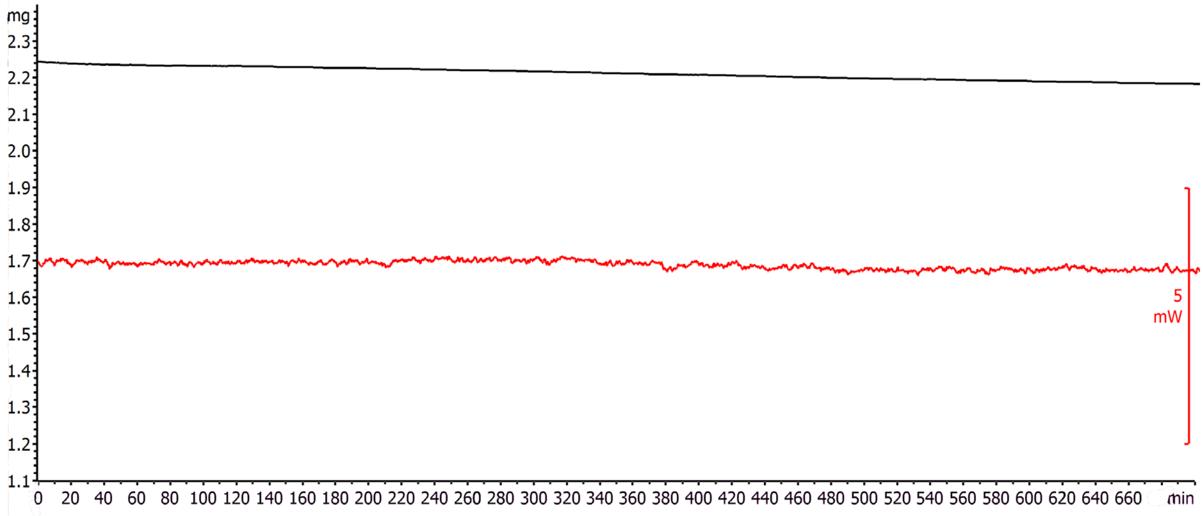

Figure S7. Isothermal TGA at 130 °C of **5** (2.26709 mg) in a 40 µL aluminum crucible under N_2 (20 mL min^{-1}) over 700 min.

An isothermal TGA experiment combined with a DSC analysis of the monomer **5** was performed. Over a time of 700 mins with a T of 130 °C and a gas flow of N_2 20 mL min^{-1} no appreciable weight loss occurred. Also, there was no significant change in the heat flow. From this, it can be concluded that the monomer **5** neither decomposes at this temperature nor that it polymerizes to any significant extent.

2.2.3 TGA: Thermal Stability and Decomposition of **5**

The monomer was thermally stable up to an onset temperature of 288 °C, at which temperature a weight loss step occurred, indicating decomposition of the sample.

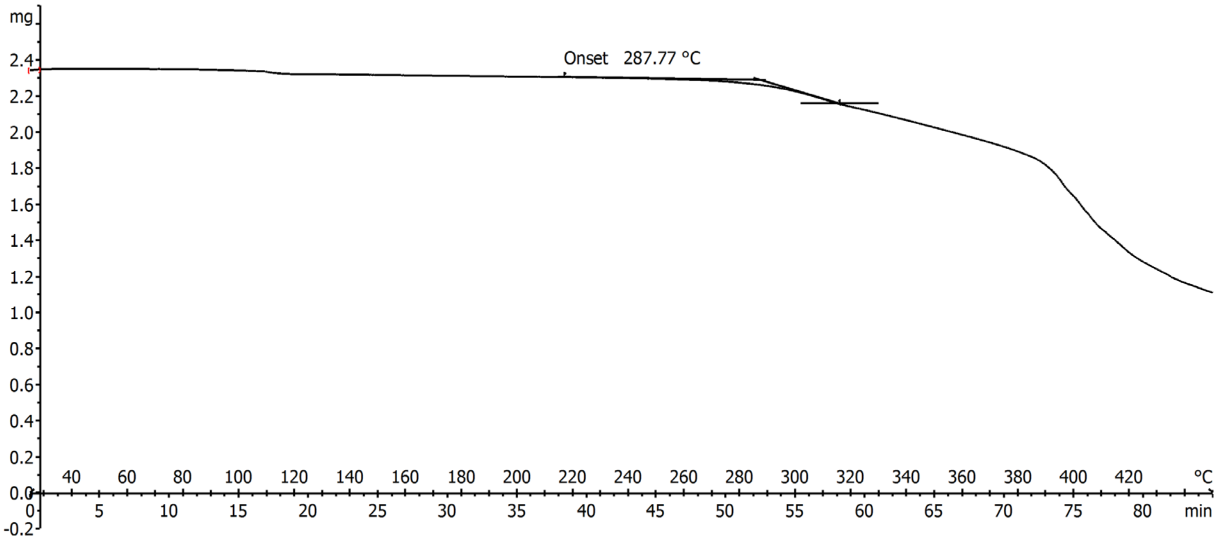

Figure S8. TGA Ramp with 5 K/min of **5** (2.34154 mg) in a 40 µL aluminum crucible under N_2 (20 mL/min) up to 450 °C.

2.2.4 DSC: Phase-Transition of **S8** up to 200 °C

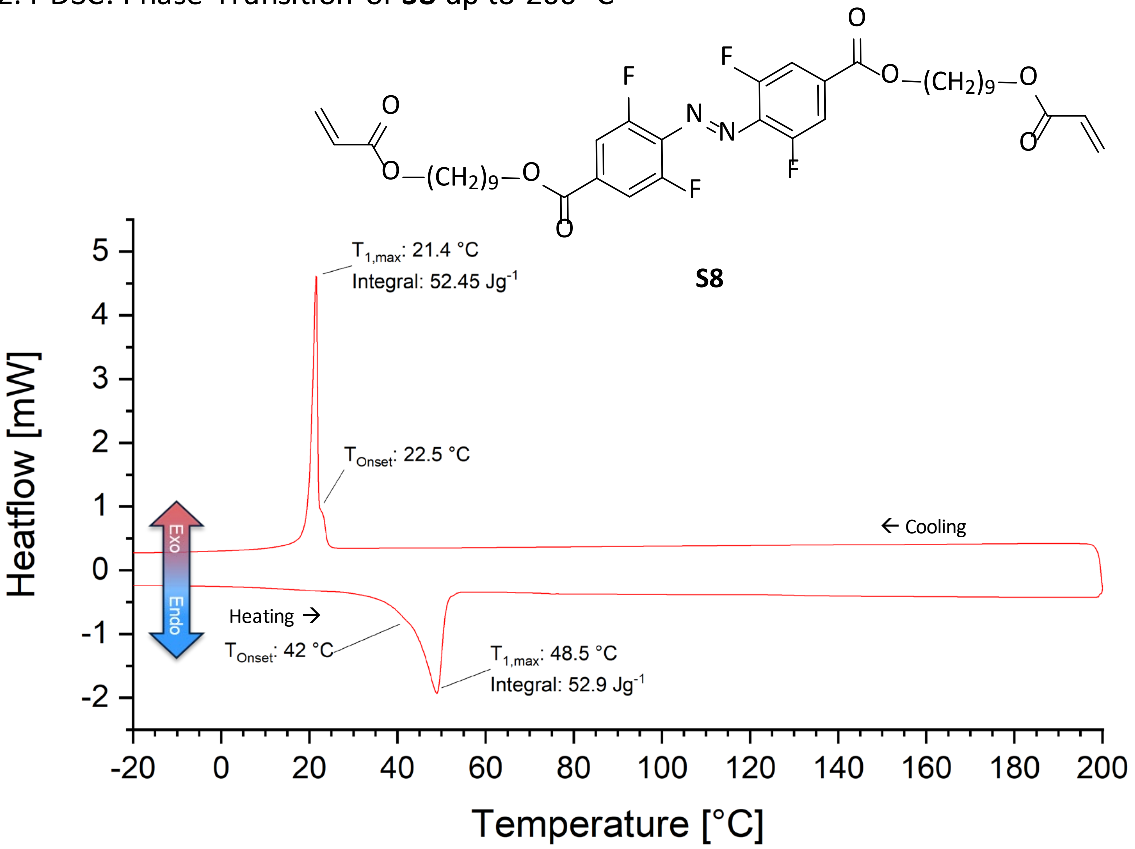

Figure S9. DSC curve of **S8** (3.5658 mg) with a heating rate of 5 K/min for the temperature range -40 °C to 200°C.

The monomer **S8** without the extended core showed (in comparison to monomer **5**) no signs of liquid crystallinity in the DSC (Figure S8) and POM (not shown).

2.2.5 DSC: Phase-Transition of **6** up to 250 °C

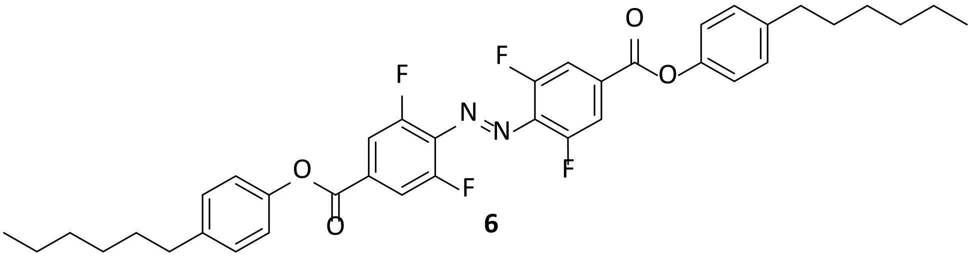

6

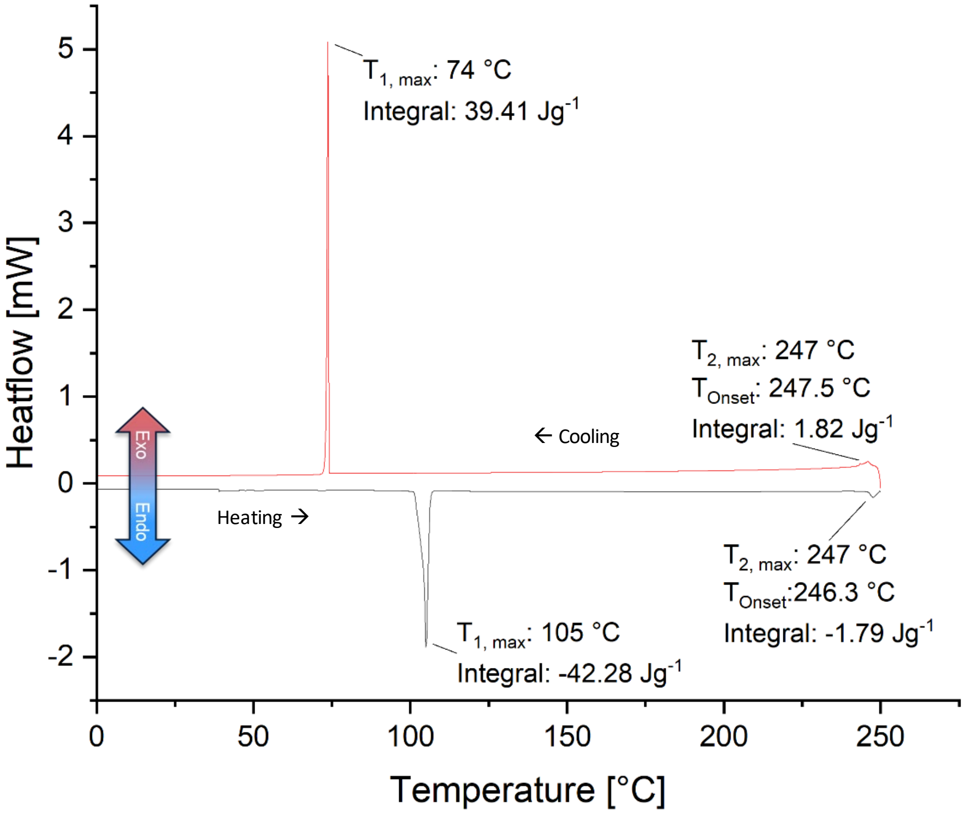

Figure S10. DSC curve of **6** (3.9546 mg) with a heating rate of 5 K/min for the temperature range -20 °C to 250°C for the second cycle.

2.2.6 Crystal Structure Analysis of **6**

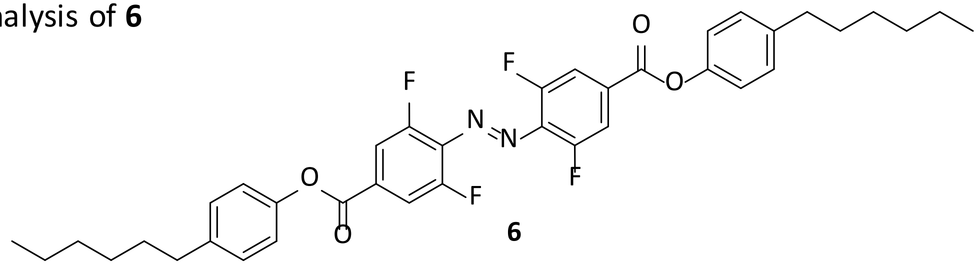

6

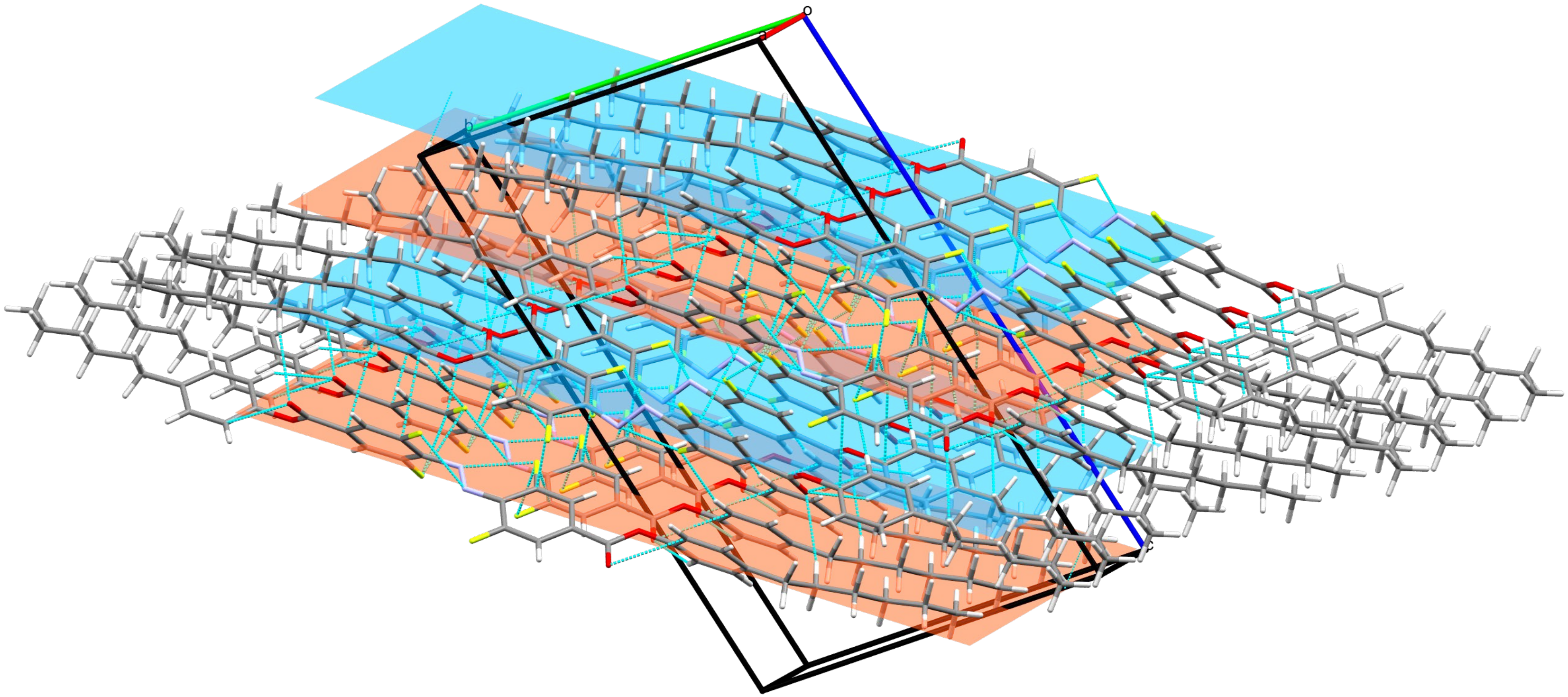

Figure S11. Crystal structure of **6** in its clustered alignment showing intermolecular interaction within the plane and between the levels marked in red and blue.

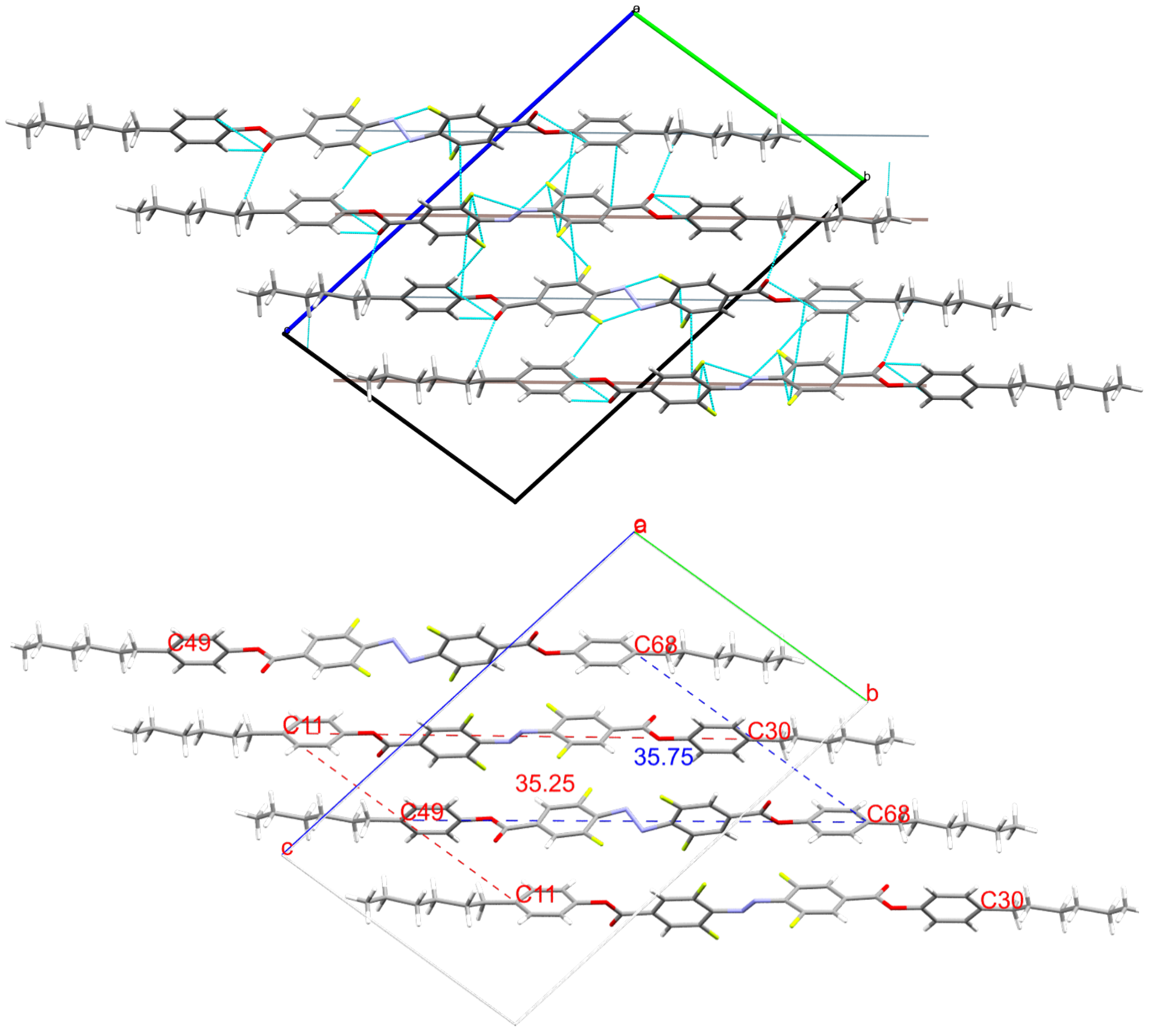

Figure S12. Crystal structure of **6** seen in the axis of the plane to distinguish the intermolecular forces between layers.(top) Tilt angle between the mesogens. As the mesogens show an internal torsion due to the fluorine atoms and thus is not entirely flat, the angle can only be defined between every second layer (marked in red and blue in **Figure S11**). As an approximation, the mesogen was modelled by a straight line from atom C30 to atom C11 and atom C49 to atom C68 respectively.(bottom)

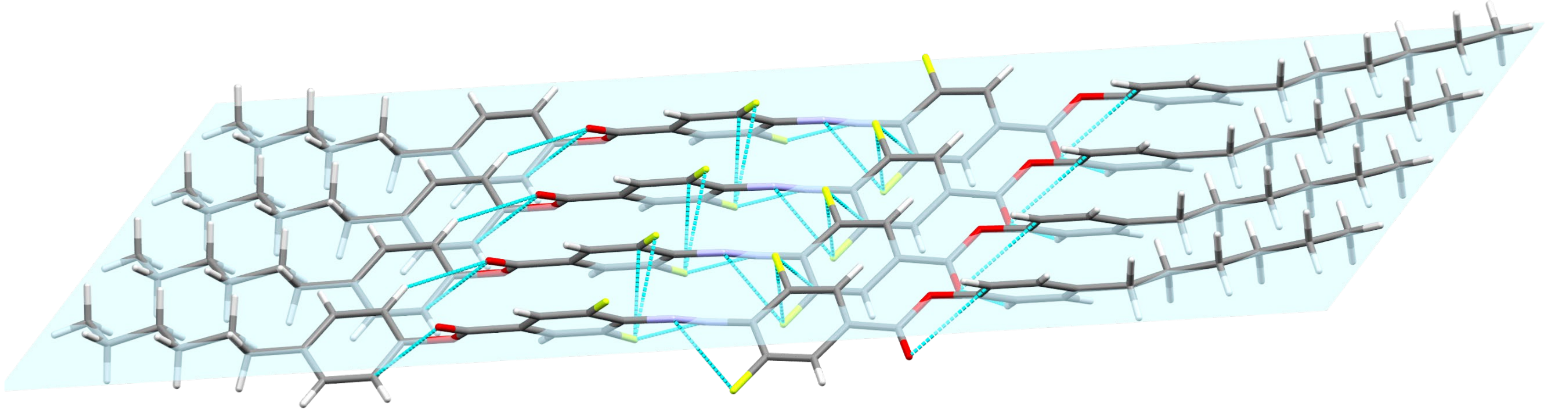

Figure S13. Crystal structure of **6** seen on top of a single plane to distinguish the intermolecular forces within the layer.

2.3 Analysis of the Polymer

2.3.1 DSC: Phase-Transition of $\mathbf{P_{Splay,105}}$ up to 200 °C

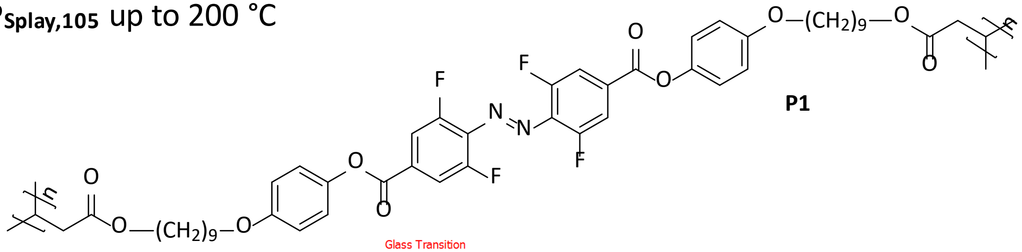

P1

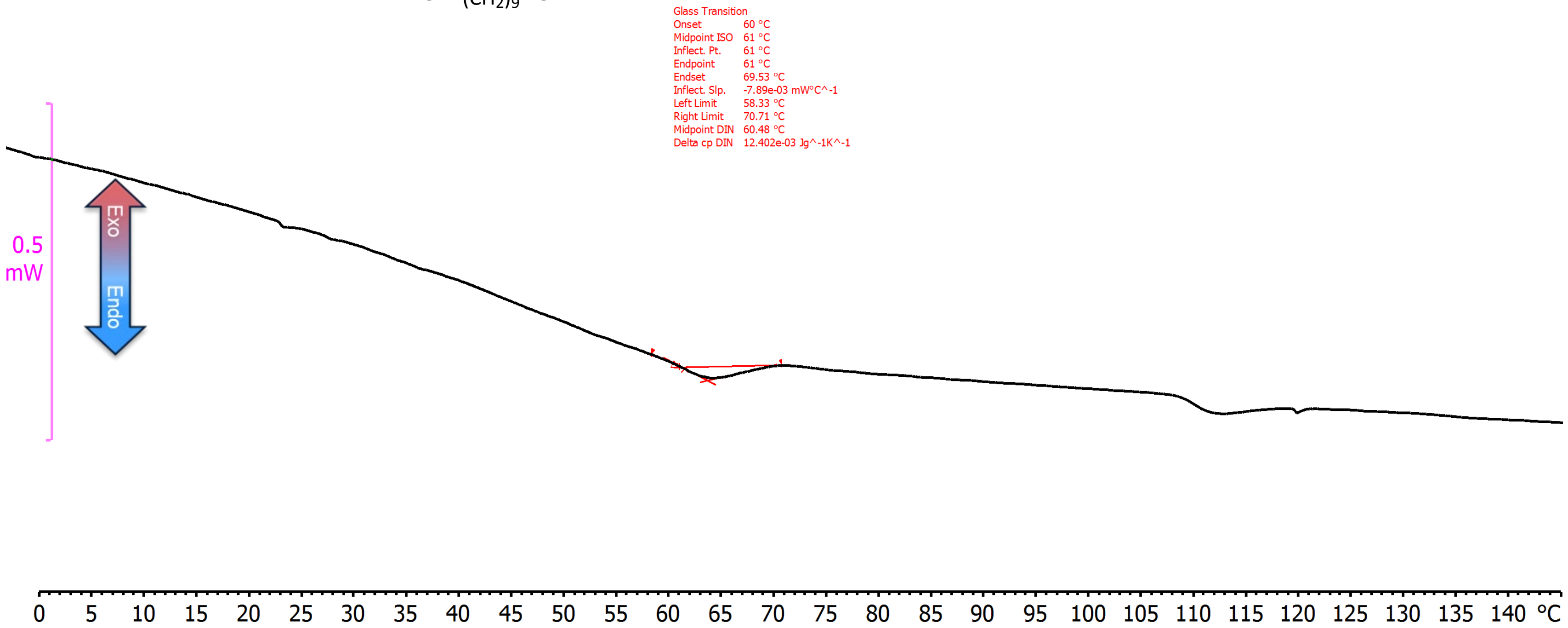

Figure S14. DSC curve of $\mathbf{P_{Splay,105}}$ (3.3714 mg) with a heating rate of 10 K/min for the temperature range -40 °C to 170°C.

2.3.2 WAXS Measurements of the Homogeneously Aligned Polymer Film $\mathbf{P}_{\mathbf{Planar,49}}$

Temperature-dependent WAXS studies of the $\mathbf{P}_{\mathbf{Planar,49}}$ polymer film reveal that the synclinic SmC phase remains stable within the examined temperature range, up to 93 °C (**Figure S15**). As anticipated, the structure undergoes only very slight changes, characterized by a reduction in the lamellar distance and an increase in the intermolecular distances: c (25°C) = 46.0 Å; c (93 °C) = 45.5 Å; dhalo (25°C) = 4.2 Å; dhalo (93°C) = 4.3 Å; dππ (25°C) = 3.4 Å; dππ (93°C) = 3.5 Å. The results were also obtained for the polymer film $\mathbf{P}_{\mathbf{Planar,31}}$ and $\mathbf{P}_{\mathbf{Planar,80}}$.

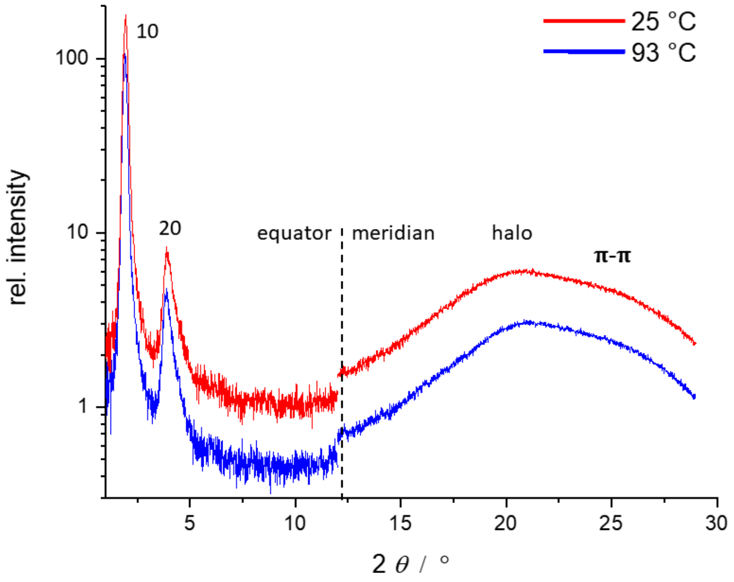

Figure S15. WAXS measurement of the polymer $\mathbf{P}_{\mathbf{Planar,49}}$ at 25 °C and 93 °C.

2.3.3 UV-Vis Analysis with Different Wavelength Irradiation of $\mathbf{P}_{\mathbf{Planar,31}}$.

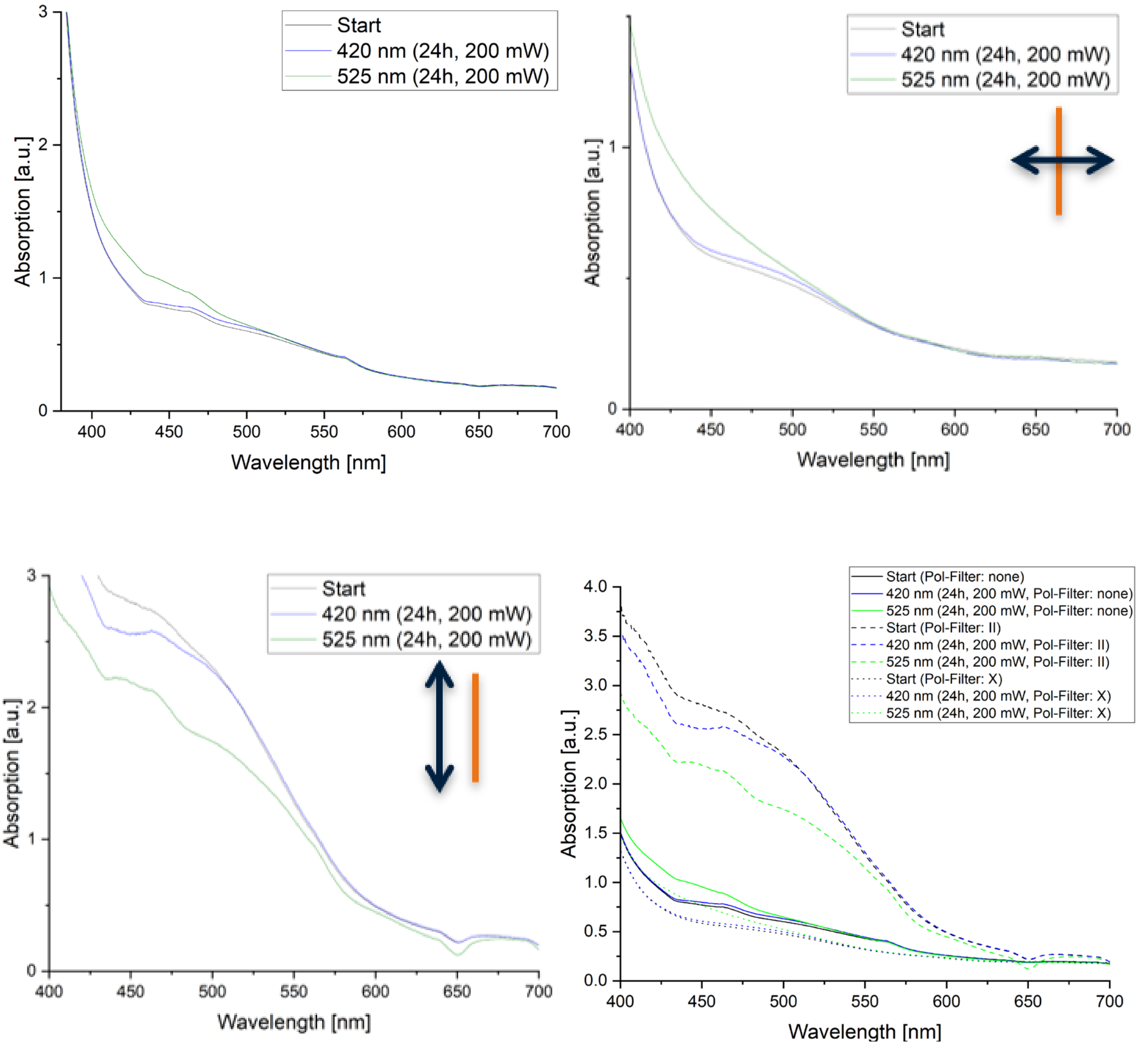

Figure S16. UV-vis spectra of the homogenously aligned $\mathbf{P}_{\mathbf{Planar,31}}$ (2 cm x 1cm x 40 µm); after irradiation with green light (525nm, 200 mW cm^{-2}) for 24 h; and afterwards violet light (420 nm, 200 mW cm^{-2}) irradiation for another 24 h. Each UV-Vis-spectrum was measured three times: With no polarization (top left), a linear polarization filter (400-700 nm) orthogonal ("X", top right), and a linear polarization filter parallel to the orientation ("II", bottom left) of the mesogen orientation. A combined UV-Vis spectrum is shown on the bottm right.

For the measurement, the $\mathbf{P}_{\mathbf{Planar,31}}$ was taped on a cuvette holder with Kapton tape. The tape was not placed in the pathway of the light beam. The linear polarization filter was taped in front of the light beam of the sample and the reference. A baseline was taken after the installment of the polarizer.

2.3.4 Photochemical Bending of $\mathbf{P}_{\mathbf{Splay,60}}$ by Irradiation

The polymer stripe $\mathbf{P}_{\mathbf{Splay,60}}$ (1.5 cm x 0.2 cm x 60 µm) was irradiated with green light (525 nm) and violet light (420 nm) with an intensity of 40 up to 200 mW cm^{-2} to induce photochemical bending. The strength of the light power is a trade-off between irradiated surface area and intensity and could be varied by the focus of the collimator. This procedure was performed in air (top row) and under water (bottom row). For the underwater measurement, a cuvette (5 cm x 5 cm x 5 cm) filled with water was used to submerge the film. In all other respects, the experimental set-up was the same as for the bending in air. For the analysis of the final bending angles, captures from the supplementary

Supplementary Video S1-2 were used, the respective time was from the tracked timeline in the video. The angles were measured with the program GIMP (**Table S4**).

Table S4: Measured Bending Angle after Irradiation

In Air	Angle Start	Angle End	Difference	Time
**1 - 525 nm**	68°	31°	37°	41s
**2 - 420 nm**	27°	64°	37°	38s
**3 - 525 nm**	64°	25°	39°	35s
**4 - 420 nm**	24°	64°	40°	30s
**In Water**				
**5 - 525 nm**	58°	27°	31°	48s
**6 - 420 nm**	30°	58°	28°	39s
**7 - 525 nm**	60°	28°	32°	55s

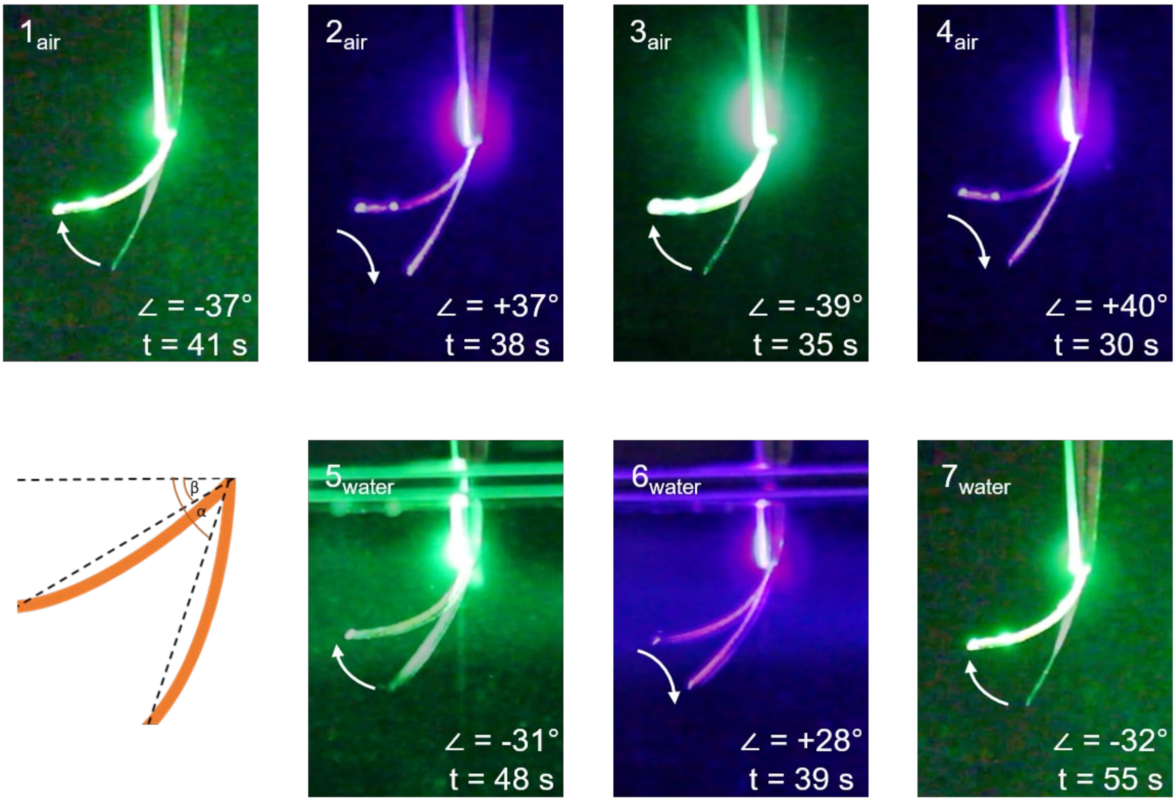

Figure S17. Bending of the polymer film $\mathbf{P_{Splay,60}}$ in water and air under illumination with violet (420 nm) and green (525 nm) light. Images captured from the **Supplementary Video S1-2**.

2.3.5 Bending of $P_{Splay,60}$ and Relaxation in the Dark.

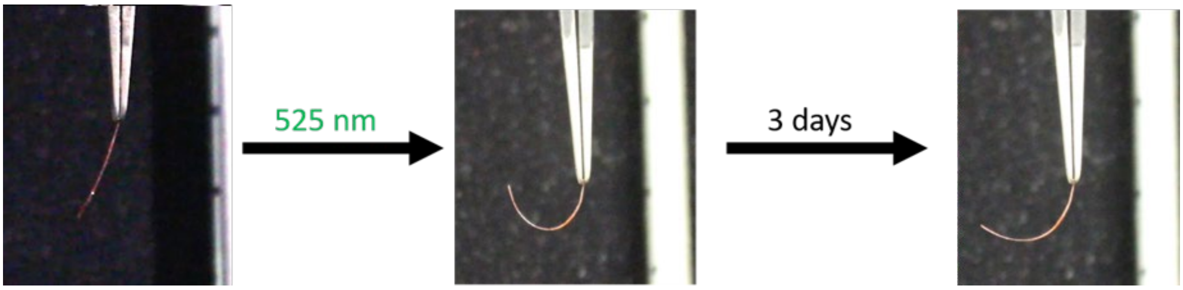

Figure S18. Bending of $\mathbf{P_{Splay,60}}$ (1.5 cm x 0.2 cm x 60 µm) before (left) and after (middle) irradiation with green light (525 nm) by light following the bending movement with the LED (tracking). Subsequently, the polymer was allowed to hang in darkness for 3 days to monitor the relaxation process (right). The marks on the metal rod to the right are spaced 1 cm apart.

2.3.6 UV-Vis Analysis: Relaxation of $\mathbf{P_{Planar,31}}$ after Irradiation with 525 nm.

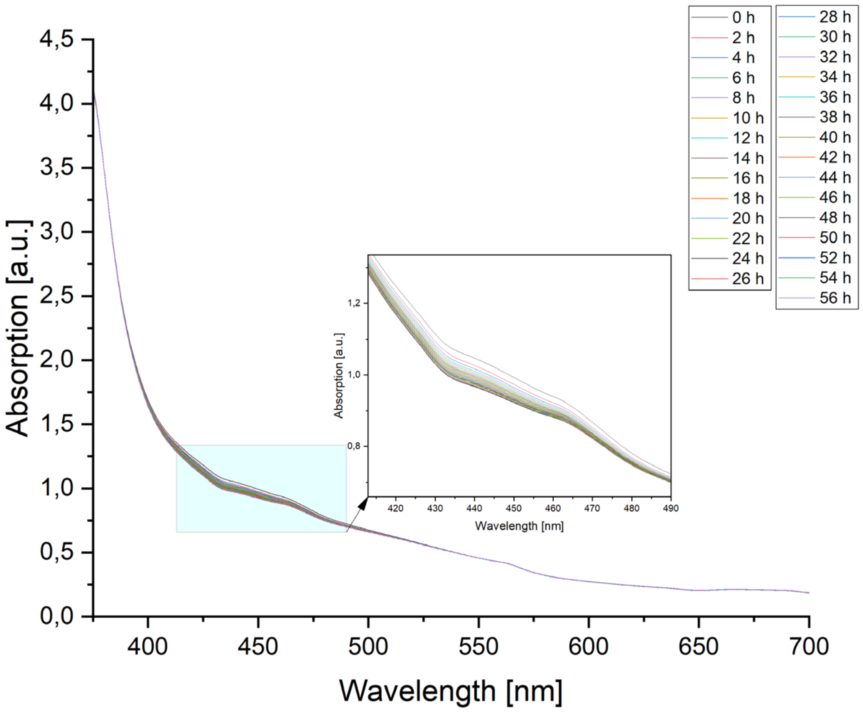

Figure S19. Half-life time measurement of $\mathbf{P_{Planar,31}}$ (2 cm x 1 cm x 39 µm) after irradiation with green light (525 nm) for 1 h. The half-life time for the thermal relaxation was determined at 23 °C under air every 2 h.

2.3.7 UV-Vis Analysis: Relaxation of $\mathbf{P}_{\mathbf{Planar,31}}$ after Irradiation with 525 nm Determined at 440 nm.

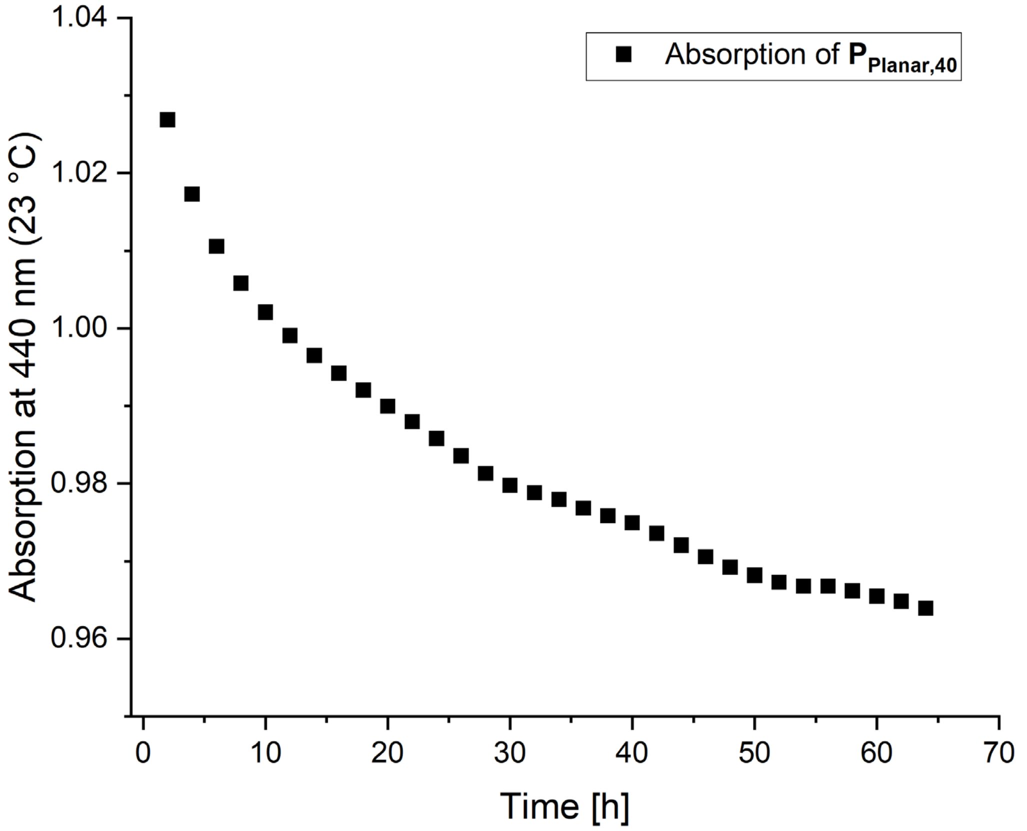

Figure S20. Measurement of the decay of the absorption signal at 440 nm of $\mathbf{P}_{\mathbf{Planar,31}}$ (2 cm x 1 cm x 40 µm) after irradiation with green light (525 nm) for 1 h at 23 °C. Because of the constraints of the azobenzene in the polymer, it is unlikely that the relaxation follows a first order kinetic. Therefore, the curve was not fitted to such a function.

2.3.8 IR-Analysis: Thermal Heating during Irradiation of $\mathbf{P_{Splay,40}}$ and $\mathbf{P_{Splay,60}}$.

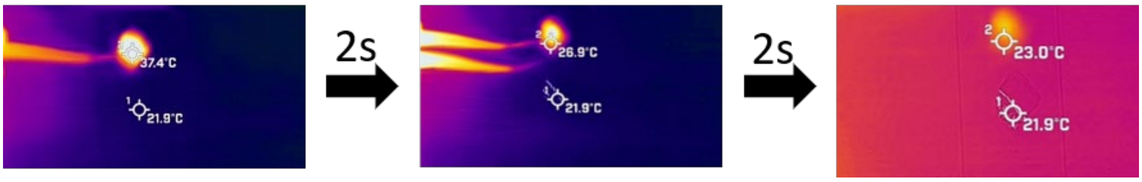

Figure S21. Thermal images taken with an infrared camera of two pieces of $\mathbf{P_{Splay,40}}$ (0.5 cm x 0.5 cm x 40 µm). The first measurement point (bottom) was a sample without irradiation and the second measurement point (top) after irradiation with green light (20 mW cm^{-2}) for 30s. The cooling down process is shown without irradiation in 2 second intervals.

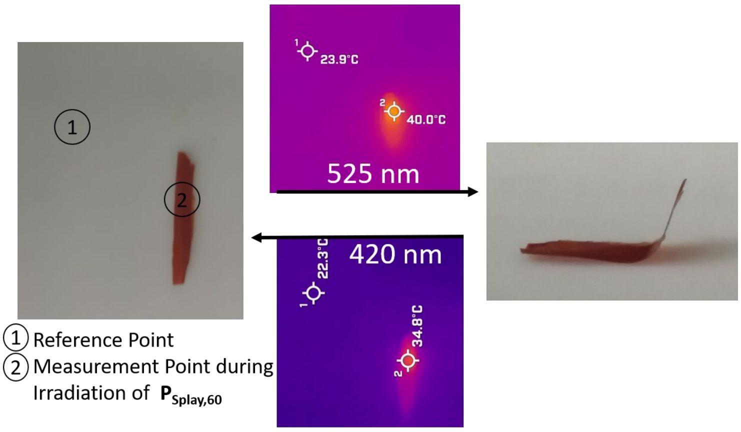

Figure S22. Thermal images taken with an infrared camera. The setup is shown on the left and the final bending angle on the right. An IR-video was taken during irradiation on $\mathbf{P_{Splay,60}}$ (2cm x 0.3 cm x 60 µm). The highest peak temperature recorded is shown in the picture with 40.0 °C for green light (525 nm) and 34.8 °C for violet irradiation (420 nm). The average temperature was around 33 °C for green light and 30 °C for violet light.

3. Preparation of the LCE

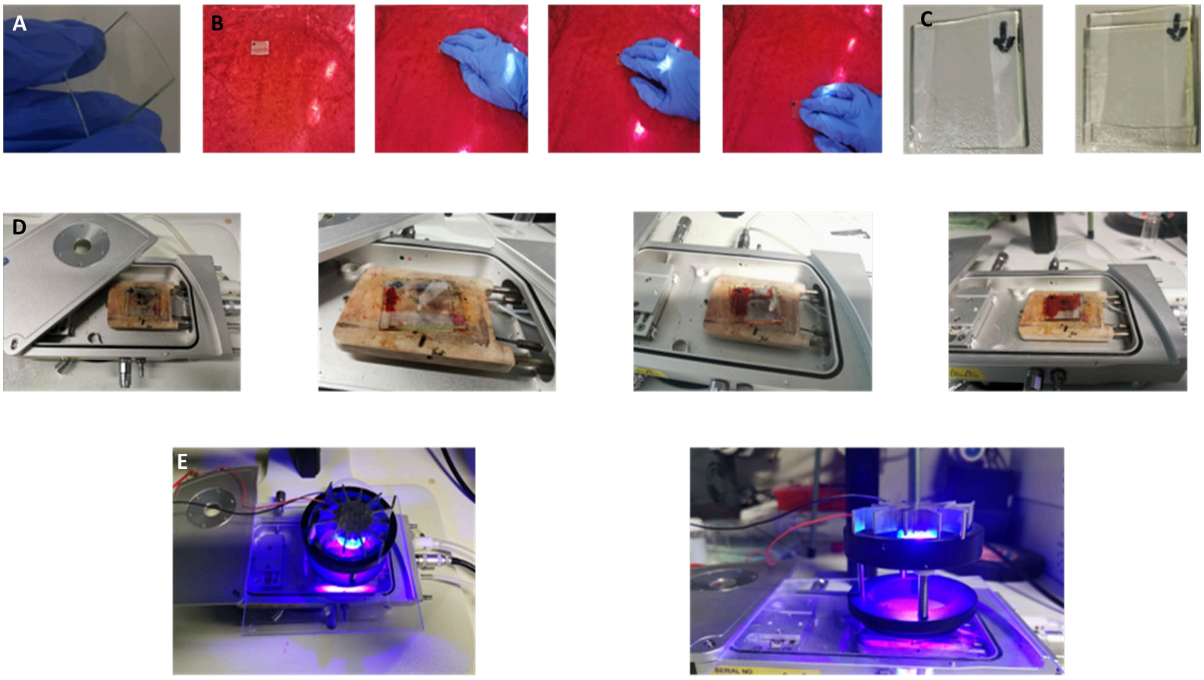

Figure S23. Visualization for the preparation of a photo bendable LCE within an LC cell. A) A glass substrate washed with hot water, EtOH, spin coated with an 5% wt PI solution and dried at 200 °C. B) Surface alignment by rubbing the glass substrate with gently pressure on a velvet cloth along one direction. C) A thin PTFE stripe of 10 µm, 30 µm ,or 50 µm thickness was cut out of a PTFE sheet and used as a spacer. A second PI coated non-rubbed glass substrate was used to form the LC cell. Both glass substrates were glued together. D) The LC cell was heated on the Linkam eating stage LTS 420 to 160°C. At this temperature the LC cell was filled with the LC mixture by capillary force. Afterwards, the temperature was slowly lowered (0.5 °C min^{-1}) to 130 °C. E)

Polymerization was initiated by employing a linearly polarized 420 nm light source from a 1 W LED, which was shone orthogonally to the planar alignment through a PMMA glass to activate the photocatalyst. This setup was maintained for a minimum of 5 hours. Afterwards, the temperature was raised to 155 °C again and hold for 15 min. The lamp was turned off and the temperature hold for another 15 min, before cooling down to room temperature and carefully opening the LC cell using a razor blade. To facilitate the extraction of the thin film, the razor blade and the LC cell were periodically immersed in hot water. The final thickness of the films was measured using a digital caliper.

3.1. Comparison of the Transparency Depending on the Alignment and Thickness.

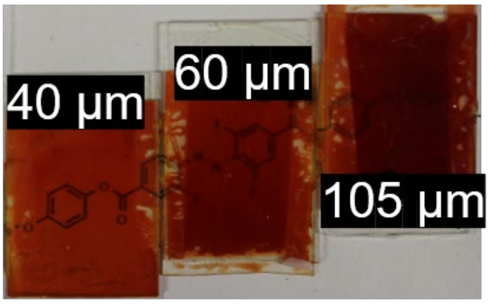

Figure S24. Photograph of the polymers (From left to right: $\mathbf{P}_{\mathbf{Splay,40}}$, $\mathbf{P}_{\mathbf{Splay,60}}$, $\mathbf{P}_{\mathbf{Splay,105}}$) still within the cell and their final thickness. It can be seen that this directly affects their transparency. The actual thickness of the films is higher than that of the spacer, because at the high polymerization temperature, the glue for the cell softens, widening the gap. The thicknesses of the films are uniform throughout.

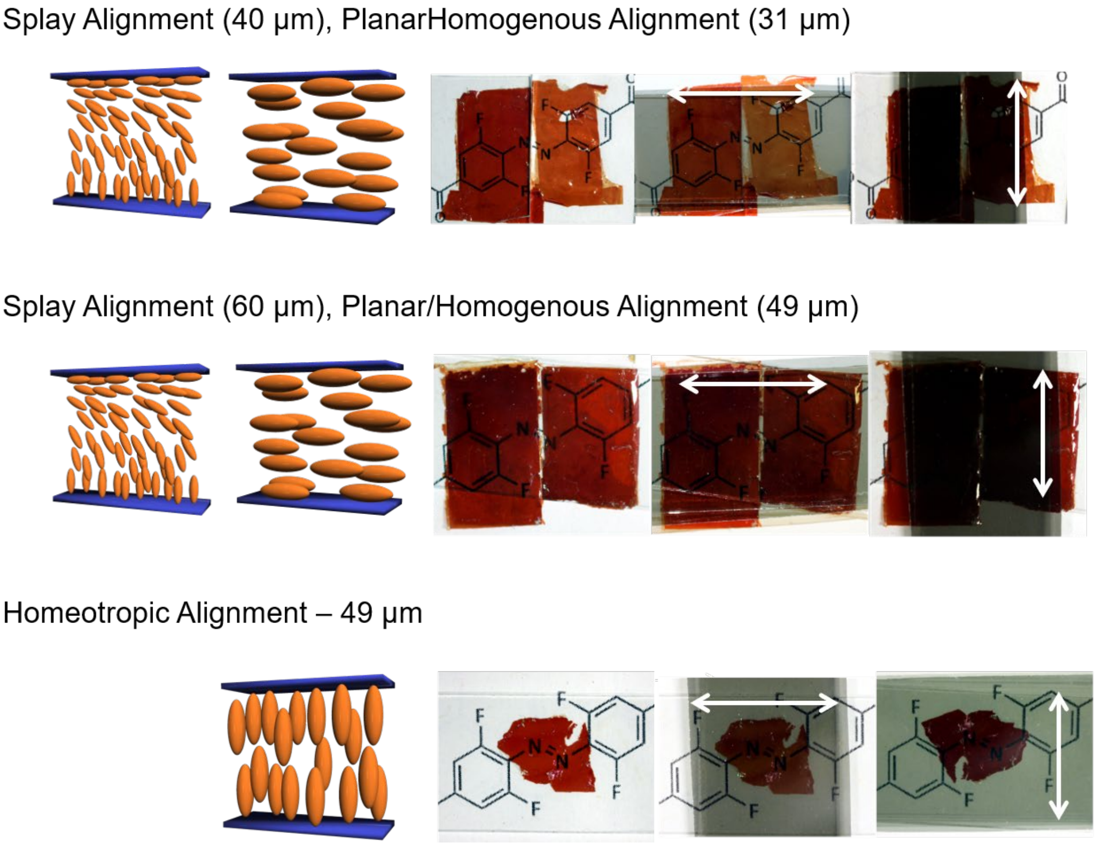

Figure S25. Test of the alignment by polarization filter. (Note: Azobenzene orientated along the polarization direction absorbs the light.) The splay and homogenous (planar) polymer side by side are photographed with no polarization filter (left), a polarizing filter orthogonal to the orientation of the mesogen (middle) and in plane with the mesogen (right) for the thickness of $\mathbf{P}_{\mathbf{Splay,40}}$ and $\mathbf{P}_{\mathbf{Planar,31}}$ (first row), $\mathbf{P}_{\mathbf{Splay,60}}$ and $\mathbf{P}_{\mathbf{Planar,49}}$ (middle row). The same procedure is shown in the last row for the homeotropic alignment of a $\mathbf{P}_{\mathbf{Homeotropic,49}}$ thick polymer film.

Scheme 1: Synthesis Overview

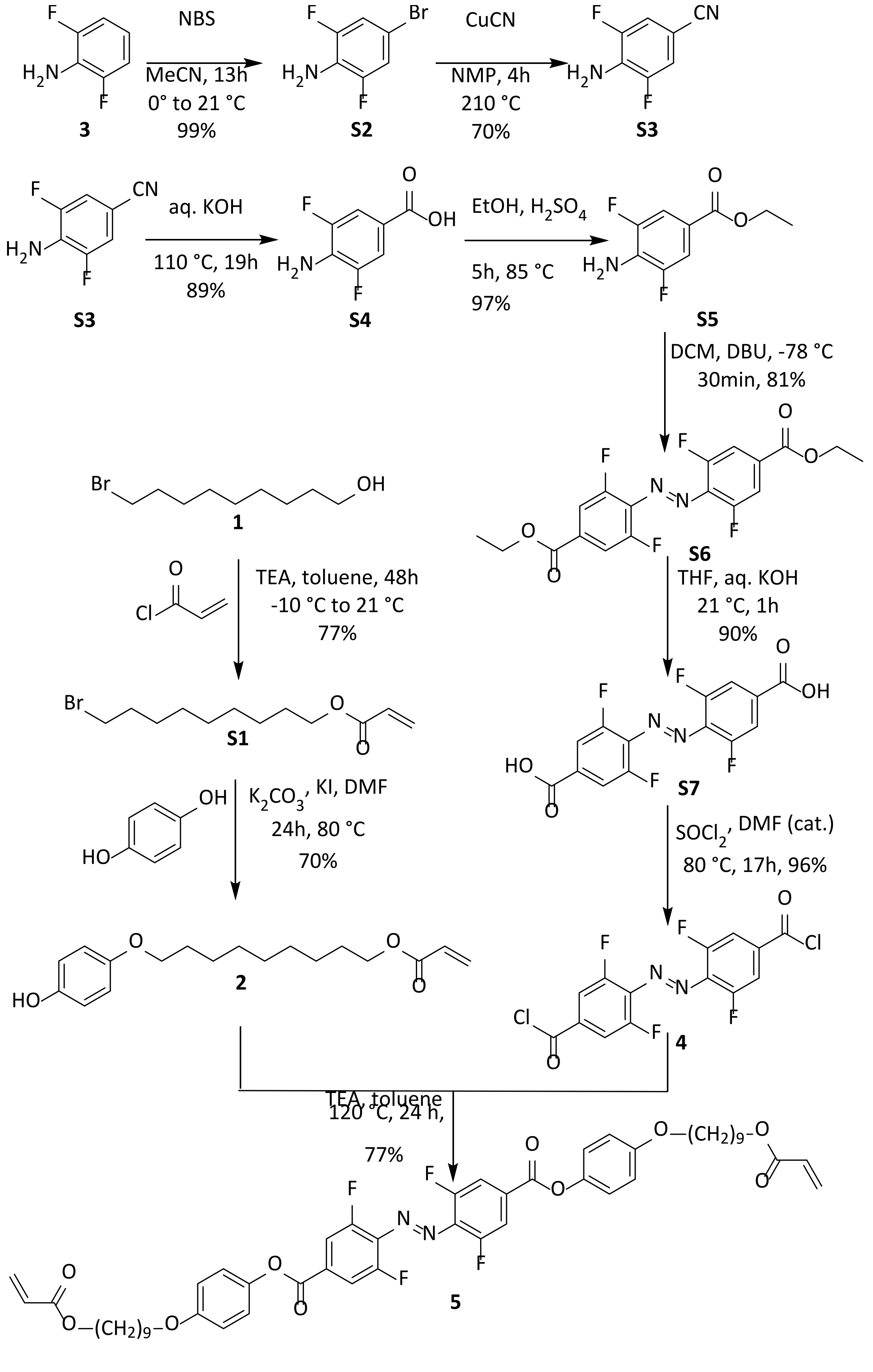

4. Syntheses

4.1 9-Bromononyl acrylate (**S1**)

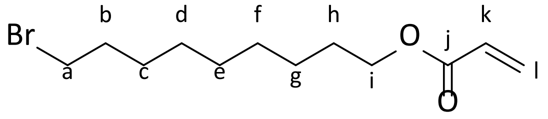

This reaction was performed under inert conditions.

Triethylamine (9.12 g, 90.2 mmol, 1.09 equiv.) and 9-bromo-nonanol (18.5 g, 82.8 mmol, 1.00 equiv.) were dissolved in dry toluene (90 mL). The mixture was then cooled to –10 °C *via* a salt and ice bath, and a solution of acryloyl chloride (8.16 g, 90.2 mmol, 1.09 equiv.) in dry toluene (30 mL) was added dropwise over the course of 30 min. The reaction was warmed to 21 °C and stirred for 48 h. The organic phase was subsequently washed with sat. solution of Na_2CO_3 (100 mL), an aqueous hydrochloric acid solution (1 M, 2 x 100 mL) and with brine (50 mL). The organic phase was then dried over $MgSO_4$, filtered and the solvent removed *in vacuo* to obtain a slightly yellow oil. After filtration through a short plug of silica (eluent: DCM) the product **S1** was obtained as a colorless oil. (17.7 g, 63.9 mmol, 77%)

^{1}H NMR (601 MHz, $CDCl_3$) δ 6.40 (dd, ^3J = 17.4, ^4J = 1.5 Hz, 1H, *H*-l), 6.12 (dd, ^3J = 17.4, ^3J = 10.5 Hz, 1H, *H*-k), 5.81 (dd, ^3J = 10.5, ^4J = 1.5 Hz, 1H, *H*-l), 4.15 (t, ^3J = 6.8 Hz, 2H, *H*-a), 3.41 (t, ^3J = 6.8 Hz, 2H, *H*-i), 1.85 (m, 2H, *H*-h), 1.69 – 1.64 (m, 2H, *H*-b), 1.31 (m, 10H, *H*-c,d,e,f,g).
^{13}C{^{1}H} NMR (151 MHz, $CDCl_3$) *δ* = 166.48 (*C*-l), 130.60 (*C*-j), 128.78, (*C*-k), 64.79 (*C*-a), 34.14 (*C*-i), 32.93 (*C*-h), 29.43 (*C*-b), 29.26 (*C*-c,d,e,f,g), 28.80 (*C*-c,d,e,f,g), 28.72 (*C*-c,d,e,f,g), 28.26 (*C*-c,d,e,f,g), 26.01 (*C*-c,d,e,f,g) ppm.
HRMS (ESI) *m/z* (%): $[M+Na]^+$ calcd for $[C_{12}H_{21}Br^{79}NaO_2]^+$ 299.06171; found 299.06185.
IR (ATR): $\tilde{\nu}$ = 2927 (m), 2854 (m), 1721 (s), 1636 (w), 1464 (w), 1406 (m), 1294 (w), 1269 (m), 1185 (s), 1085 (m), 983 (m), 965 (m), 809 (m), 722 (w) cm^{-1}.

4.2 9-(4-hydroxyphenoxy)nonyl acrylate **(2)**

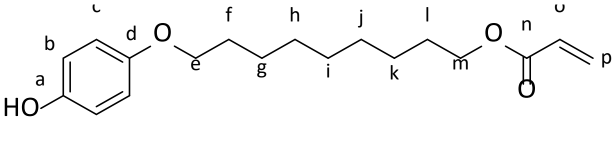

Hydroquinone (24.8 g, 225 mmol, 5.00 equiv.), **S1** (13.8 g, 45.0 mmol, 1.00 equiv.), K_2CO_3 (31.2 g, 225 mmol, 5.00 equiv.), a catalytic amount KI (100 mg) was dissolved in anhydrous DMF (150 mL). The solution was stirred for 24 h at 80 °C. Then, water (150 mL) was added and the mixture was placed in the fridge at 6 °C for 3 h. The precipitate was filtered and washed with water (3 x 100 mL). Afterwards, the filter cake was dissolved in ethyl acetate (200 mL). The organic layer was washed with water (3 x 150 mL), brine (3 x 150 mL) and dried over anhydrous $MgSO_4$. After filtration, the solvent was removed by evaporation under reduced pressure. The crude product was purified by silica gel column chromatography (ethyl acetate:hexane, 1:4) to obtain **2** as a pale-yellow solid (9.65 g, 31.5 mmol, 70%).

^{1}H NMR (600 MHz, $CDCl_3$) *δ* = 6.82 – 6.72 (m, 4H, *H*-b,c), 6.40 (dd, ^3J = 17.4, ^4J = 1.4 Hz, 1H, *H*-p), 6.12 (dd, ^3J = 17.4, ^3J = 10.4 Hz, 1H, *H*-o), 5.82 (dd, ^3J = 10.5, ^4J = 1.4 Hz, 1H, *H*-p), 4.54 (s, 1H, *H*O-a), 4.15 (t, ^3J = 6.7 Hz, 2H, *H*-e), 3.89 (t, ^3J = 6.6 Hz, 2H, *H*-m), 1.77 – 1.71 (m, 2H, *H*-l), 1.69 – 1.64 (m, 2H, *H*-f), 1.46 – 1.41 (m, 2H, *H*-k), 1.37 – 1.30 (m, 8H, *H*-g,h,I,j).

^{13}**C{^{1}H} NMR** (151 MHz,CDCl$_3$) δ = 166.58 (*C*-n), 153.45 (*C*-a), 149.49 (*C*-d), 130.67 (*C*-p), 128.76 (*C*-o), 116.12 (*C*-b,c), 115.74 (*C*-b,c), 68.79 (*C*-m), 64.87 (*C*-e), 29.54 (*C*-l), 29.48 (*C*-g,h,l,j), 29.42 (*C*-f), 29.29 (*C*-g,h,l,j) , 28.73 (*C*-g,h,l,j), 26.14 (*C*-g,h,l,j), 26.03 (*C*-k).
HRMS (EI, 70 eV) *m/z* (%): [M]$^+$ calcd for [$C_{18}H_{26}O_4$]$^+$ 306.18244; found 306.18256 (20), 110.1 (100).
IR (ATR): $\tilde{\nu}$ = 3372 (br), 2918 (m) 2851 (w), 1696 (m),1511 (s), 1473 (m), 1423 (m), 1392 (w), 1300 (w), 1256 (w), 1212 (s), 1024 (s), 983 (w), 827 (m), 809 (m), 766 (m), 751 (m) cm^{-1}.
R*f*: 0.5 (ethyl acetate:*n*-hexane, 1:4).

4.3 4-Bromo-2,6-difluoroaniline (**S2**)[5]

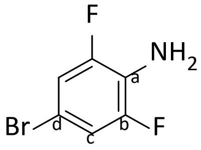

To a solution of 2,6-difluoroaniline (12.9 g, 100 mmol, 1.00 equiv.) in acetonitrile (200 mL), *N*-bromosuccinimide (17.8 g, 100 mmol, 1.00 equiv) dissolved in acetonitrile (100 mL) was added dropwise at 0°C over the duration of 1 h. The mixture was stirred for 12 h while warming up to 21°C, and then diluted with water (800 mL). The product was extracted with ethyl acetate (3 × 100 mL). The organic phase was dried over $MgSO_4$, filtered, and concentrated under reduced pressure. The crude residue was purified by column chromatography over silica gel (eluent: *n*-hexane:DCM, 4:1) to give **S2** as a colorless solid (17.2 g, 99%, Lit.: 99%).[5]

^{1}**H NMR** (600 MHz, CDCl$_3$) δ 6.99 (d, ^3J = 7.8 Hz, 2H, *H*-c), 3.73 (s, 2H, N*H*$_2$-a) ppm.
^{13}**C{^{1}H} NMR** (151 MHz, CDCl$_3$) δ 151.88 (dd, ^1J = 244.1 Hz, ^4J = 8.6 Hz, *C*-b), 123.64 (t, ^2J = 16.2 Hz, *C*-a), 114.85 (dd, ^2J = 18.5, ^4J = 7.2 Hz, *C*-c), 107.20 (t, ^3J = 11.6 Hz, *C*-d) ppm.
^{19}**F NMR** (565 MHz, CDCl$_3$) δ -130.69 (d, ^3J = 6.3 Hz, *F*-b) ppm.
IR (ATR): $\tilde{\nu}$ = 3421(m), 3328 (m), 1643 (w), 1605 (m), 1583 (m), 1497 (s), 1428 (s), 1299 (m), 1272 (m), 1199 (w), 1150 (s), 963 (s), 869 (m), 839 (m), 761 (w), 717 (s) $^{-1}$.
HRMS (EI, 70 eV) *m/z* (%): calcd for [$C_6H_4NBr^{79}F_2$]$^+$ 206.94921, found 206.94897(100).
Mp: 65 °C.
R$_f$: 0.40 (*n*-hexane:DCM, 1:2).

4.4 4-Amino-3,5-difluorobenzonitrile (**S3**) [6]

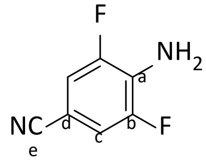

A mixture of copper(I) cyanide (29.3 g, 325 mmol, 1.70 equiv.) and 4-bromo-2,6-difluoroaniline (**S2**) (39.5 g, 190 mmol, 1.00 equiv.) in *N*-methyl-2-pyrrolidone (120 mL) was heated to 210 °C under anhydrous conditions for 4 h. After cooling to 30 °C the resulting mixture was poured into aqueous ammonia (conc. 30%) until a white precipitate was formed. The suspension was extracted with toluene (5 × 100 mL). The organic phase was washed with water (3 x 100mL), brine (2 x 100 mL), dried over $MgSO_4$ and filtered. The solvent was removed *in vacuo*. The crude product was dissolved in dichloromethane and purified by adsorption filtration through a large column of silica (eluent: DCM:hexane, 1:1). **S3** was obtained as a colorless solid (20.5 g, 133 mmol, 70%, Lit.: 87%).[7]

^{1}**H NMR** (600 MHz, CDCl$_3$) δ =7.14 (dd, ^3J = 6.1, ^5J = 2.2 Hz, 2H, *H*-c), 4.27 (s, 2H, N*H*$_2$-a) ppm.
^{13}**C{^{1}H} NMR** (151 MHz, CDCl$_3$) δ 150.66 (dd, ^1J = 243.5 Hz, ^4J = 9.1 Hz, *C*-b), 129.75 (t, ^2J = 15.7 Hz, *C*-a), 118.05 (t, ^5J = 3.4 Hz, *C*-e), 115.60 (dd, ^2J = 17.5, ^4J = 7.1 Hz, *C*-c), 98.47 (t, ^3J = 11.1 Hz, *C*-d) ppm.

^{19}**F NMR** (565 MHz, $CDCl_3$) δ = -130.76 (dd, ^3J = 6.1 Hz, ^5J = 2.2 Hz, *F*-b) ppm.
IR (ATR): $\tilde{\nu}$ = 3376 (w), 3313 (w), 2224 (m), 1650 (w), 1608 (w), 1587 (m), 1523 (m), 1443 (s), 1338 (m), 1163 (s), 977 (s), 875 (s), 862 (m), 727 (s) cm^{-1}.
HRMS (EI, 70 eV) *m/z* (%): $[M]^+$ calcd for $[C_7H_4N_2F_2]^+$ 154.03371; found 154.03383 (100).
Mp: 112 °C.
R$_f$: 0.40 (*n*-hexane:DCM, 1:2).

4.5 4-Amino-3,5-difluorobenzoic acid (**S4**)[6]

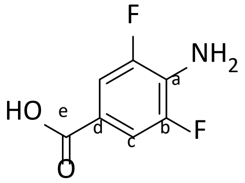

A mixture of 4-amino-3,5-difluorobenzonitrile (**S3**) (5.00 g, 32,4 mmol, 1.00 equiv.) was dissolved in aq. KOH (25 wt%, 100 mL). The mixture was heated to 110 °C for 19 h. After cooling down to 21 °C, the aqueous phase was acidified to pH = 2 with HCl (2 M) and extracted with ethyl acetate (2 × 100 mL). The combined organic extracts were washed with water (3 x 75 mL), brine (2 x 75 mL), dried over $MgSO_4$ and filtered. The solvent was removed *in vacuo* to obtain **S4** as a slightly yellowish solid (4.99 g, 28.8 mmol, 89%, Lit.: 89%).[8]

^{1}**H NMR** (600 MHz, DMSO-*d$_6$*) δ = δ 7.39 (dd, ^3J = 7.2 Hz, ^5J = 2.4 Hz, 2H, *H*-c), 6.04 (s, 2H, NH_2-a) ppm.
^{13}**C{^{1}H} NMR** (151 MHz, DMSO-*d$_6$*) δ = 166.12 (*C*-e), 149.80 (dd, ^1J = 239.6, ^4J = 9.5 Hz, *C*-b), 130.61 (t, ^2J = 16.7 Hz, *C*-a), 112.31 (dd, ^2J = 15.8 Hz, ^4J = 5.9 Hz, *C*-c), 59.83 (*C*-d) ppm.
^{19}**F NMR** (565 MHz, DMSO-*d$_6$*) δ -131.62 (d, ^3J = 7.2 Hz, *F*-b) ppm.
IR (ATR): $\tilde{\nu}$ = 3397 (w), 1683 (m), 1628 (s), 1587 (m), 1538 (w), 1454 (m), 1422 (s), 1341 (s), 1277 (s), 1243 (m), 1145 (m), 1082 (w), 953 (m), 889 (m), 765 (s), 719 (s) cm^{-1}.
HRMS (EI, 70 eV) *m/z* (%): $[M]^+$ calcd for $[C_7H_5NO_2F_2]^+$ 173.02829; found 173.02830 (75), 156.1 (100).
Mp: 171 °C.
R$_f$: 0.90 (ethyl acetate).

4.6 Ethyl 4-amino-3,5-difluorobenzoate (**S5**)[6]

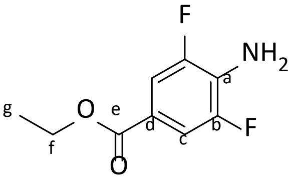

S4 (2.42 g, 14.0 mmol) was dissolved in EtOH (50 mL) and H_2SO_4 (95%, 1 mL), and heated to 85 °C for 5 h. The mixture was neutralized (pH 7) with saturated $NaHCO_3$, and extracted with DCM (3 × 50 mL). The organic phase was dried over $MgSO_4$, filtered, and concentrated under reduced pressure to give **S5** as a pale brown solid (2.74 g, 13.6 mmol, 97%, Lit.: 97%.)[8]

^{1}**H NMR** (600 MHz, $CDCl_3$) δ = 7.53 (dd, ^3J = 7.1, ^5J = 2.2 Hz, 2H, *H*-c), 4.33 (q, ^3J = 7.1 Hz, 2H, *H*-f), 4.13 (s, 2H, NH_2-a), 1.36 (t, ^3J = 7.1 Hz, 3H, *H*-g) ppm.
^{13}**C{^{1}H} NMR** (151 MHz, $CDCl_3$) δ = 165.30 (t, ^5J = 3.5 Hz, *C*-e), 150.80 (dd, ^1J = 240.8 Hz, ^4J = 7.9 Hz, *C*-b), 128.92 (t, ^3J = 16.1 Hz, *C*-a), 118.72 (t, ^4J = 8.2 Hz, *C*-d), 113.13 – 112.08 (m, *C*-c), 61.20 (*C*-f), 14.45 (*C*-g) ppm.
^{19}**F NMR** (565 MHz, $CDCl_3$) δ -132.93 (dd, ^3J = 7.2 Hz, ^5J = 2.3 Hz, *F*-b) ppm.
IR (ATR): $\tilde{\nu}$ = 3505 (w), 3368 (m), 1697 (s), 1629 (s), 1585 (m), 1531 (m), 1477 (w), 1444 (m), 1397 (m), 1370 (m), 1339 (s), 1270 (s), 1222 (s), 1133 (m), 1089 (s), 1027 (s), 946 (s), 881 (m), 760 (m) cm^{-1}.
HRMS (EI, 70 eV) *m/z* (%): $[M]^+$ calcd for $[C_9H_9NO_2F_2]^+$ 201.0599; found 201.05932 (15), 156.1 (100).

R$_f$: 0.30 (*n*-hexane:DCM, 1:1).

4.7 (*E*)-4,4'-(Diazene-1,2-diyl)bis(3,5-difluorobenzoic acid) (**S6**)[9]

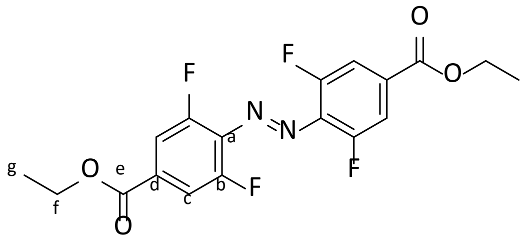

To a solution of **S5** (637 mg, 2.50 mmol, 1.00 equiv.) dissolved in DCM (40 mL) 1,8-diazabicyclo[5.4.0]undec-7-ene (761 mg, 5.00 mmol, 2.00 equiv.) was added. The solution was stirred at 21 °C for 5 min before being cooled down to −78 °C. *N*-Chlorosuccinimide (668 mg, 5.00 mmol, 2.00 equiv.) was added as a solid to the reaction mixture. The orange solution was stirred for 30 min at −78 °C, before quenching by addition of a saturated bicarbonate solution (50 mL). The organic layer was separated, washed sequentially with water (3 x 50 mL) and HCl (1 M, 50 mL), dried over $MgSO_4$, filtered and concentrated *in vacuo*. The residue was purified by silica gel flash chromatography (eluent: gradient *n*-hexane:DCM, 1:0 → 1:1) to obtain **S6** as a red solid (402 mg, 1.01 mmol, 81%, Lit.: 81%)[8].

^{1}H NMR (600 MHz, $CDCl_3$) δ = 7.75 (d, ^3J = 9.1 Hz, 4H, *H*-c), 4.43 (q, ^3J = 7.1 Hz, 4H, *H*-f), 1.43 (t, ^3J = 7.1 Hz, 6H, *H*-g) ppm.
^{13}C{^{1}H} NMR (151 MHz, $CDCl_3$) δ = 163.84 (*C*-e), 155.17 (dd, ^1J = 262.6 Hz, ^4J = 3.8 Hz, *C*-b) ,134.31 (t, ^3J = 10.1 Hz, *C*-a), 133.89 (t, ^4J = 9.2 Hz, *C*-d), 114.07 (dd, ^2J = 21.7, ^4J = 3.9 Hz, *C*-c), 62.34 (*C*-f), 14.36 (*C*-g) ppm.
^{19}F NMR (565 MHz, $CDCl_3$) δ = -119.5 (d, ^3J = 9.1 Hz, *F*-b) ppm.
IR (ATR): $\tilde{\nu}$ = 1721 (m), 1573 (m), 1434 (m), 1369 (s), 1330 (m), 1238 (m), 1192 (m), 1089 (w), 1052 (s), 1017 (s), 886 (s), 767 (s), 752 (s) cm^{-1}.
HRMS (EI, 70 eV) *m/z* (%): $[M]^+$ calcd for $[C_{18}H_{14}N_2O_4F_4]^+$ 398.08842; found 398.08876 (15), 101.1 (100).
Mp: 147°C.
R*f*: 0.35 (*n*-hexane:DCM, 1:1).

4.8 (*E*)-4,4'-(Diazene-1,2-diyl)bis(3,5-difluorobenzoic acid) (**S7**)[6]

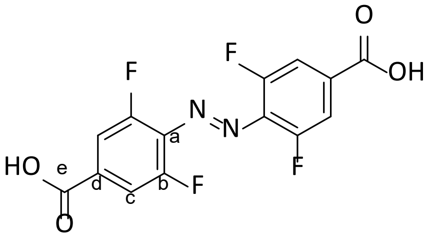

Diethyl 4,4'-(diazene-1,2-diyl)-bis(3,5-difluorobenzoate) (**S6**) (1.19 g, 3.00 mmol, 1.00 equiv.) was dissolved in THF (40 mL). KOH (673 mg, 12.0 mmol, 4.00 equiv.) dissolved in H_2O (40 mL) was added. The reaction mixture was stirred for 1 h and acidified with HCl (1 M) until a precipitate was formed. The precipitate was filtered and dried in a vacuum oven at 50°C for 24 h to yield the desired product **S7** (924 mg, 2.70 mmol, 90 %, Lit.: 94%) as a pink solid. [6]

^{1}H NMR (600 MHz, DMSO-d_6) δ = 7.80 (d, ^3J = 9.5 Hz, 4H, *H*-c) ppm.
^{13}C{^{1}H} NMR (151 MHz, DMSO-d_6) δ = 202.28 (*C*-e), 192.00 (dd, ^{1}J = 260.9, ^{4}J =3.7 Hz, *C*-b), 173.11 (t, ^4J = 9.0 Hz, *C*-d), 170.65 (t, ^3J = 10.4 Hz, *C*-a), 151.61 (dd, ^2J = 21.3, ^4J = 3.7 Hz, *C*-c) ppm.
^{19}F NMR (565 MHz, DMSO-d_6) δ = -120.06 (d, ^3J = 9.5 Hz, *F*-b) ppm.
HRMS (ESI) *m/z* (%): $[M-H]^-$ calcd for $[C_{14}H_5N_2O_4F_4]^-$ 341.01909; found 341.01902.
IR (ATR): $\tilde{\nu}$ = 2871 (br), 1693 (s), 1577 (s), 1481 (m), 1436 (m), 1414 (m), 1259 (s), 1195 (m), 1054 (s), 893 (s), 879 (m), 773 (s), 729 (m) cm^{-1}.
Mp: >300 °C.

4.9 (*E*)-4,4'-(Diazene-1,2-diyl)bis(3,5-difluorobenzoyl chloride) (4)

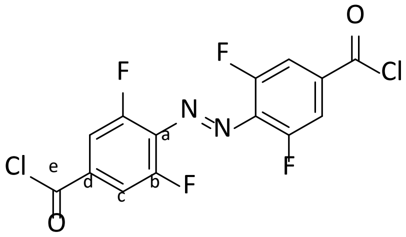

This reaction was performed under inert conditions.

In an oven dried flask, **S7** (1.03 g, 3.00 mmol, 1.00 equiv.) was dissolved in an excess of $SOCl_2$ (5 mL). Then, a single drop of dry DMF was added and the reaction mixture heated up to 80 °C for 17 h. Afterwards, the solvent was removed *in vacuo* by adding a cooling trap to the apparatus. The crude product was transferred to the glovebox and crystallized in dry toluene at -20 °C. After filtration, the dark red solid **4** was obtained in 96% (1.09 g, 2.88 mmol) yield.

^{1}H NMR (600 MHz, $CDCl_3$) δ = 7.86 (d, ^3J = 8.4 Hz, 4H, *H*-c) ppm.
^{13}C{^{1}H} NMR (151 MHz, $CDCl_3$) δ = 165.92 (*C*-e), 155.06 (dd, ^2J = 265.0, ^4J = 3.8 Hz, *C*-b), 136.05 (t, ^4J = 9.1 Hz, *C*-d), 135.54 (t, ^3J = 10.4 Hz, *C*-a), 115.68 (dd, ^2J = 22.1, ^4J = 4.4 Hz, *C*-c).
^{19}F NMR (565 MHz, $CDCl_3$) δ = -117.46 (d, ^3J = 8.4 Hz, *F*-b) ppm.
HRMS (EI, 70 eV) *m/z* (%): $[M]^+$ calcd for $[C_{14}H_4N_2O_4F_4]^+$ 377.95801; found 377.95805 (10), 86.1 (100).
IR (ATR): $\tilde{\nu}$ = 1746 (m), 1698 (m), 1684 (w), 1576 (s), 1558 (m), 1540 (w), 1473 (w), 1431 (m), 1418 (m), 1396 (w), 1328 (w), 1260 (w), 1123 (m), 1054 (s), 1004 (s), 891 (m), 878 (w), 807 (m), 772 (w), 701 (m) cm^{-1}.

4.10 (*E*)-4,4'-(Diazene-1,2-diyl)bis(3,5-difluorobenzoic acid) (**5**)

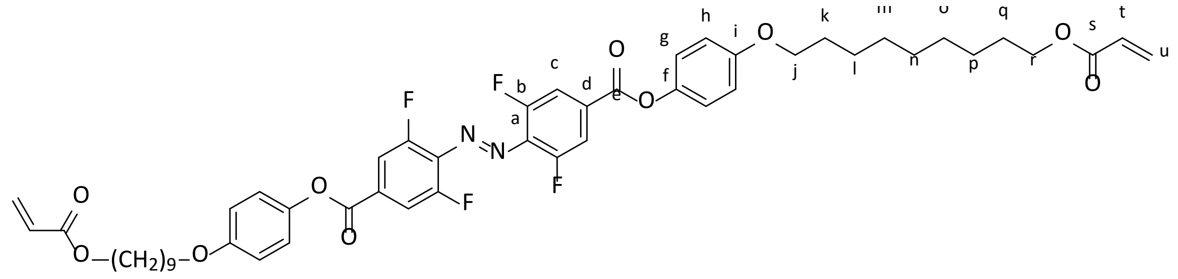

This reaction was performed under inert conditions.

In a glovebox, **4** (342 mg, 1 mmol, 1 equiv.) and **2** (674 mg, 2.20 mmol, 2.20 equiv.) and triethylamine (1 mL) were dissolved in toluene (30 mL). The reaction was heated to 110 °C for 24 h. Then, the solvent was removed *in vacuo* and the crude product was directly purified by column chromatography (eluent gradient: Hexane -> DCM) to afford **5** as an orange solid. (707 mg, 0.769 mmol, 77%)

^{1}H NMR (600 MHz,$CDCl_3$) δ = 7.91 (d, ^3J = 9.3 Hz, 4H, *H*-c), 7.14 (d, ^3J = 9.0 Hz, 4H, *H*-g), 6.95 (d, ^3J = 9.0 Hz, 4H, *H*-h), 6.40 (dd, ^2J = 17.4, ^4J = 1.5 Hz, 2H, *H*-u), 6.13 (dd, ^3J = 17.4, ^3J = 10.4 Hz, 2H, *H*-t), 5.82 (dd, ^3J = 10.4, ^4J = 1.5 Hz, 2H, *H*-u), 4.16 (t, ^3J = 6.7 Hz, 4H, *H*-j), 3.97 (t, ^3J = 6.5 Hz, 4H, *H*-r), 1.83 - 1.77 (m, 4H, *H*-q), 1.71 – 1.65 (m, 4H, *H*-k), 1.48-1.46 (m, 4H, *H*-p), 1.37 (s, 16H, *H*-l,m,n,o).
^{13}C{^{1}H} NMR (151 MHz,$CDCl_3$) δ = 166.51 (*C*-s), 162.84 (*C*-e), 157.43 (*C*-f), 155.24 (d, ^1J = 263.4 Hz, *C*-b), 143.88 (*C*-i), 134.71 (t, ^2J = 10.0 Hz, *C*-a), 133.13 (t, ^3J = 9.5 Hz, *C*-d), 130.61 (*C*-u), 128.80 (*C*-t), 122.21 (*C*-g), 115.36 (*C*-h), 114.65 (dd, ^2J = 21.7, ^4J = 4.0 Hz, *C*-c), 68.56 (*C*-r), 64.83 (*C*-j), 29.57 (*C*-q), 29.43 (*C*-l,m,n,o), 29.37 (*C*-k), 29.32 (*C*-l,m,n,o), 28.75 (*C*-l,m,n,o), 26.16 (*C*-l,m,n,o), 26.05 (*C*-p).
^{19}F NMR (565 MHz, $CDCl_3$) δ = -118.92 (d, ^3J = 9.3 Hz, *F*-b).
HRMS (ESI) *m/z* (%): $[M+Na]^+$ calcd for $[C_{50}H_{54}F_4N_2NaO_{10}]^+$ 941.36068; found 941.36095.
IR (ATR): $\tilde{\nu}$ = 2923 (w), 1710 (m), 1575 (w), 1501 (w), 1473 (m), 1434 (w), 1336 (m), 1295 (m), 1204 (s), 1184 (s), 1110 (m), 1048 (m), 1017 (m), 988 (m), 968 (m), 958 (m), 912 (w), 898 (w), 827 (m), 812 (s), 773 (m), 886 (m), 752 (m), 725 (w) cm^{-1}.
Mp: Cr120N153I
R*f*: 0.5 (*n*-hexane:DCM, 1:1).

4.11 Bis(9-(acryloyloxy)nonyl) 4,4'-(diazene-1,2-diyl)(*E*)-bis(3,5-difluorobenzoate) (**S8**)

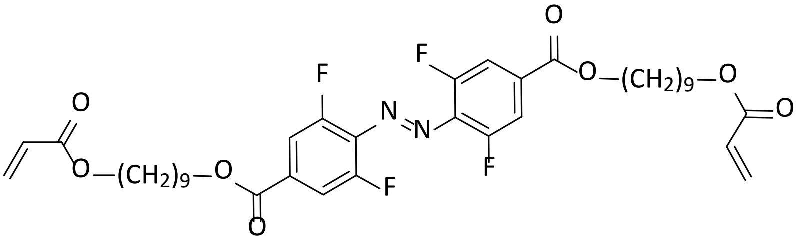

In a glovebox, **6** (342 mg, 1 mmol, 1 equiv.) and excess of nonane-1,9-diol (641 mg, 4.00 mmol, 4.00 equiv.) and triethylamine (1 mL) were dissolved in toluene (30 mL). The reaction was heated to 110 °C for 24h. Then, the solvent was removed in vacuo and the crude product was purified by column chromatography (cyclohexane: ethyl acetate, 2:1) to afford a crude product (532 mg), which was used without further purification.

Triethylamine (202 mg, 2.00 mmol, 2.00 equiv.) and the crude material (532 mg, 0.840 equiv.) were dissolved in dry THF (10 mL). The mixture was then cooled to –10 °C *via* a salt and ice bath, and a solution of acryloyl chloride (270 mg, 3.00 mmol, 3.00 equiv.) in dry THF (5 mL) was added dropwise. The reaction was warmed to 21°C and stirred for 18 h. DCM (25 mL) was added to the organic phase. The organic phase is subsequently washed with sat. solution of sodium carbonate (2 x 20 mL), an aqueous hydrochloric acid solution (1 M, 2 x 20 mL) and with brine (20 mL). The organic phase was then dried over $MgSO_4$, filtered and the solvent removed *in vacuo*. The crude product was purified by column chromatography (eluent: DCM) and crystalized in acetonitrile to obtained **S8** as an orange solid. (360 mg, 490 µmol, 49%)

^{1}H NMR (600 MHz, $CDCl_3$) δ = 7.74 (d, *J* = 9.0 Hz, 4H), 6.40 (dd, *J* = 17.3, 1.5 Hz, 2H), 6.12 (dd, *J* = 17.4, 10.4 Hz, 2H), 5.81 (dd, *J* = 10.4, 1.5 Hz, 2H), 4.36 (t, *J* = 6.7 Hz, 4H), 4.15 (t, *J* = 6.7 Hz, 4H), 1.79 (dt, *J* = 14.6, 6.8 Hz, 4H), 1.71 – 1.64 (m, 4H), 1.47 – 1.33 (m, 21H).
^{13}C{^{1}H} NMR (151 MHz, $CDCl_3$) δ = 166.50, 156.04 (d, *J* = 3.1 Hz), 154.30 (d, *J* = 3.6 Hz), 130.62, 128.78, 114.13 (d, *J* = 2.8 Hz), 113.98 (d, *J* = 3.5 Hz), 66.45, 64.80, 29.51, 2x 29.31, 28.74, 28.71, 26.08, 26.04.
^{19}F NMR (565 MHz, $CDCl_3$) δ = -117.97 (d, *J* = 6.6 Hz, (*E*-isomer <5%), *F*-b), -119.45 (d, *J* = 9.0 Hz (*Z*-isomer), *F*-b).
HRMS (ESI) *m/z* (%): $[M+Na]^+$ calcd for $[C_{38}H_{46}N_2O_8F_4Na]^+$ 757.30825; found 757.30683.
IR (ATR): $\tilde{\nu}$ = 2928 (m), 2855 (m), 1716 (s), 1577 (m), 1474 (w), 1431 (m), 1336 (m), 1232 (s), 1192 (s), 1088 (w), 1049 (s), 1010 (m), 982 (m), 883 (m), 810 (w), 768 (m), 753 (m).
Mp: 53 °C

4.12 Bis(4-hexylphenyl) 4,4'-(diazene-1,2-diyl)(E)-bis(3,5-difluorobenzoate) (**6**)

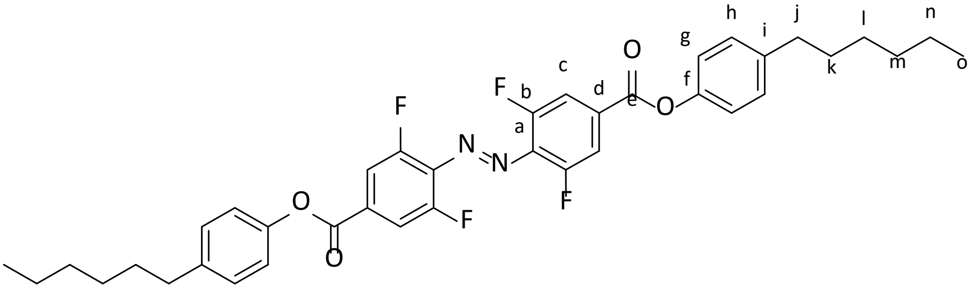

This reaction was performed under inert conditions.

In a glovebox, **4** (342 mg, 1 mmol, 1 equiv.) and hexylphenol (392 mg, 2.20 mmol, 2.20 equiv.) and triethylamine (1 mL) were dissolved in toluene (30 mL). The reaction was heated to 110 °C for 24 h.

Then, the solvent was removed *in vacuo* and the crude product was directly purified by column chromatography (eluent gradient: Hexane -> DCM) to afford **6** as an orange solid. (541 mg, 0.800 mmol, 80%)

^{1}H NMR (600 MHz, $CDCl_3$) δ = 7.93 (d, J = 8.5 Hz, 4H, *H*-c), 7.27-7.26 (m, 4H, *H*-g), 7.15 (d, J = 8.5 Hz, 4H, *H*-h), 2.68 – 2.63 (m, 4H, *H*-j), 1.65 (p, J = 7.7, 7.3 Hz, 4H, *H*-k), 1.38-1.31 (m, 12H, *H*-l,m,n), 0.94-0.91 (m, 6H, *H*-o) ppm.
^{13}C{^{1}H} NMR (151 MHz, $CDCl_3$) δ = 162.64 (*C*-e), 155.24 (dd, ^1J = 263.2, ^4J = 3.7 Hz, (*C*-b)), 148.48 (*C*-f), 141.40 (*C*-i), 134.59 (t, ^2J = 10.2 Hz, (*C*-a)), 133.02 (t, ^4J = 9.4 Hz, (*C*-d)), 129.66 (*C*-g), 121.10 (*C*-h), 114.66 (dd, ^2J = 21.8, ^4J = 3.9 Hz, (*C*-d)), 35.54 (*C*-j), 31.85 (*C*-l,m,n), 31.56 (*C*-k), 29.10 (*C*-l,m,n), 22.76 (*C*-l,m,n), 14.25 (*C*-o) ppm.
^{19}F NMR (565 MHz, $CDCl_3$) δ = -118.90 (d, ^3J = 9.3 Hz, *F*-b) ppm.
HRMS (EI) *m/z* (%): $[M+]^+$ calcd for $[C_{38}H_{38}F_4N_2O_4]^+$ 662.27564; found 662.27622.
IR (ATR): $\tilde{\nu}$ = 2953 (w), 2930 (w), 2854 (w), 1727 (s), 1580 (m), 1510 (m), 1467 (w), 1429 (s), 1338 (s), 1216 (s), 1187 (s), 1096 (w), 1037 (s), 1020 (m), 971 (w), 954 (m), 876 (s), 852 (m), 803 (m), 783 (m), 724 (w) cm^{-1}.
Mp: Cr91N247I
R*f*: 0.6 (*n*-hexane:DCM, 1:1).

5. References

[1] P. A.Heiney, http://www.datasqueezesoftware.com, 2006.
[2] P. Scherrer, *Nachr. Ges. Wiss. Goettingen Math.-Phys. Kl.* **1918**, 2, 98.
[3] O. V. Dolomanov, Bourhis, L. J., Gildea, R. J., Howard, J. A. K., Puschmann, H., *J. Appl. Crystallogr.* **2009**, 42.
[4] *The ACS Style Guide: Effective Communication of Scientific Information*, American Chemical Society, Washington, DC **2006**.
[5] J. Moreno, M. Gerecke, L. Grubert, S. A. Kovalenko, S. Hecht, *Angew. Chem. Int. Ed.* **2016**, 55, 1544.
[6] B. Heinrich, K. Bouazoune, M. Wojcik, U. Bakowsky, O. Vázquez, *Org. Biomol. Chem.* **2019**, 17, 1827.
[7] M. Cigl, A. Bubnov, M. Kašpar, F. Hampl, V. Hamplová, O. Pacherová, J. Svoboda, *J. Mater. Chem. C* **2016**, 4, 5326.
[8] S. Schultzke, M. Walther, A. Staubitz, *Molecules* **2021**, 26, 3916.
[9] A. Antoine John, Q. Lin, *J. Org. Chem.* **2017**, 82, 9873.

6. ^{1}H, ^{13}C{^{1}H} and ^{19}F NMR Spectra of the Purified Compounds

9-Bromononyl acrylate (**S1**)

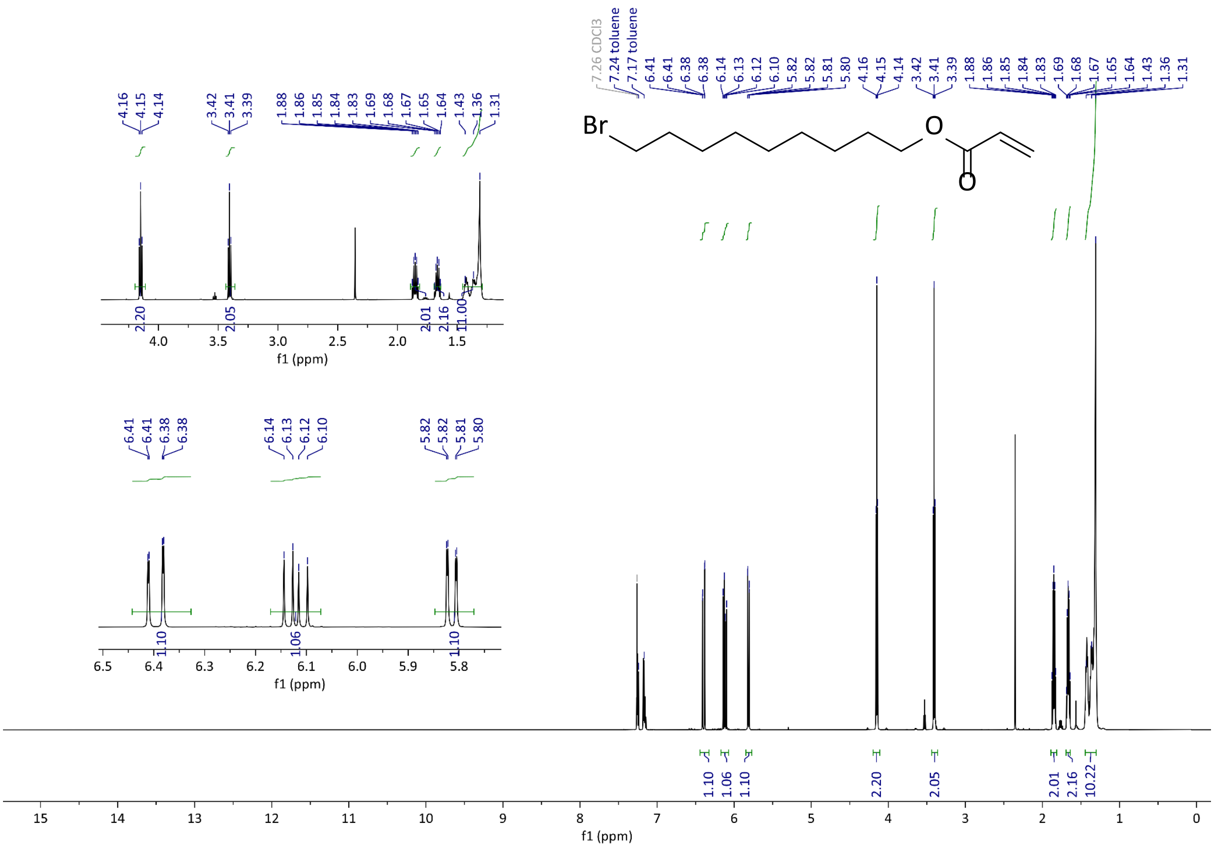

Figure S26: ^{1}H NMR spectrum of **S1** in $CDCl_3$.

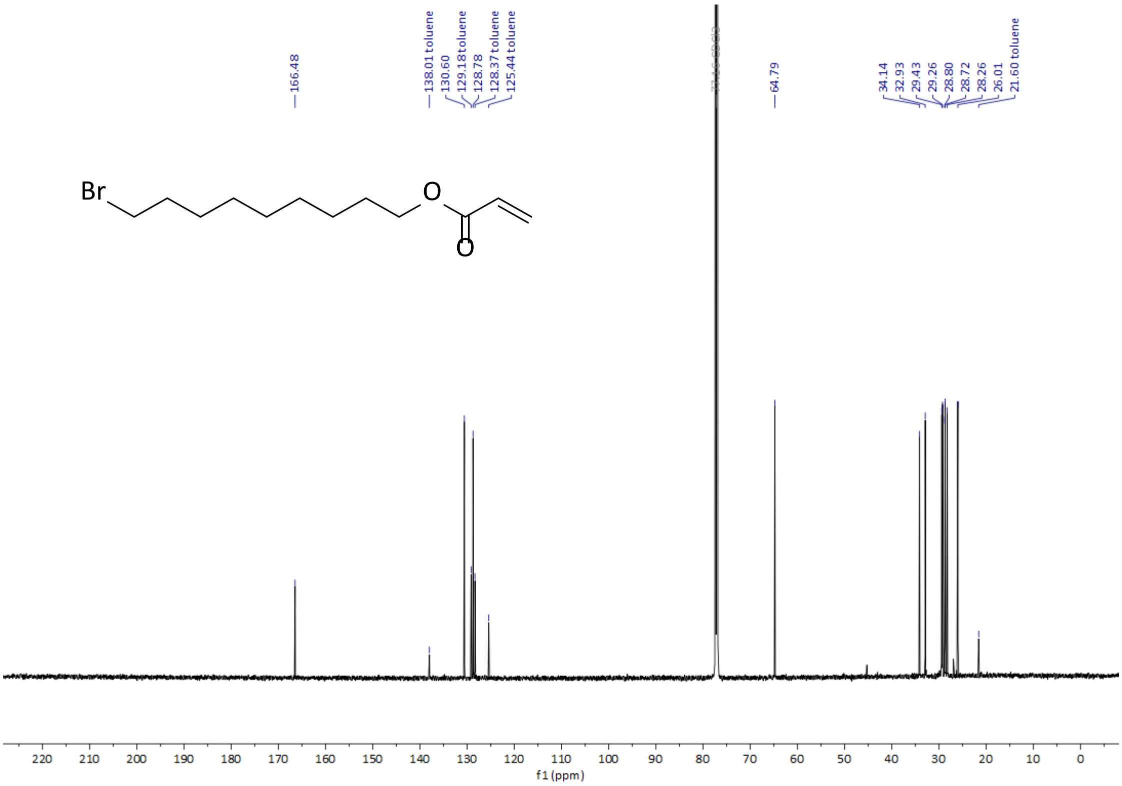

Figure S27: ^{13}C{^{1}H} NMR spectrum of **S1** in $CDCl_3$.

(*E*)-4,4'-(Diazene-1,2-diyl)bis(3,5-difluorobenzoic acid) (**2**)

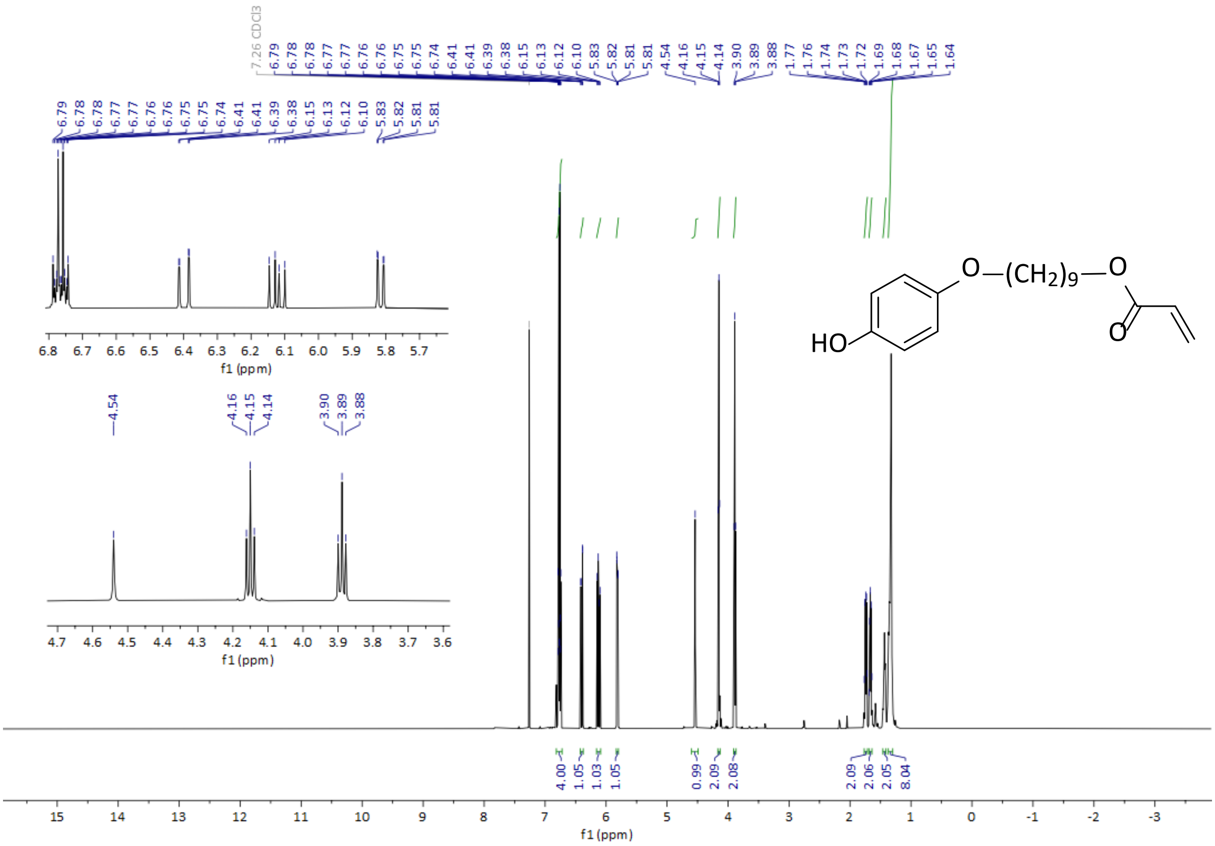

Figure S28: ^{1}H NMR spectrum of **2** in $CDCl_3$.

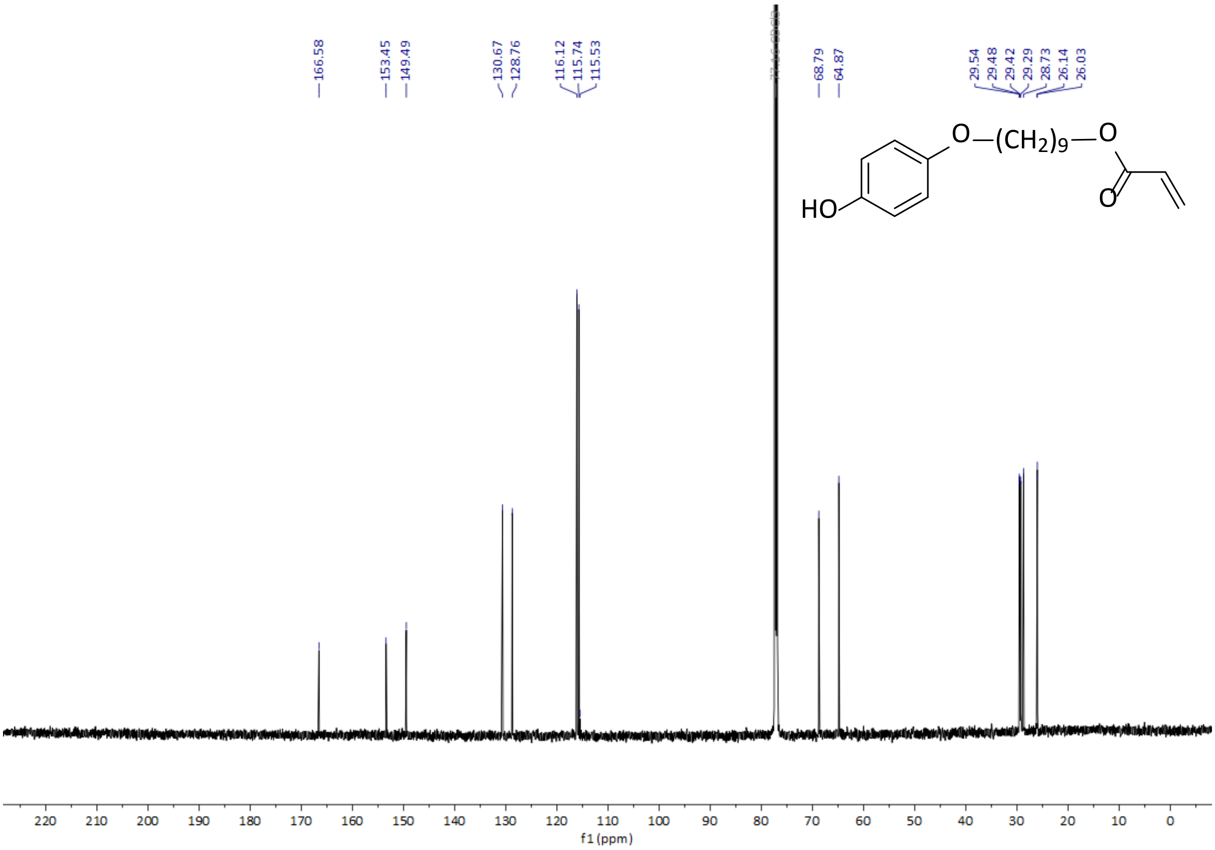

Figure S29: $^{13}C\{^{1}H\}$ NMR spectrum of **2** in $CDCl_3$.

4-Bromo-2,6-difluoroaniline (**S2**)

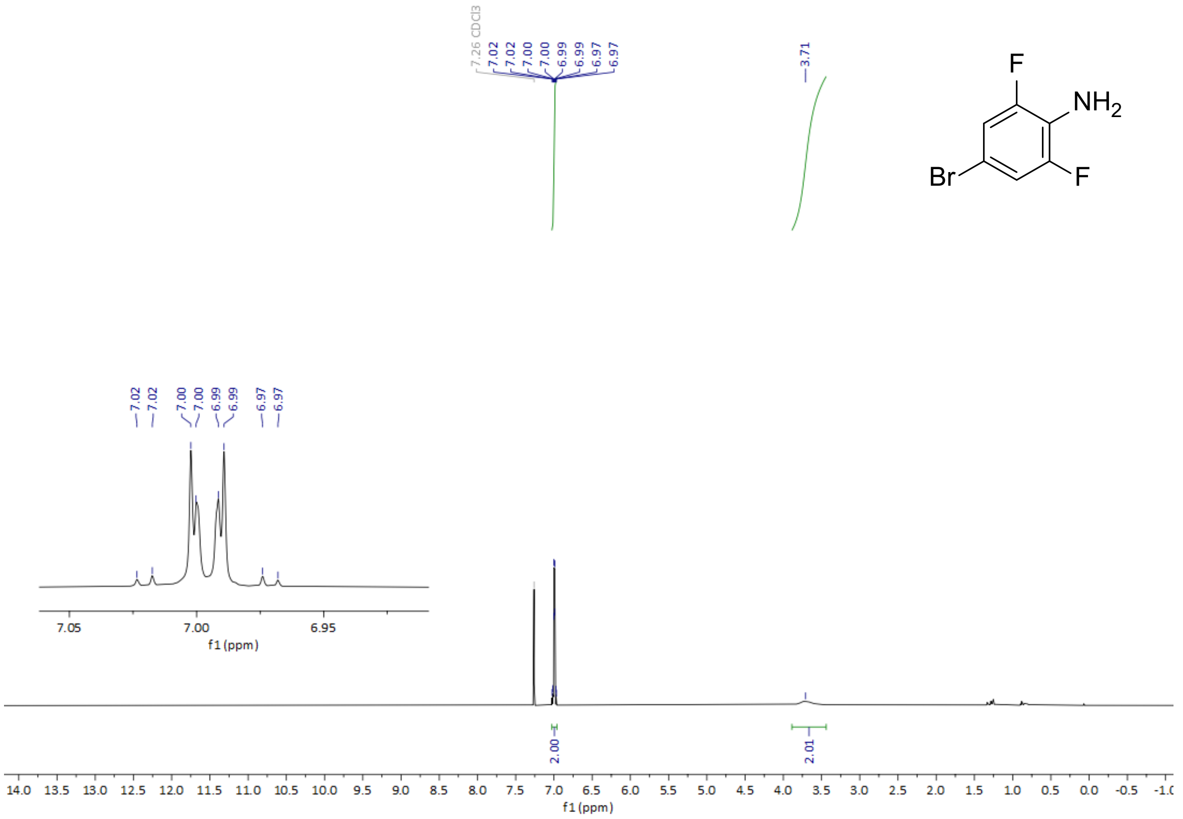

Figure S30: ^{1}H NMR spectrum of **S2** in $CDCl_3$.

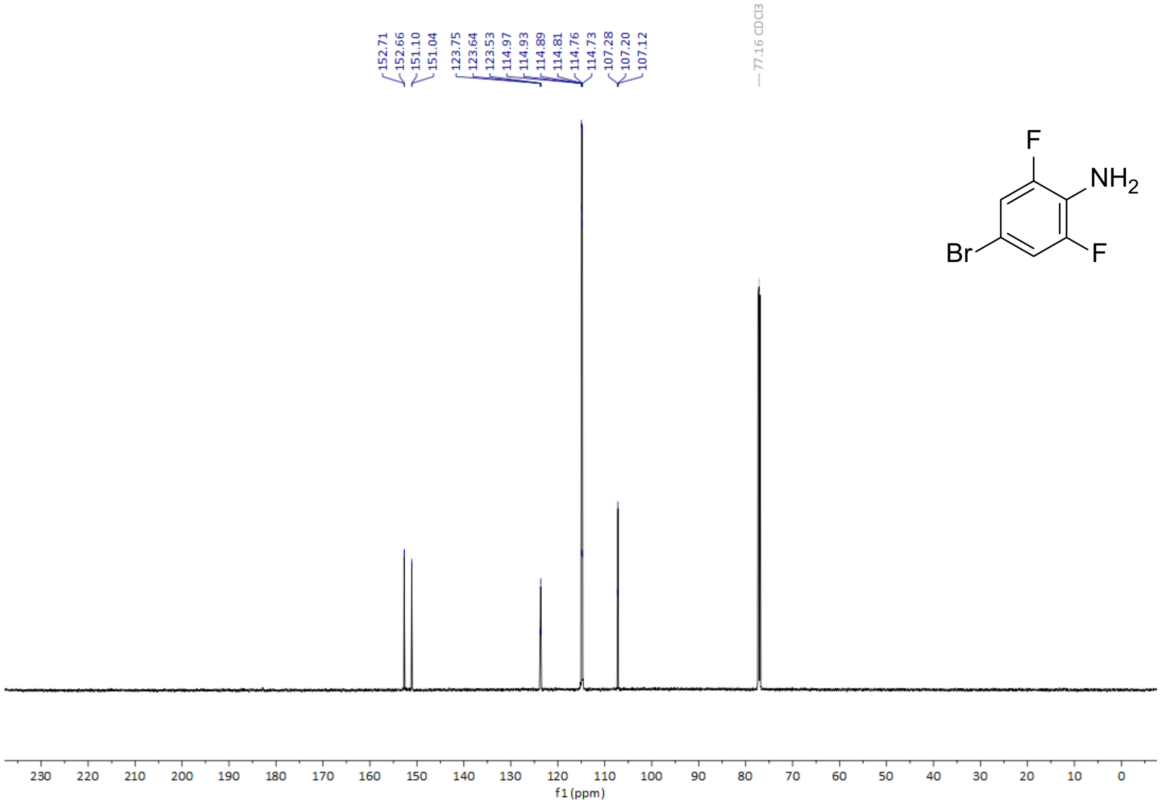

Figure S31: $^{13}C\{^{1}H\}$ NMR spectrum of **S2** in $CDCl_3$.

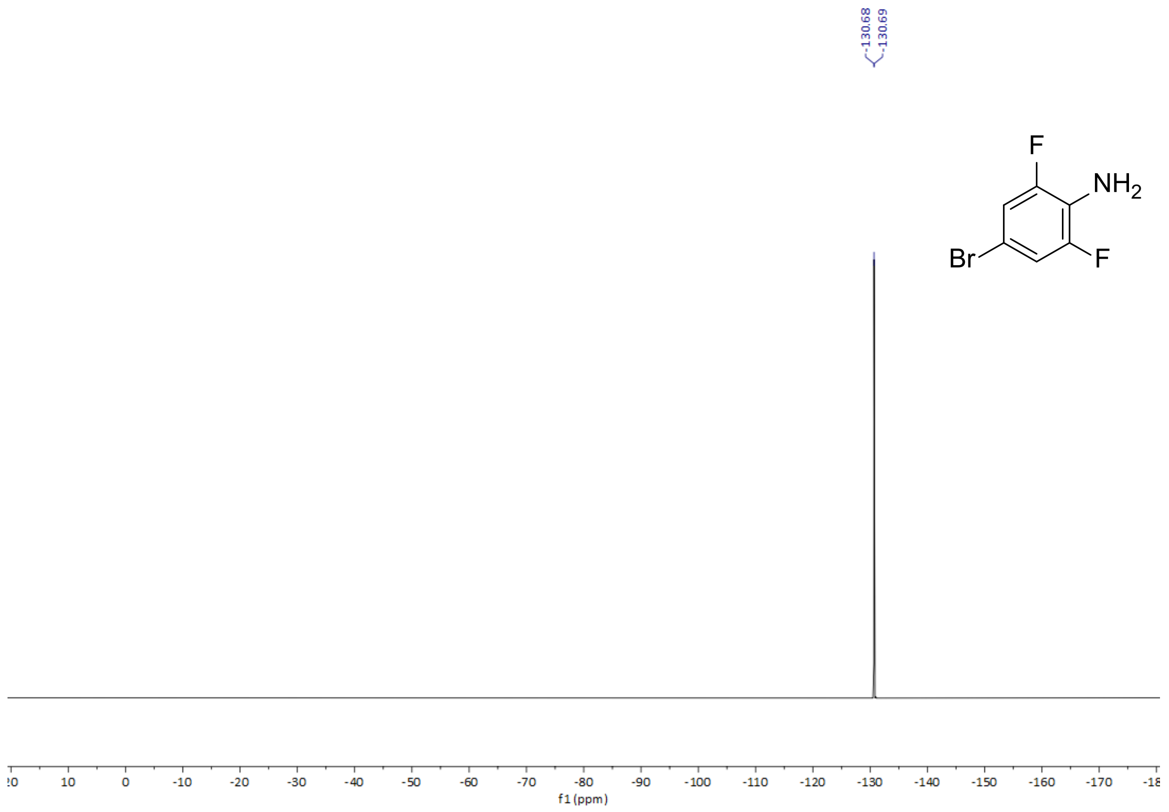

Figure S32: ^{19}F NMR spectrum of **S2** in $CDCl_3$.

4-Amino-3,5-difluorobenzonitrile (**S3**)

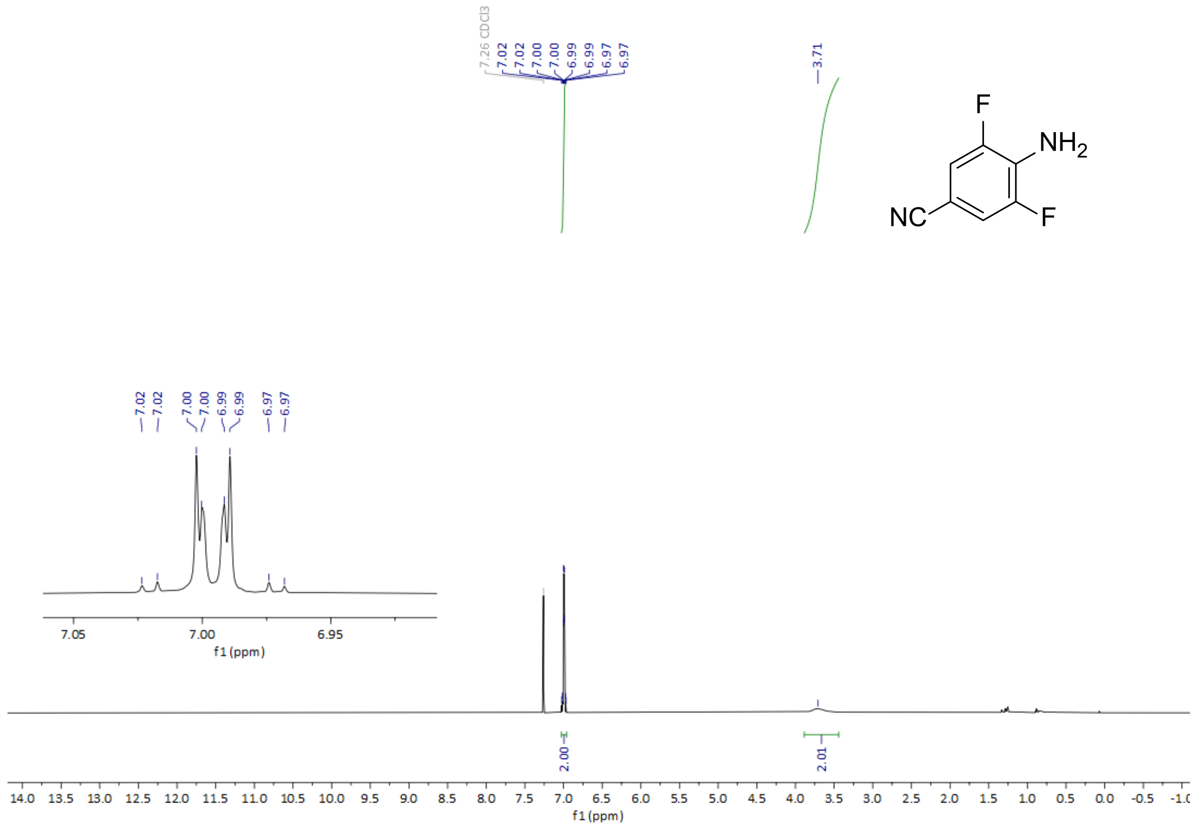

Figure S33: ^{1}H NMR spectrum of **S3** in $CDCl_3$.

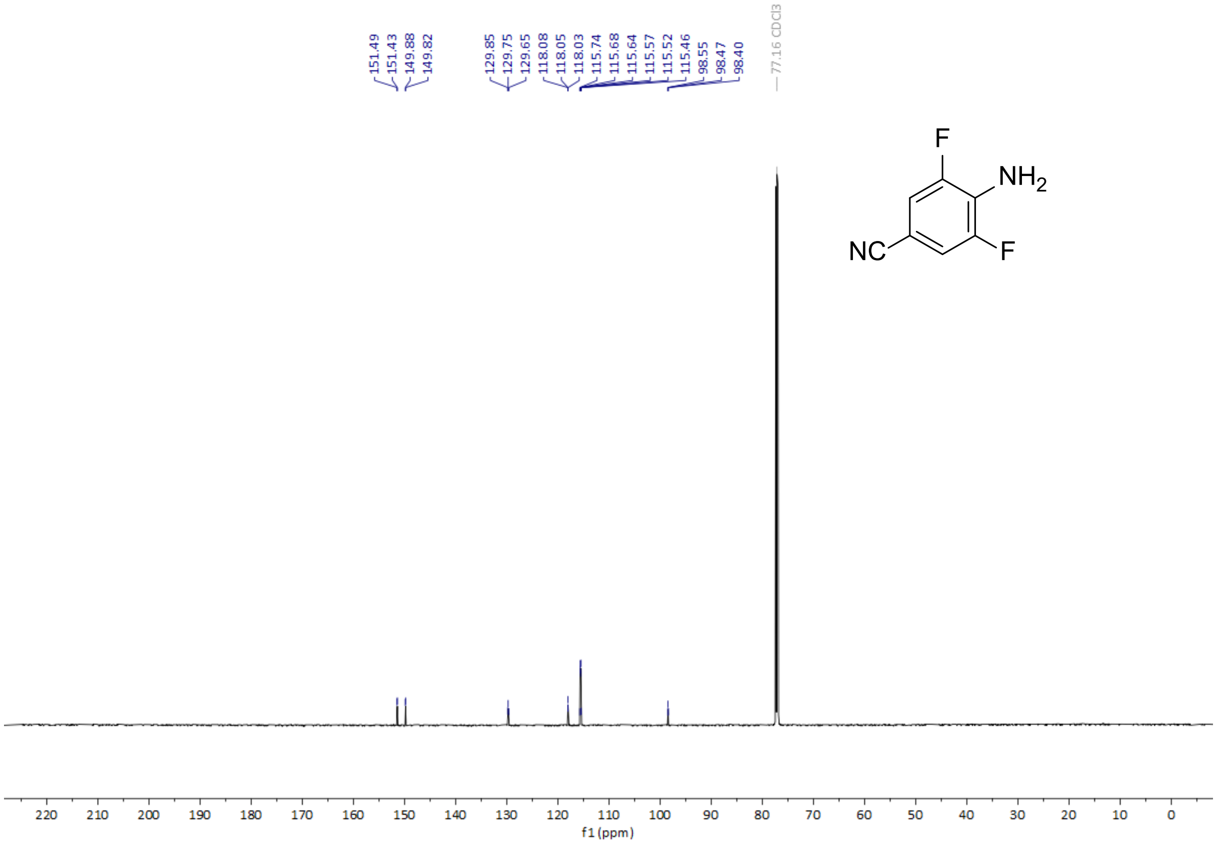

Figure S34: $^{13}C\{^{1}H\}$ NMR spectrum of **S3** in $CDCl_3$.

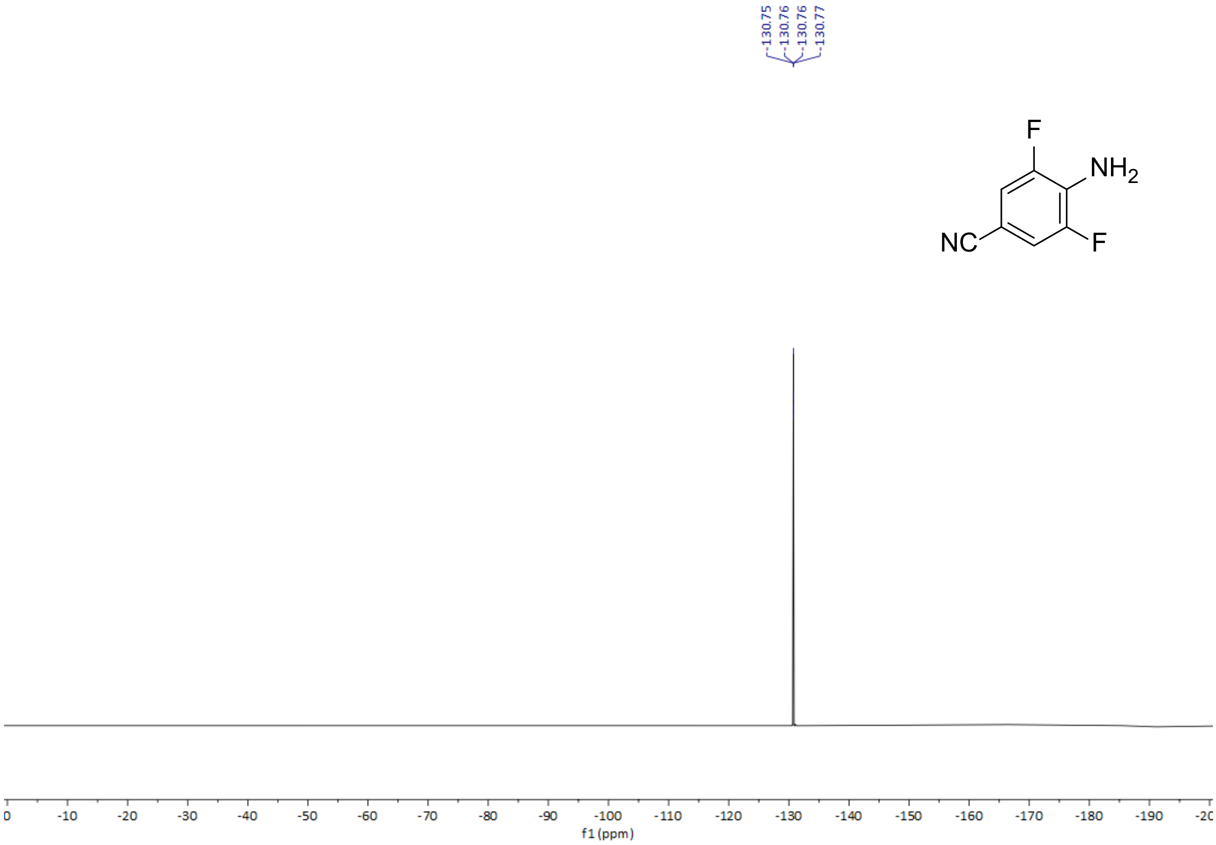

Figure S35: ^{19}F NMR spectrum of **S3** in $CDCl_3$.

4-Amino-3,5-difluorobenzoic acid (**S4**)

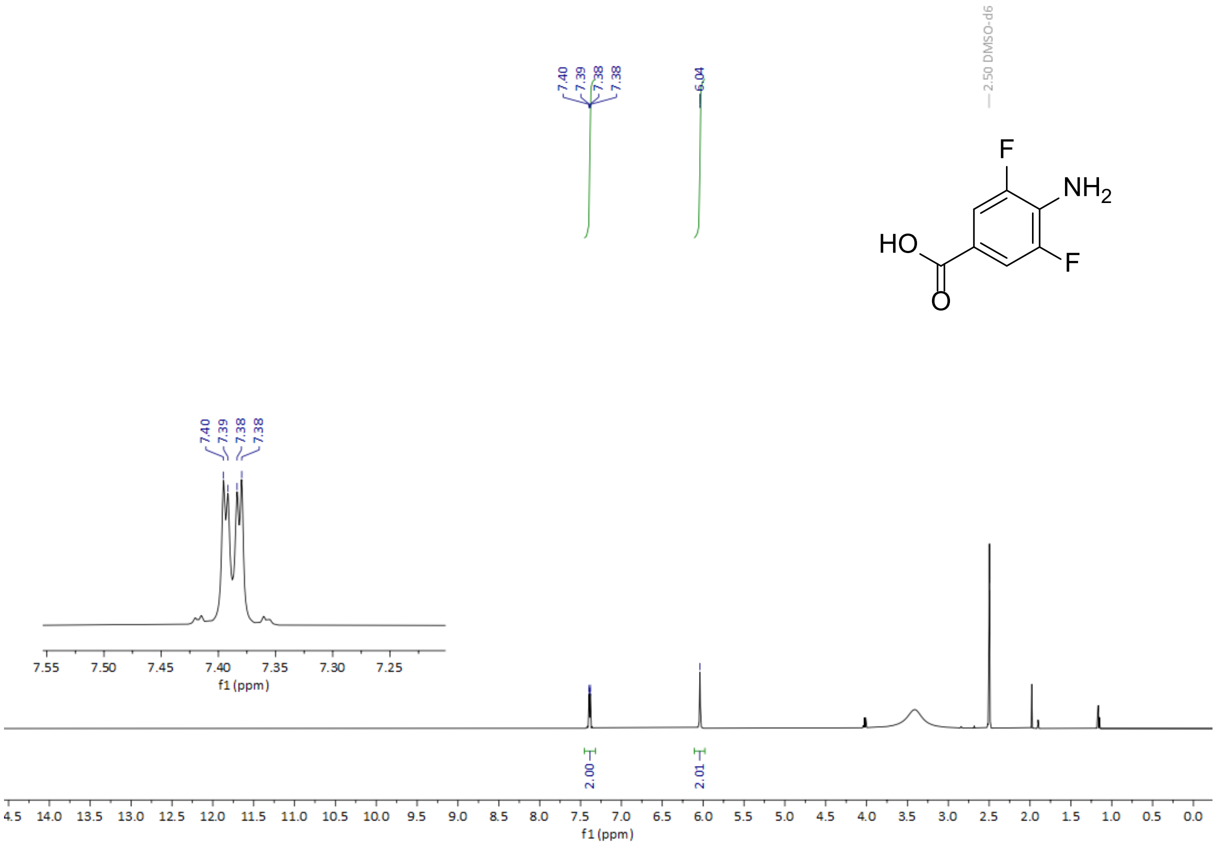

Figure S36: ^{1}H NMR spectrum of **S4** in DMSO-d_6.

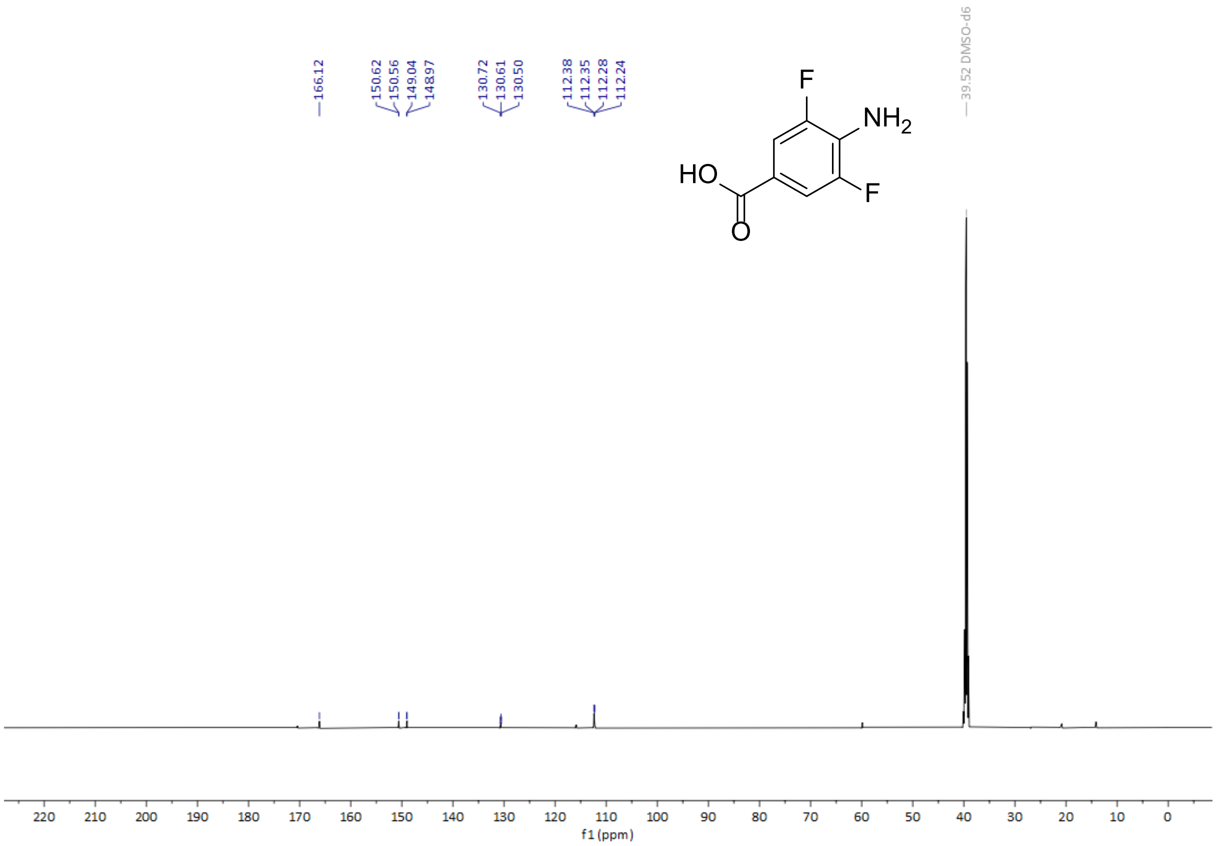

Figure S37: ^{13}C{^{1}H} NMR spectrum of **S4** in DMSO-d_6.

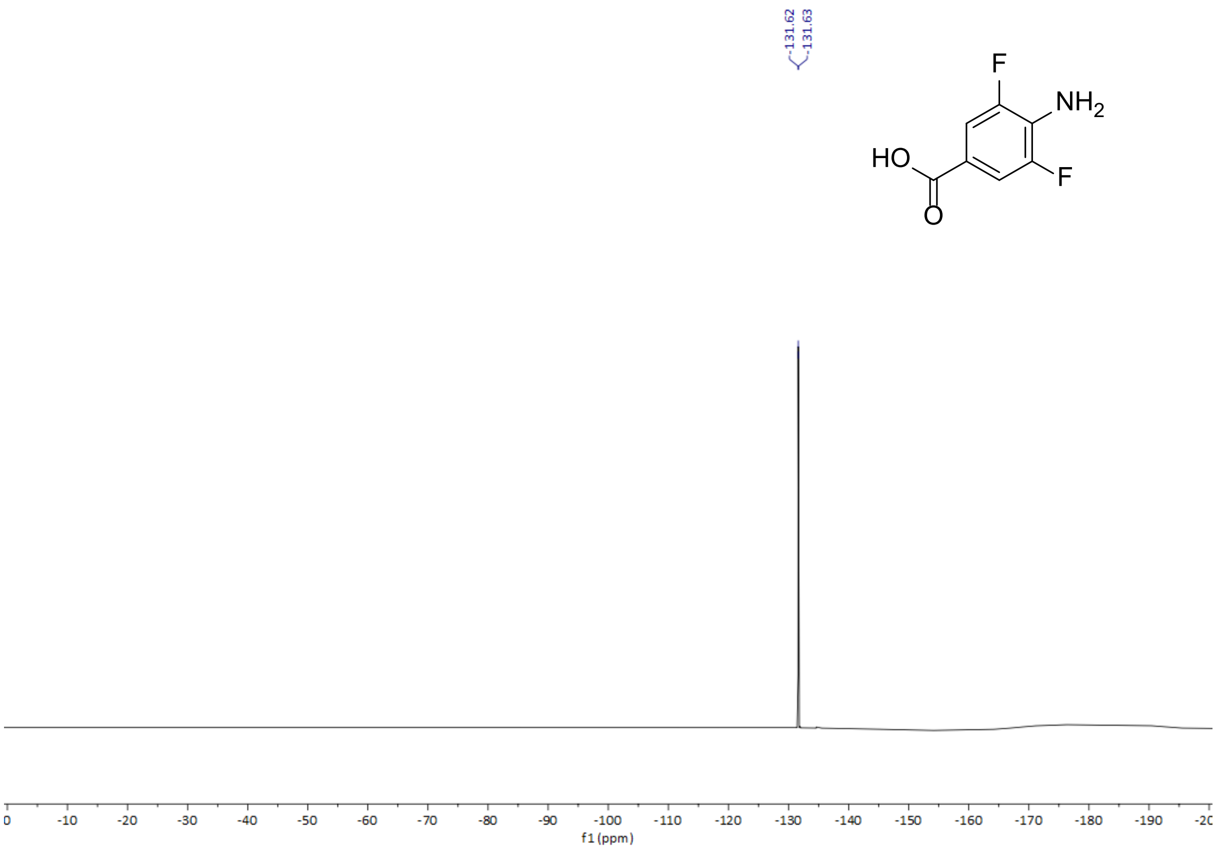

Figure S38: ^{19}F NMR spectrum of **S4** in DMSO-d_6.

Ethyl 4-amino-3,5-difluorobenzoate (**S5**)

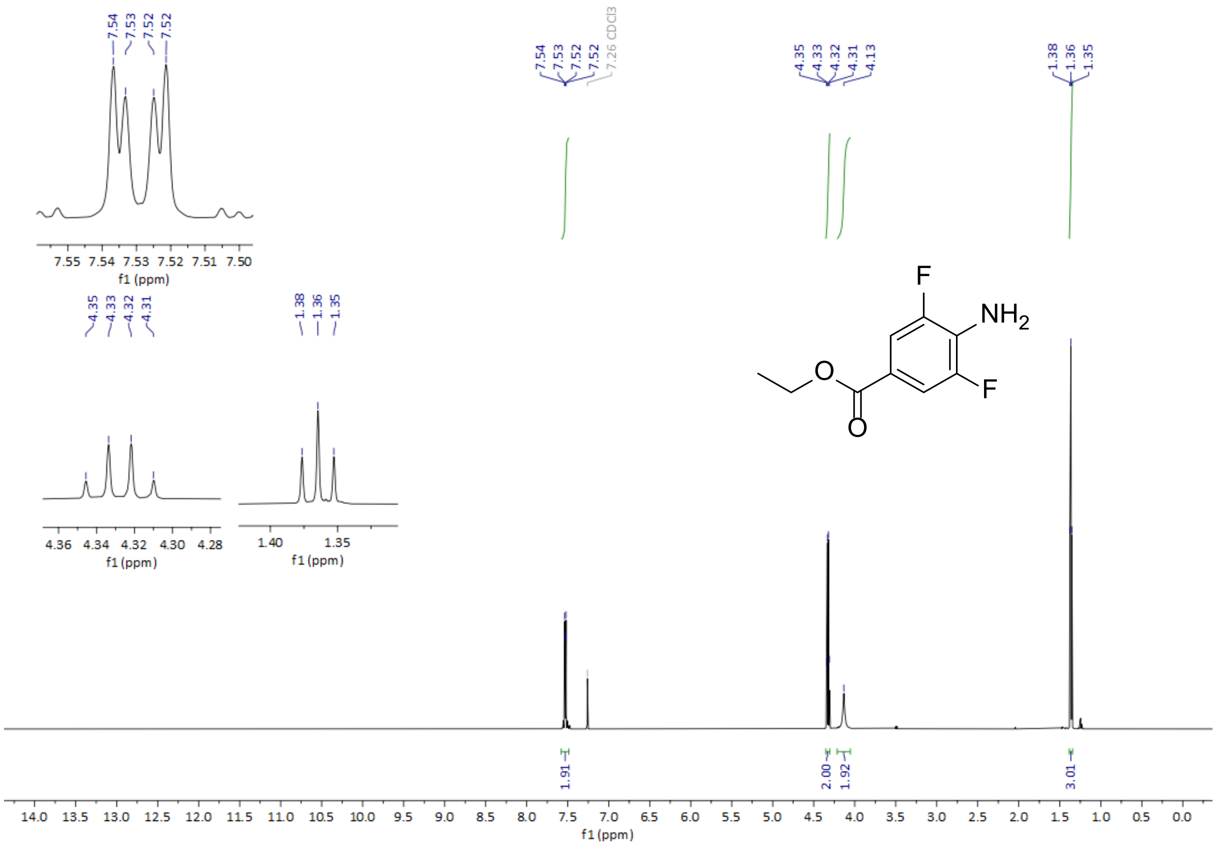

Figure S39: ^{1}H NMR spectrum of **S5** in $CDCl_3$.

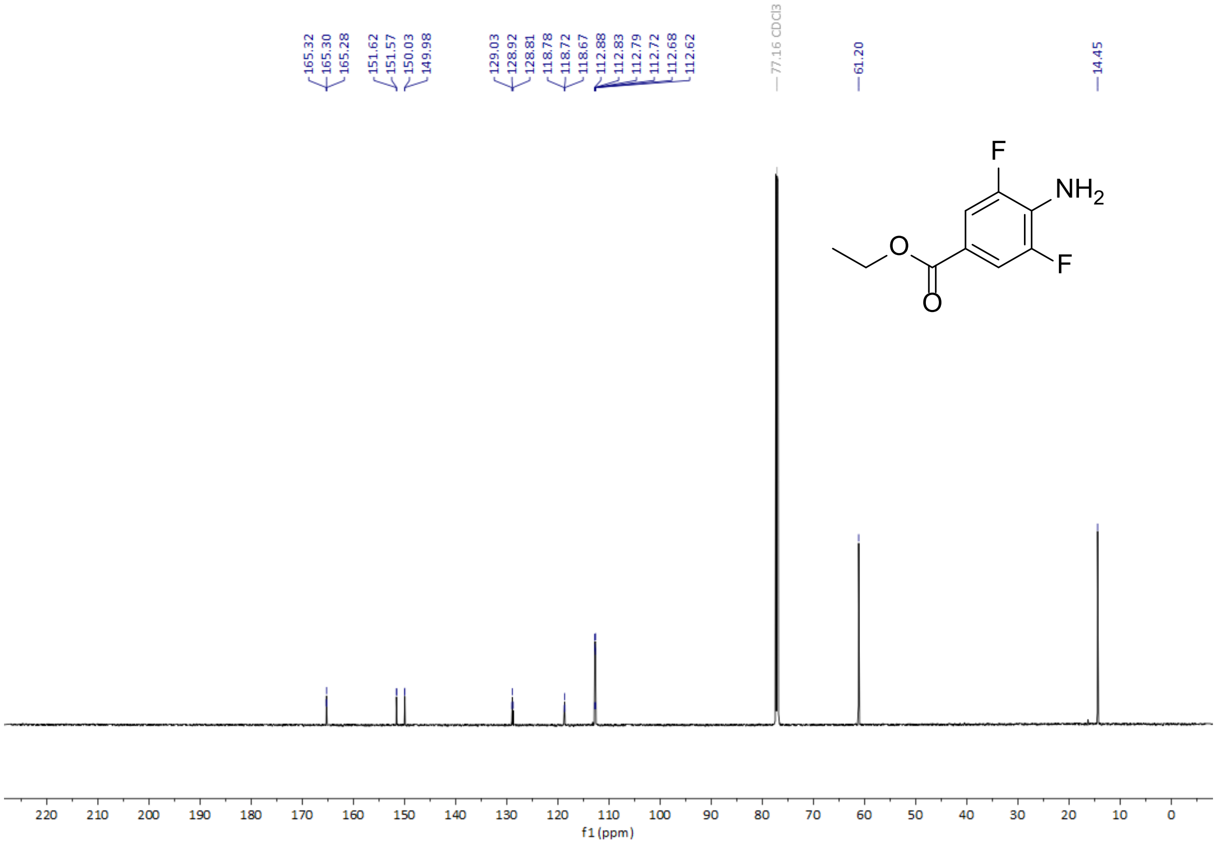

Figure S40: $^{13}C\{^1H\}$ NMR spectrum of **S5** in $CDCl_3$.

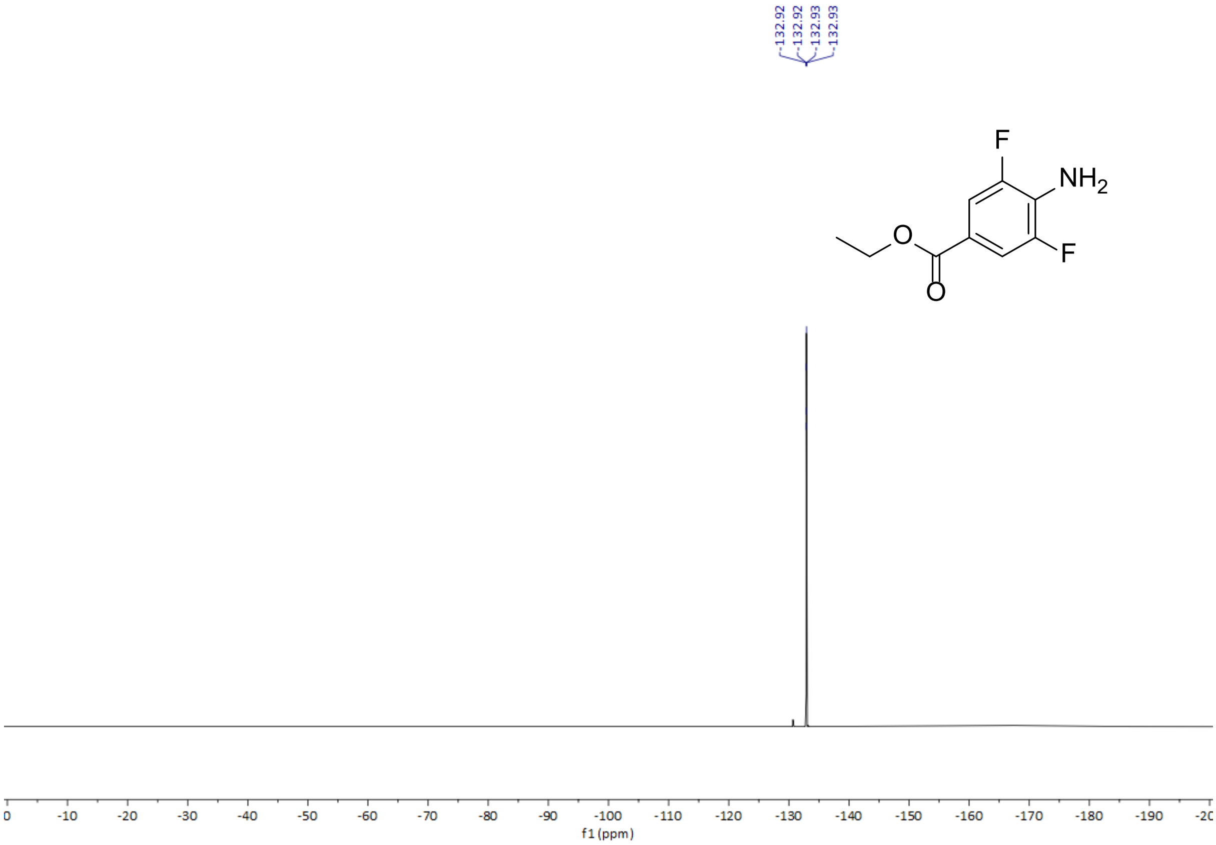

Figure S41: ^{19}F NMR spectrum of **S5** in $CDCl_3$.

Diethyl 4,4'-(diazene-1,2-diyl)(E)-bis(3,5-difluorobenzoate) (**S6**)

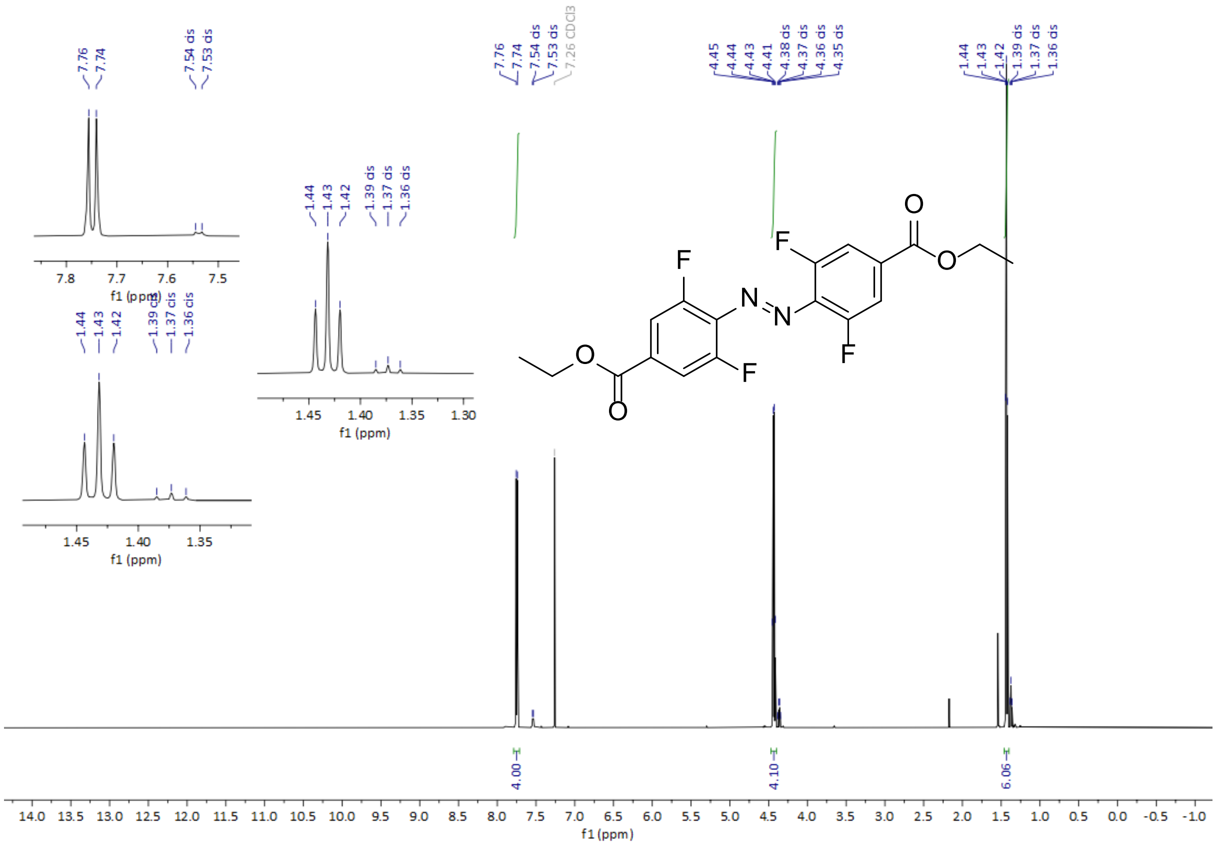

Figure S42: ^{1}H NMR spectrum of **S6** in $CDCl_3$.

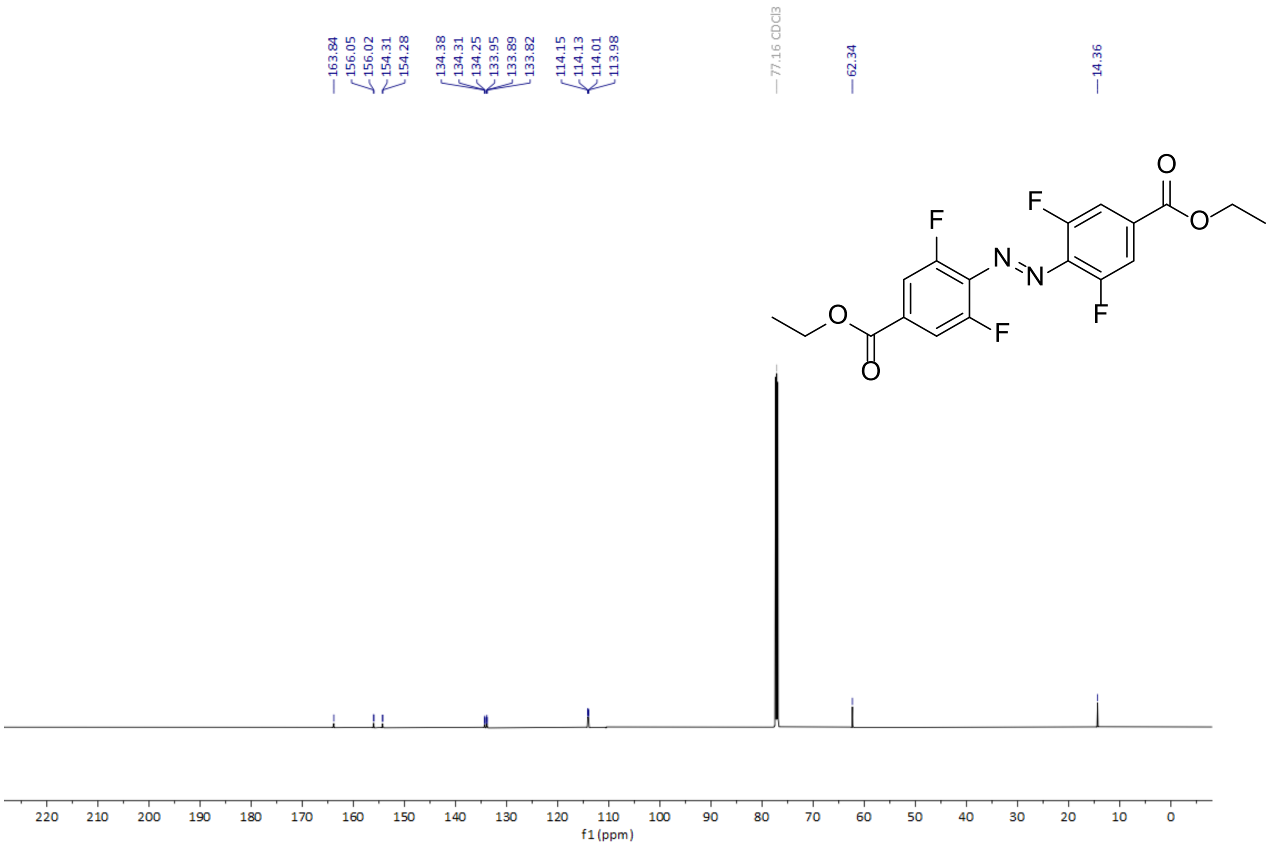

Figure S43: $^{13}C\{^{1}H\}$ NMR spectrum of **S6** in $CDCl_3$.

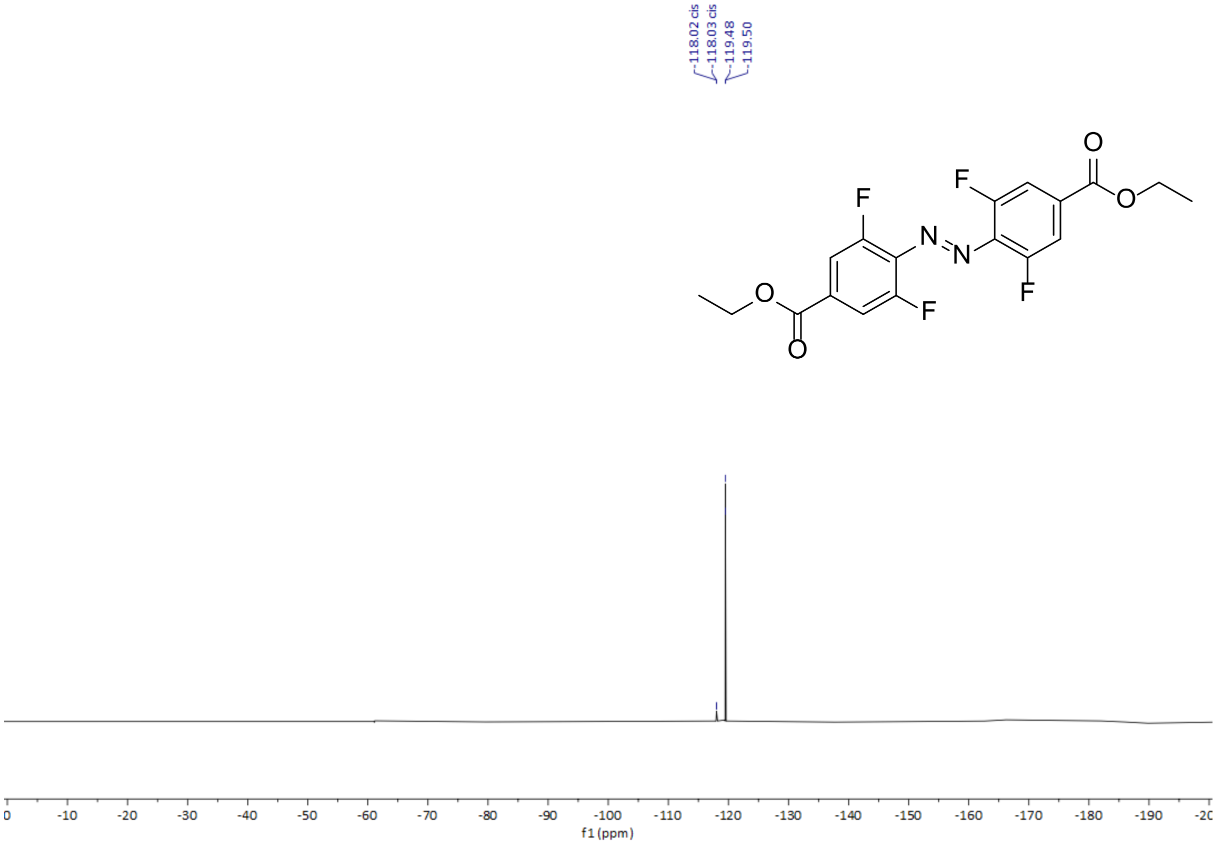

Figure S44: ^{19}F NMR spectrum of **S6** in $CDCl_3$.

(*E*)-4,4'-(Diazene-1,2-diyl)bis(3,5-difluorobenzoic acid) (**S7**)

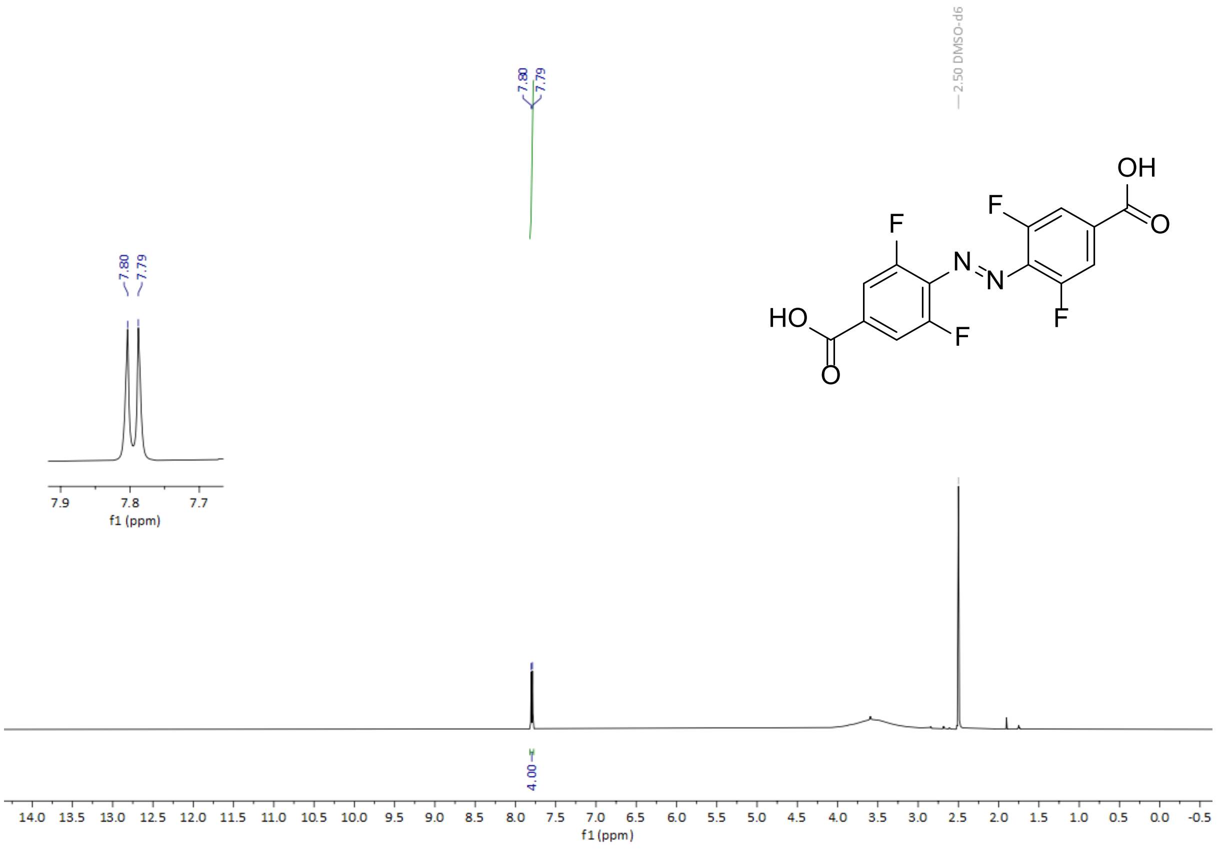

Figure S45: ^{1}H NMR spectrum of **S7** in DMSO-d_6.

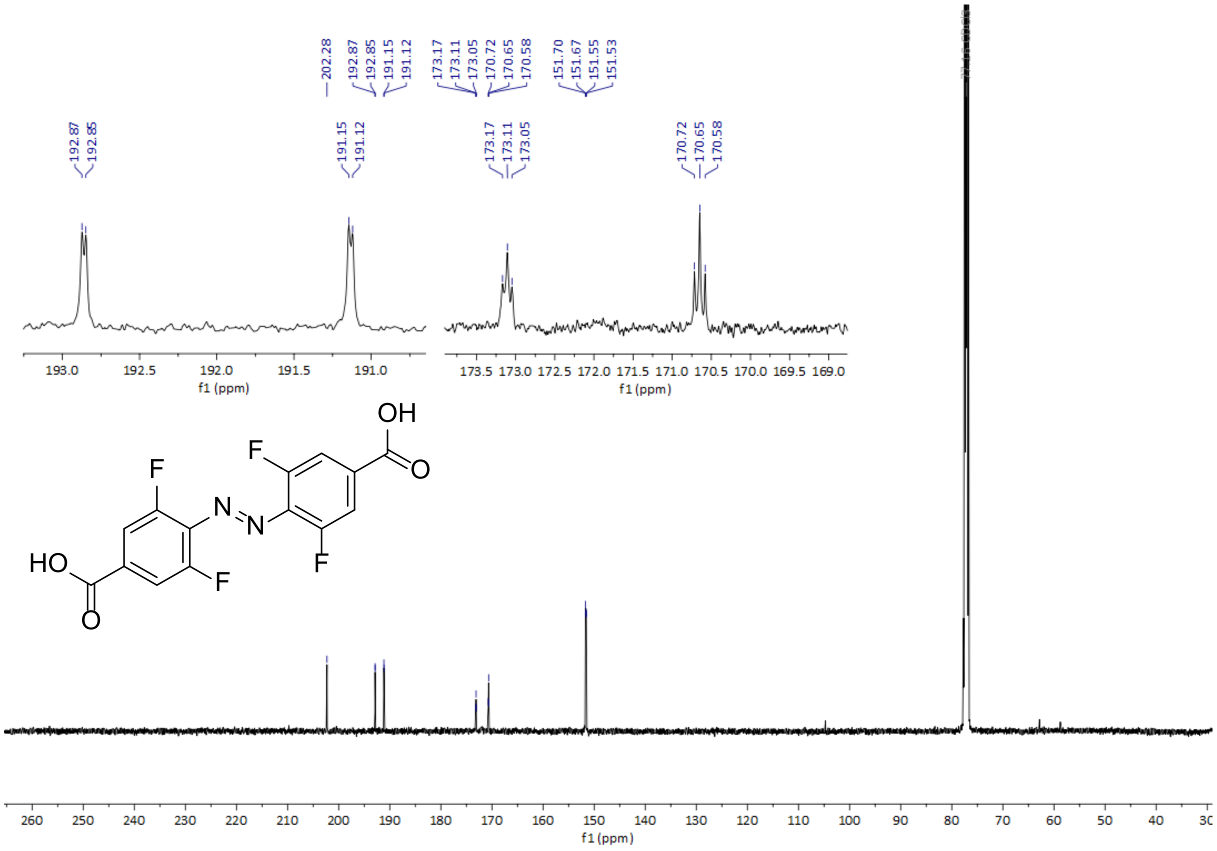

Figure S46: $^{13}C\{^{1}H\}$ NMR spectrum of **S7** in DMSO-d_6.

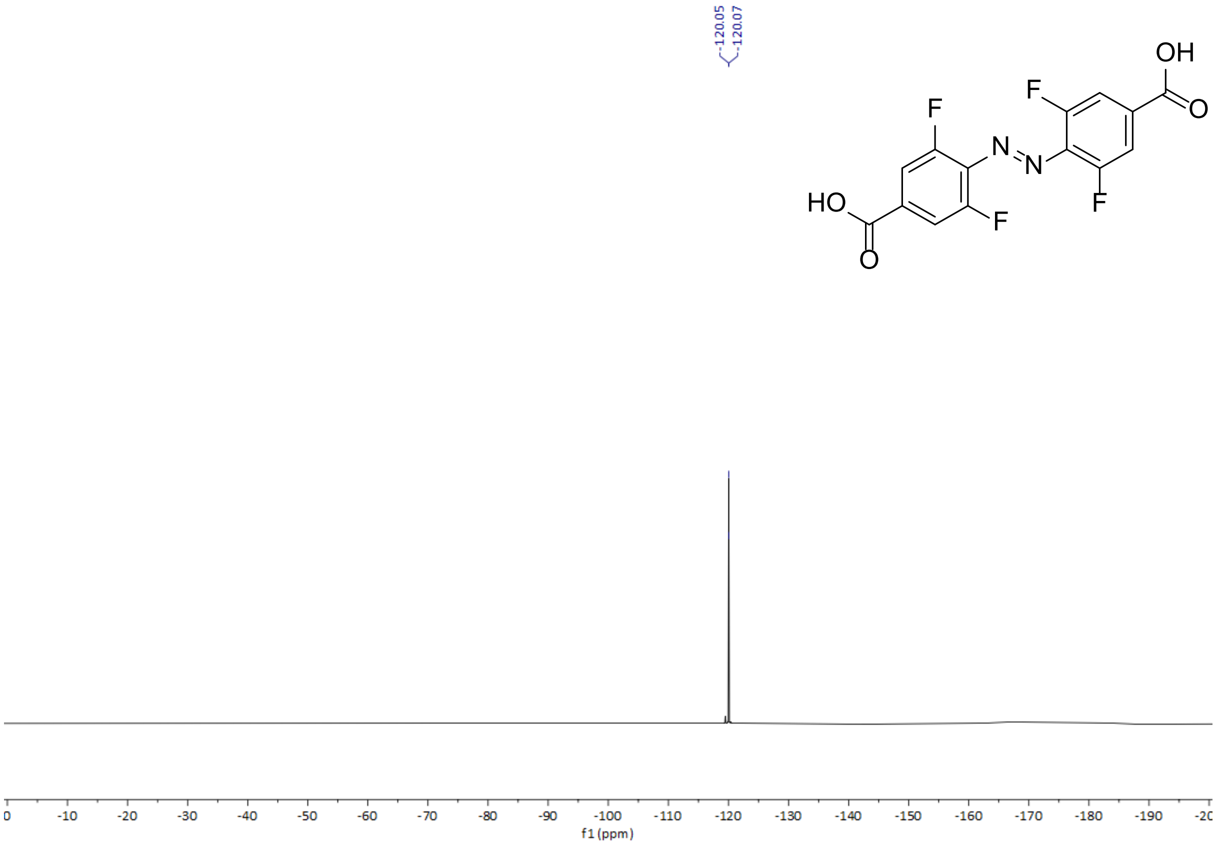

Figure S47: ^{19}F NMR spectrum of **S7** in DMSO-d_6.

(*E*)-4,4'-(Diazene-1,2-diyl)bis(3,5-difluorobenzoyl chloride) **(4)**

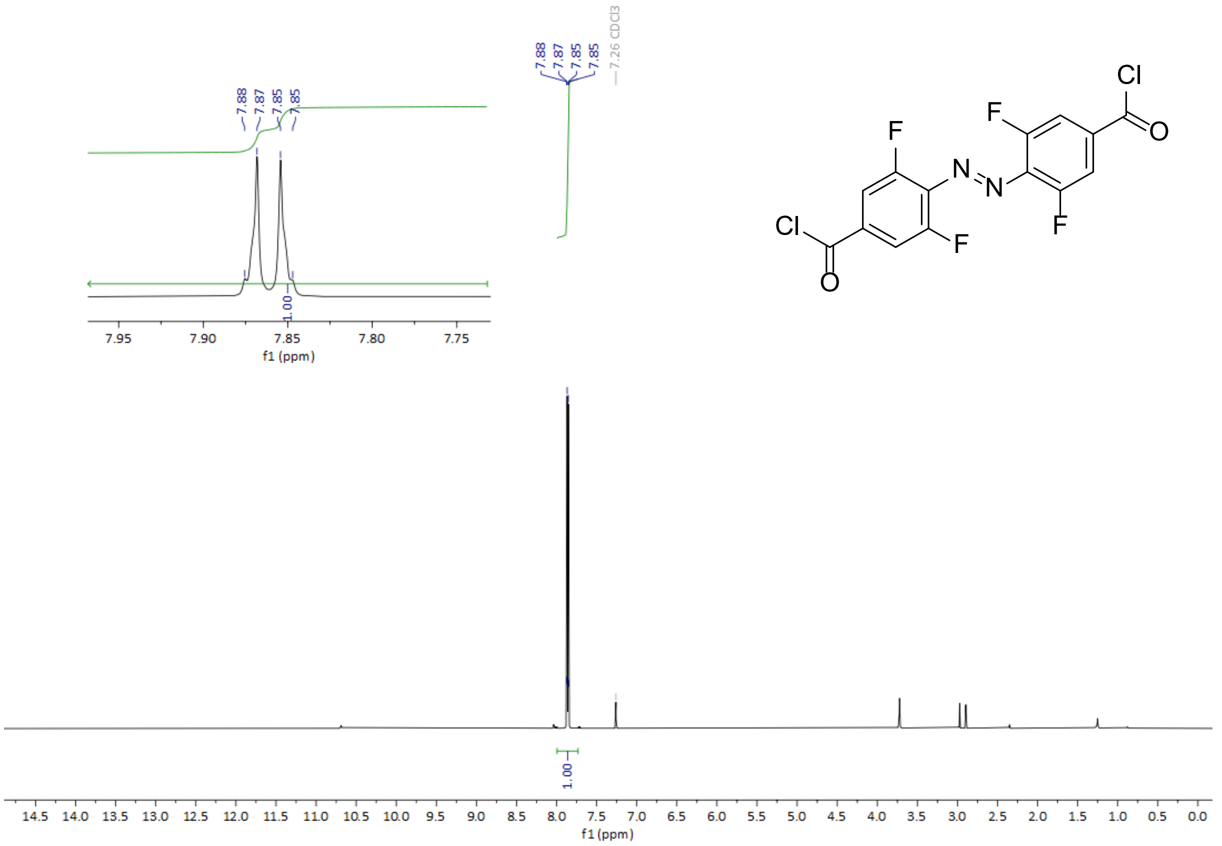

Figure S48: ^{1}H NMR spectrum of **4** in $CDCl_3$.

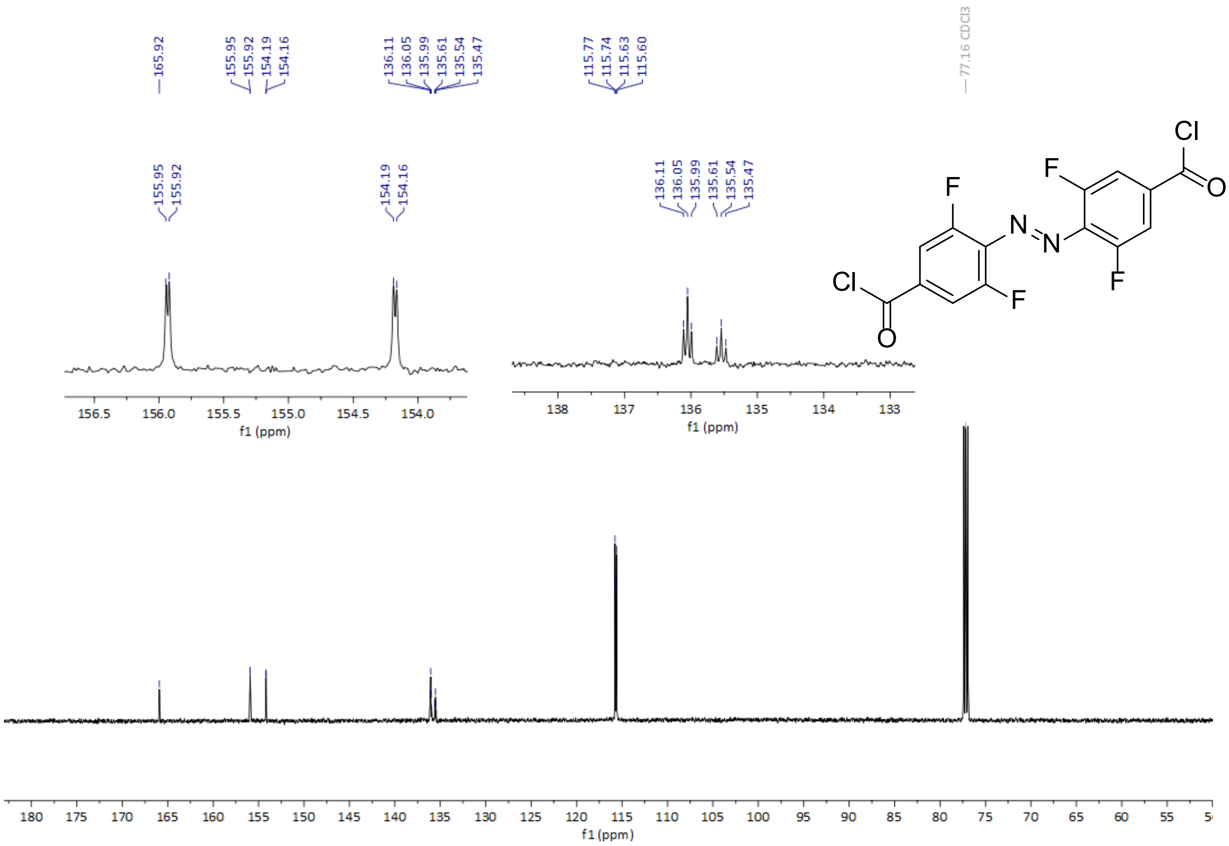

Figure S49: $^{13}C\{^{1}H\}$ NMR spectrum of **4** in $CDCl_3$.

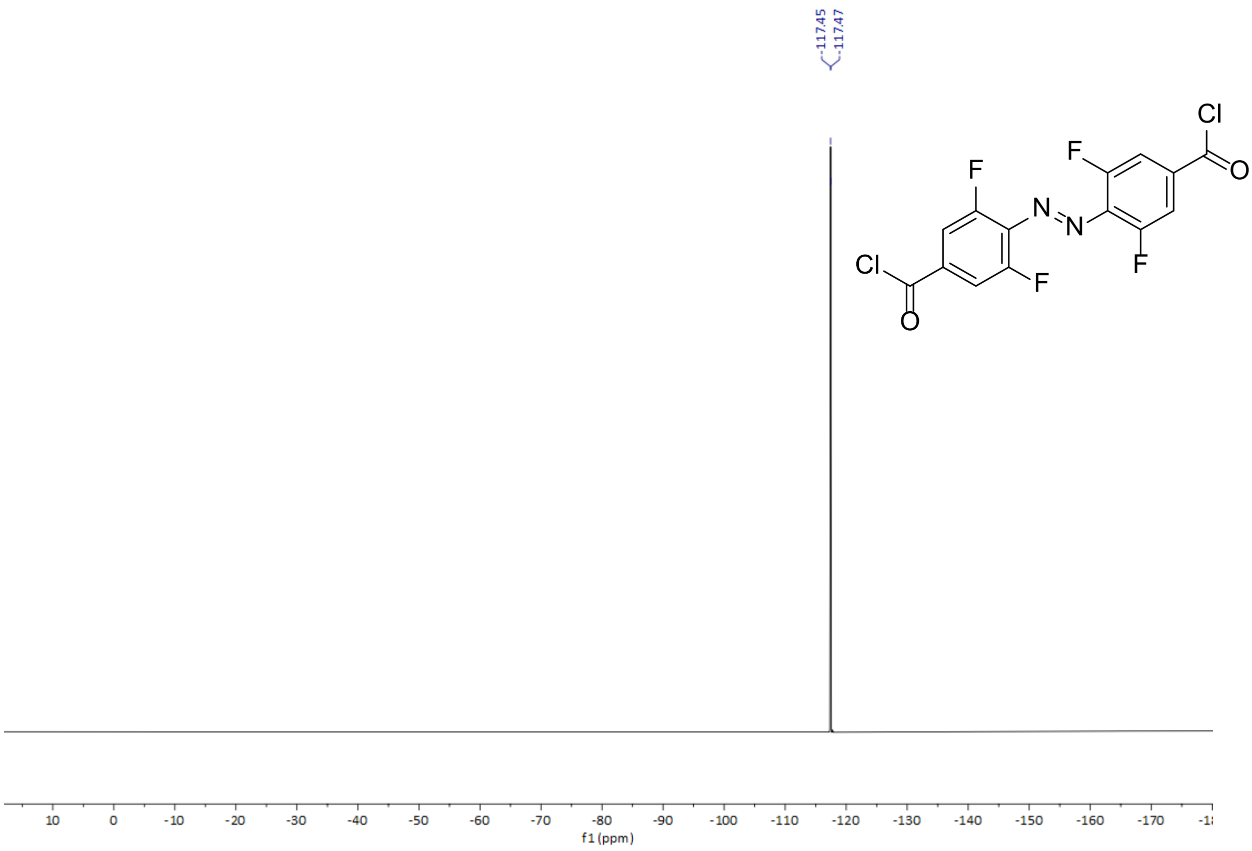

Figure S50: ^{19}F NMR spectrum of **4** in $CDCl_3$.

(*E*)-4,4'-(Diazene-1,2-diyl)bis(3,5-difluorobenzoic acid) (**5**)

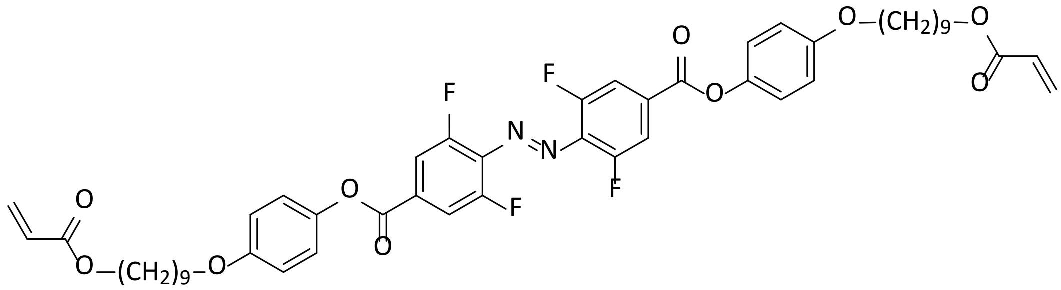

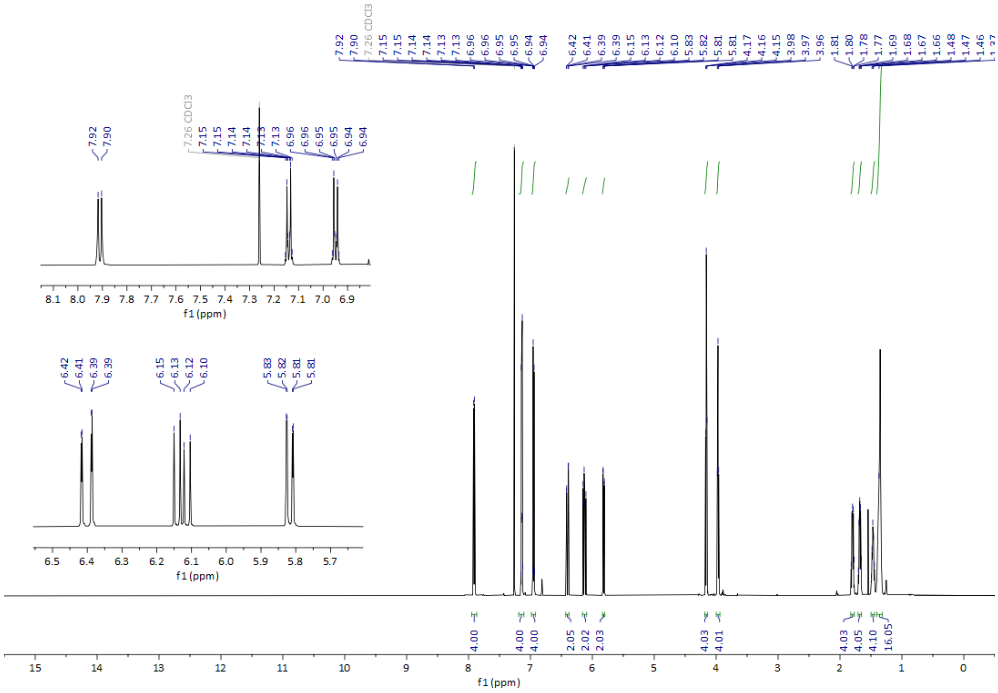

Figure S51: ^{1}H NMR spectrum of **5** in $CDCl_3$.

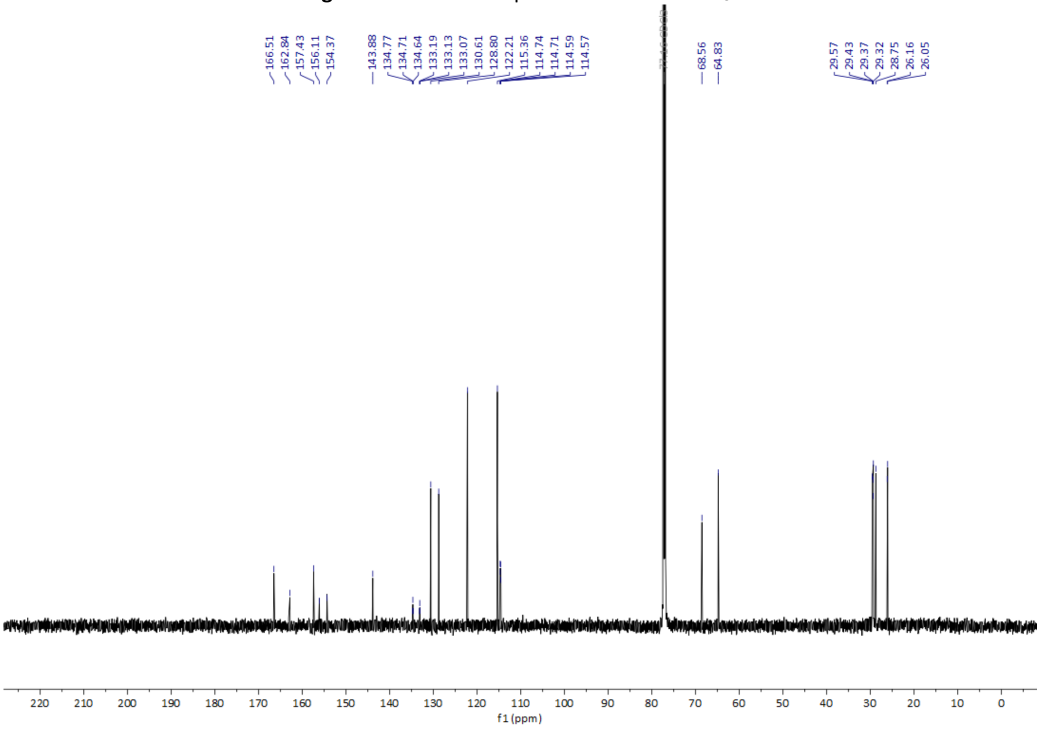

Figure S52: $^{13}C\{^{1}H\}$ NMR spectrum of **5** in $CDCl_3$.

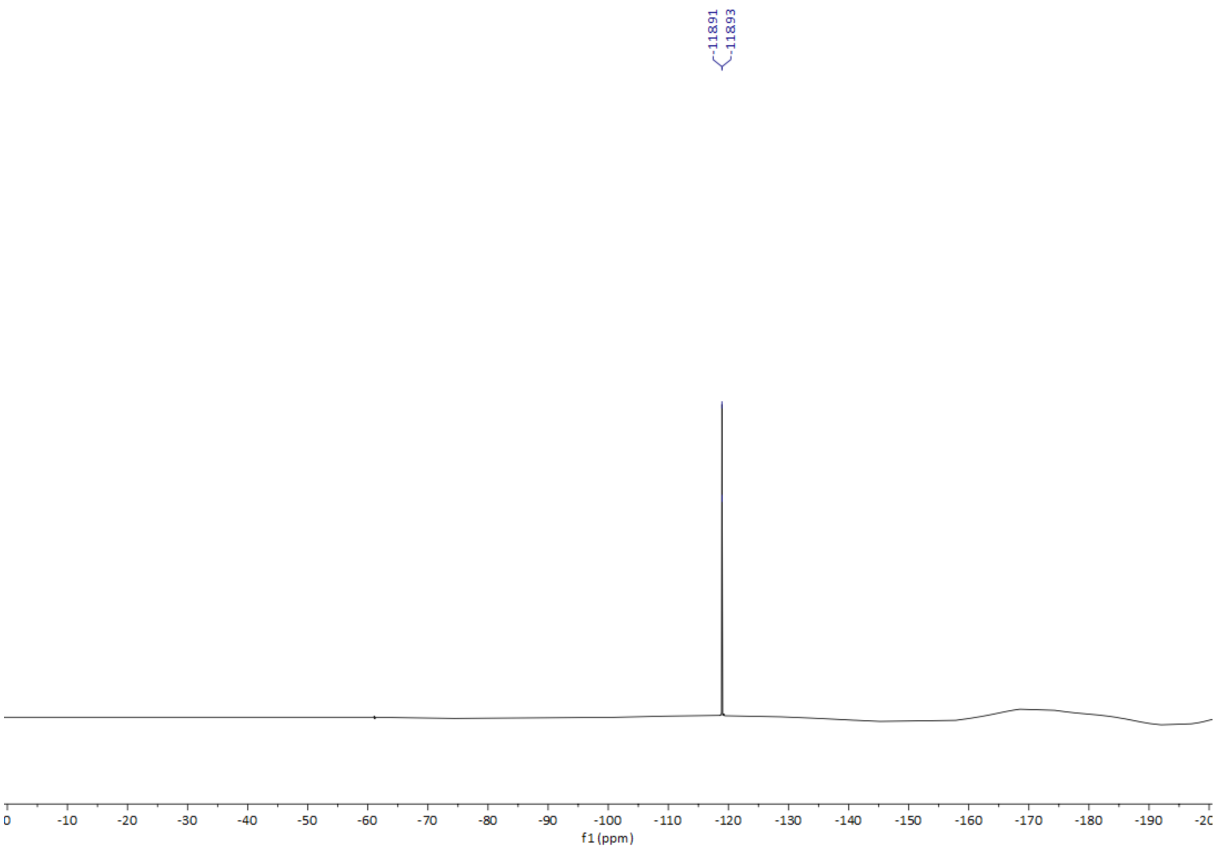

Figure S53: ^{19}F NMR spectrum of **5** in $CDCl_3$.

Bis(9-(acryloyloxy)nonyl) 4,4'-(diazene-1,2-diyl)(E)-bis(3,5-difluorobenzoate) (**S8**)

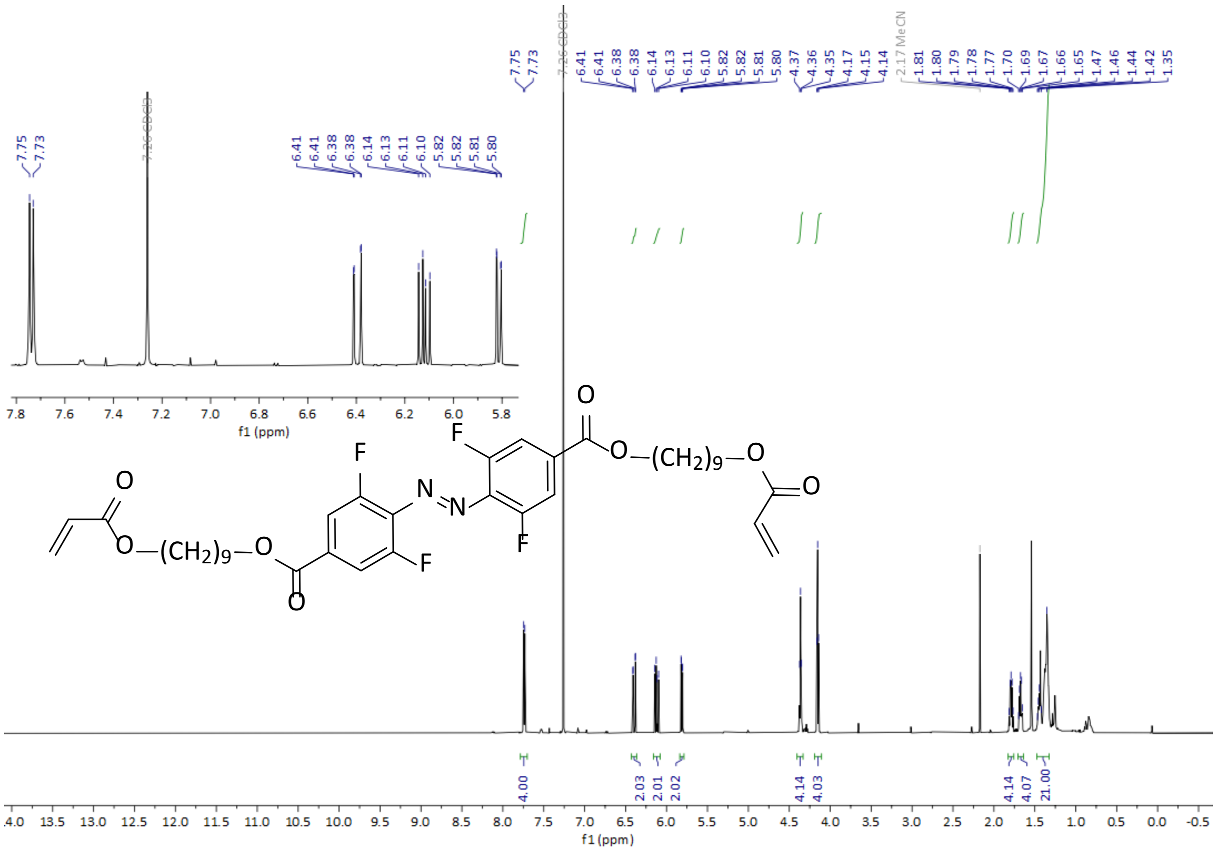

Figure S54: ^{1}H NMR spectrum of **S8** in $CDCl_3$.

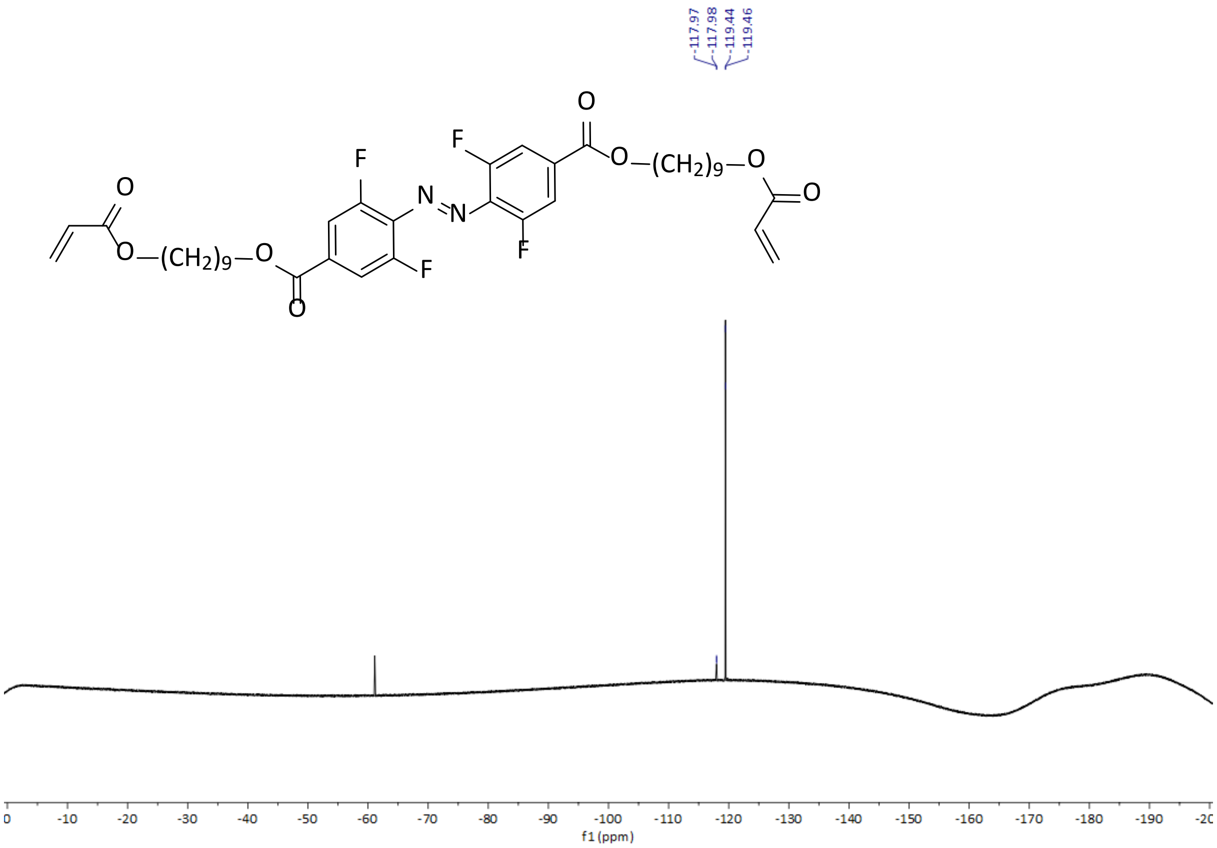

Figure S55: ^{19}F NMR spectrum of **S8** in $CDCl_3$.

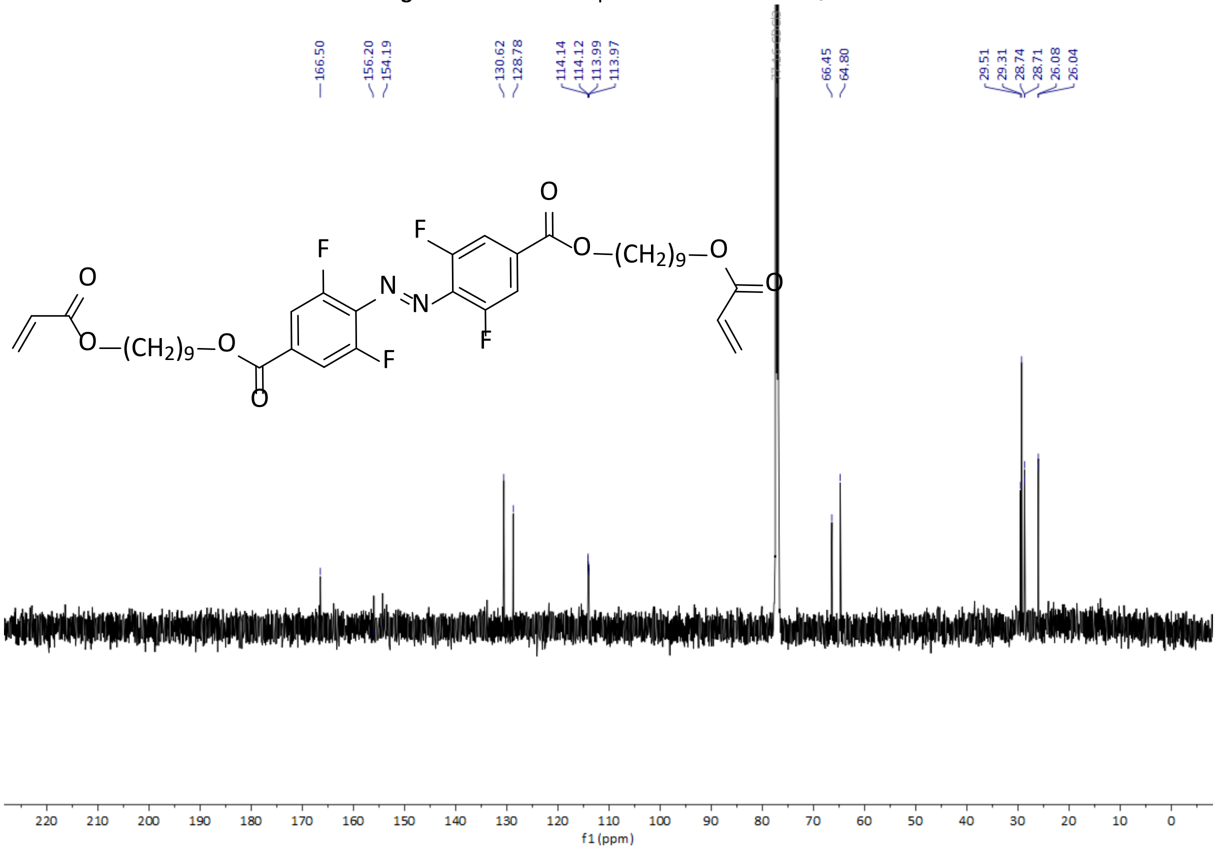

Figure S56: $^{13}C\{^1H\}$ NMR spectrum of **S8** in $CDCl_3$.

Bis(4-hexylphenyl) 4,4'-(diazene-1,2-diyl)(E)-bis(3,5-difluorobenzoate) (**6**)

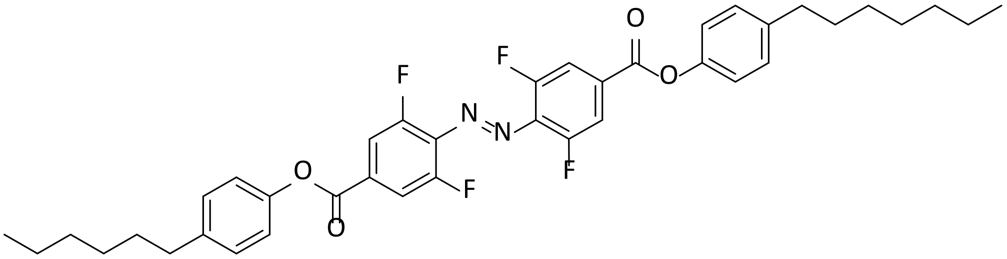

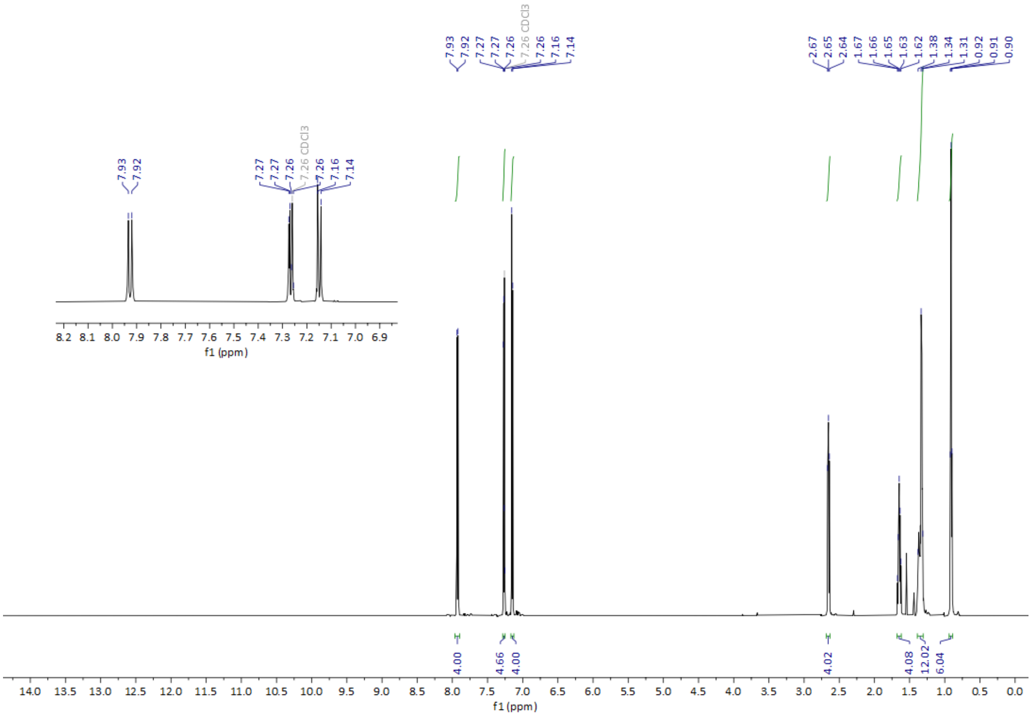

Figure S57: ^{1}H NMR spectrum of **6** in $CDCl_3$.

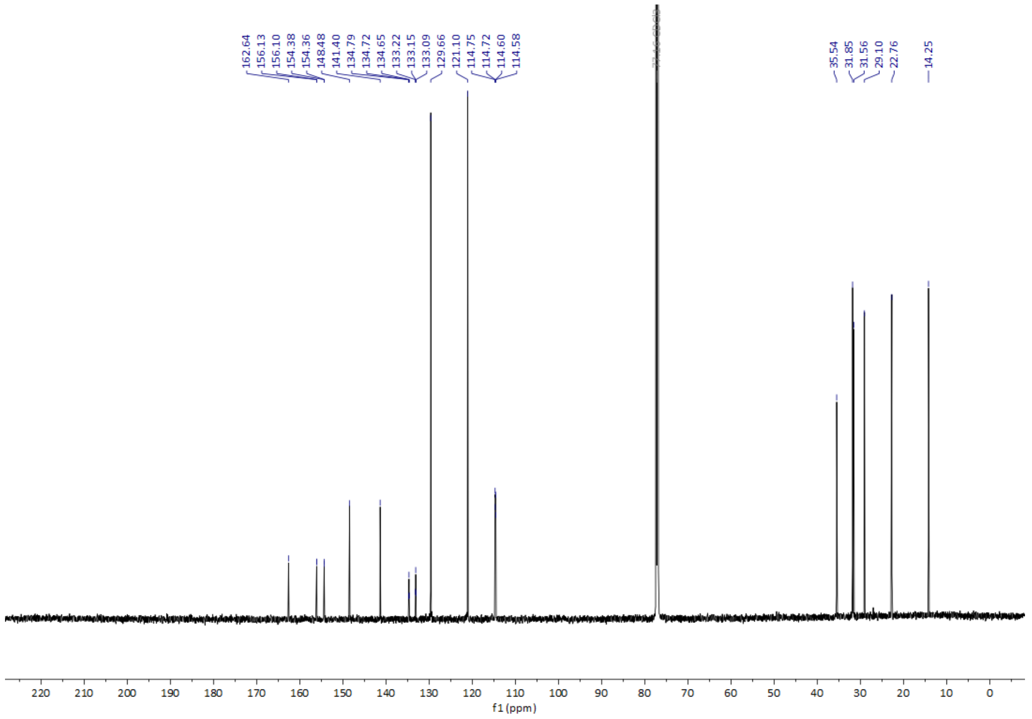

Figure S58: $^{13}C\{^{1}H\}$ NMR spectrum of **6** in $CDCl_3$.

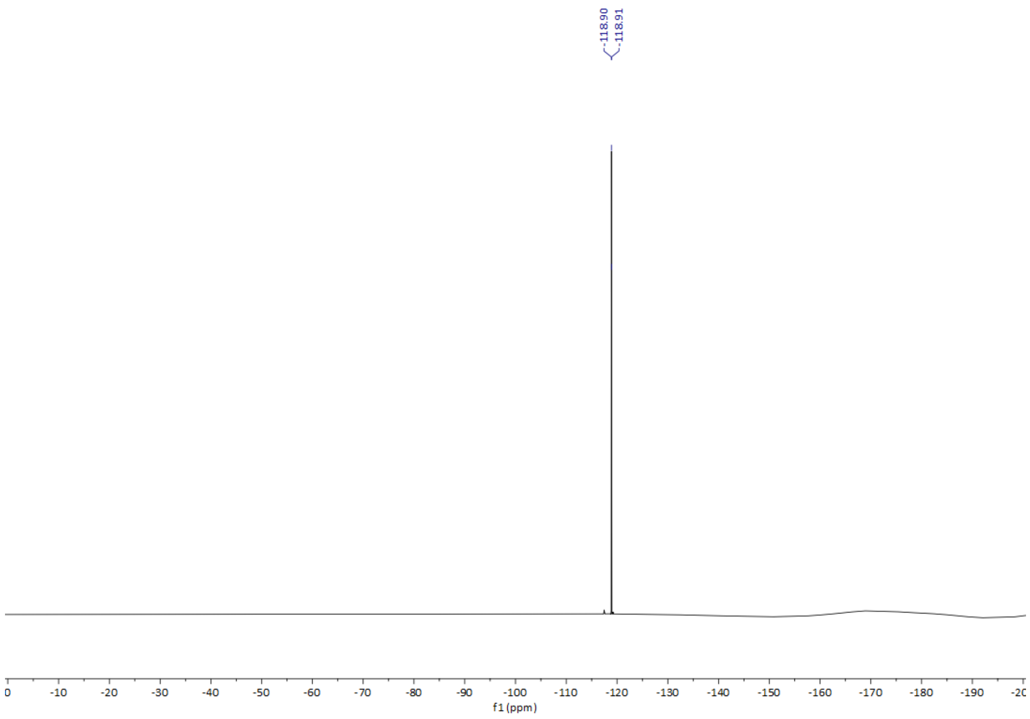

Figure S59: ^{19}F NMR spectrum of **6** in $CDCl_3$.

7. Emission spectra of the LED´s

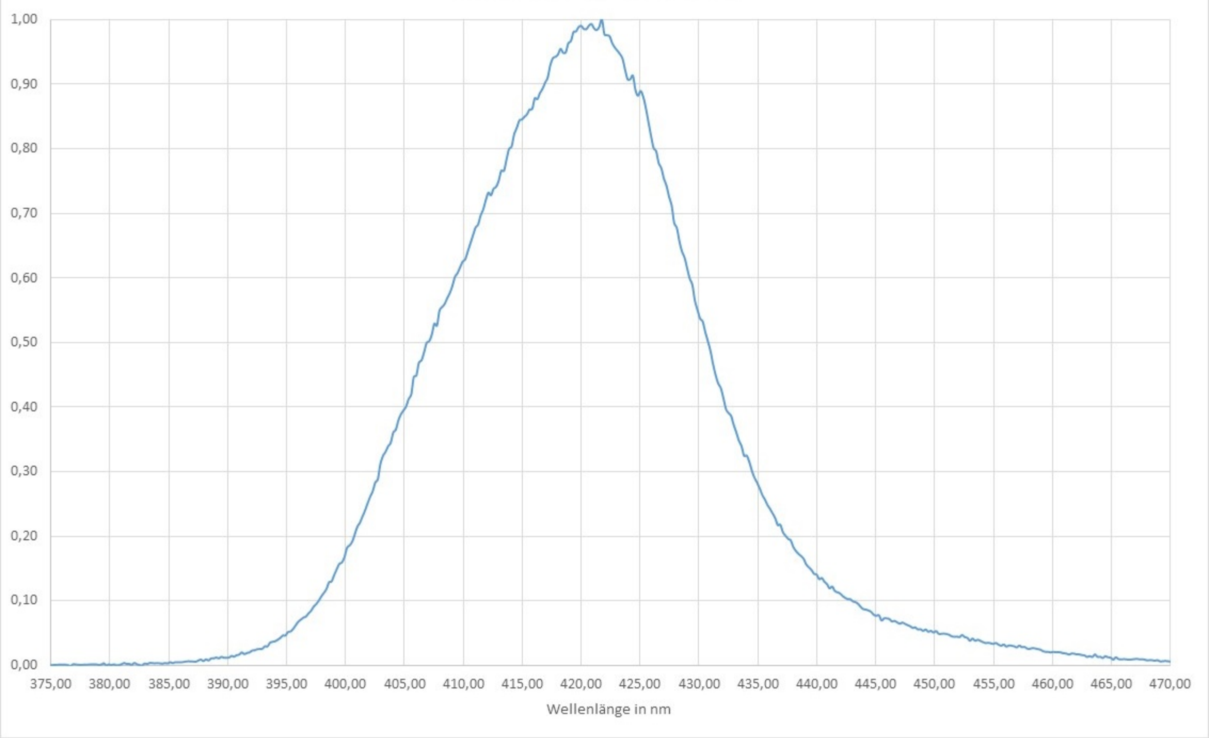

Figure S60: Spectral measurement of the 420 nm (violet) LED measured by Avonec. Reprinted with permission from Avonec.

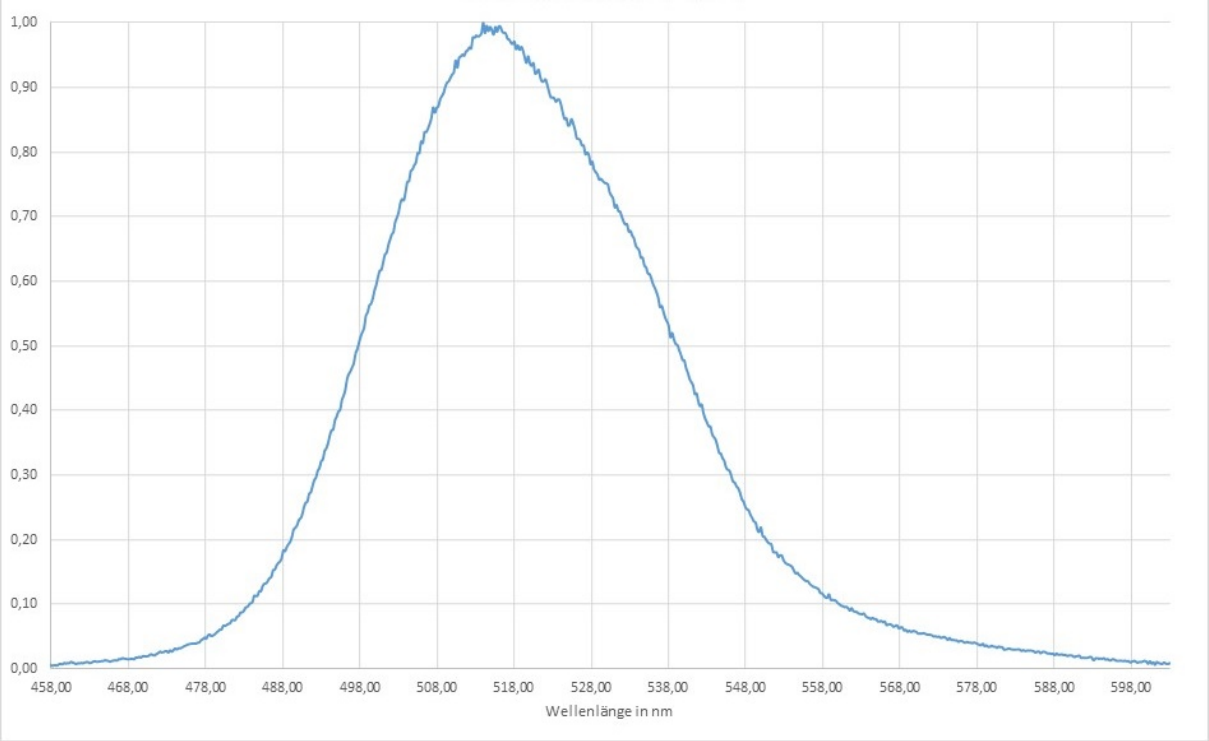

Figure S61: Spectral measurement of the 515 nm (green) LED measured by Avonec. Reprinted with permission from Avonec.

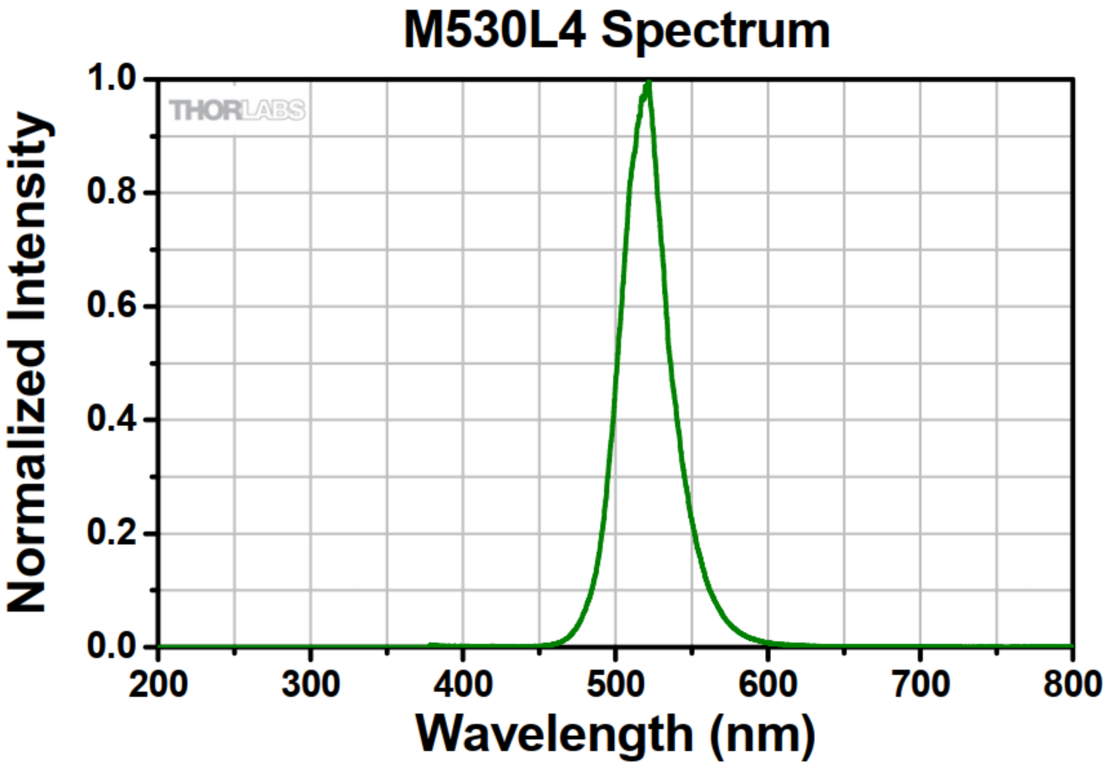

Figure S62: Spectral measurement of the 525 nm (green) LED measured by Thorlabs. Reprinted with permission from Thorlabs.

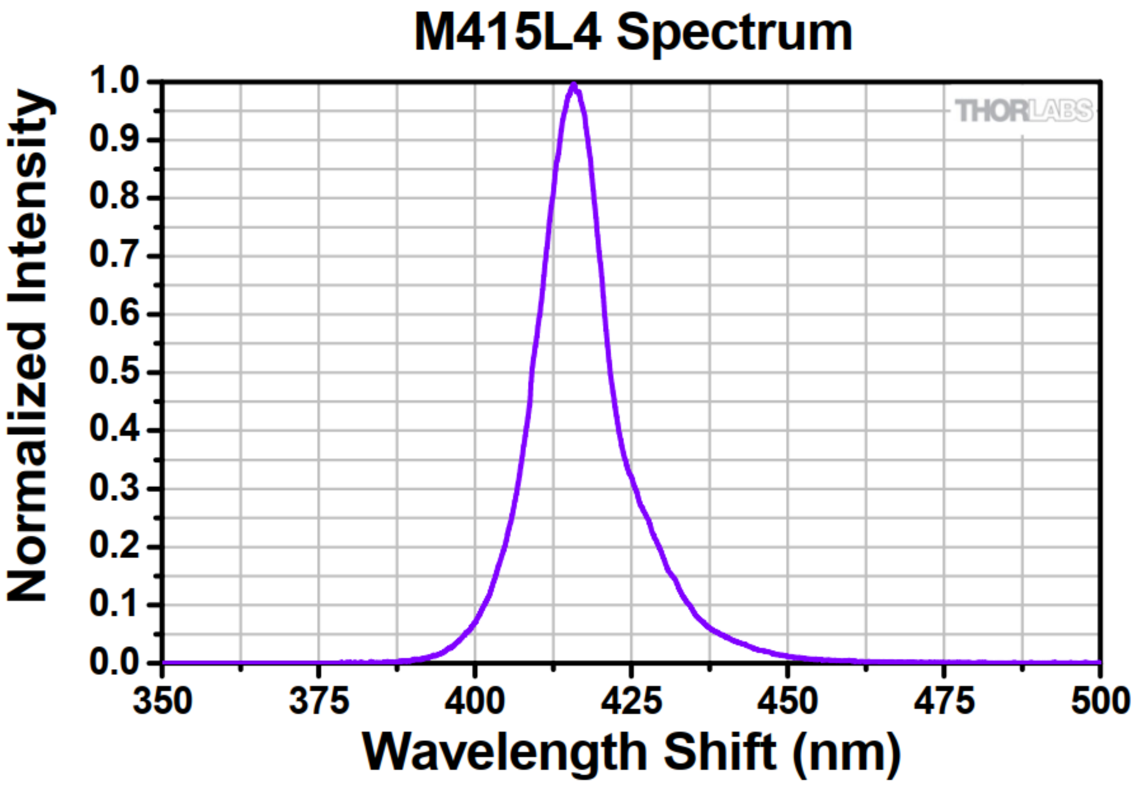

Figure S63: Spectral measurement of the 415 nm (purple) LED measured by Thorlabs. Reprinted with permission from Thorlabs.

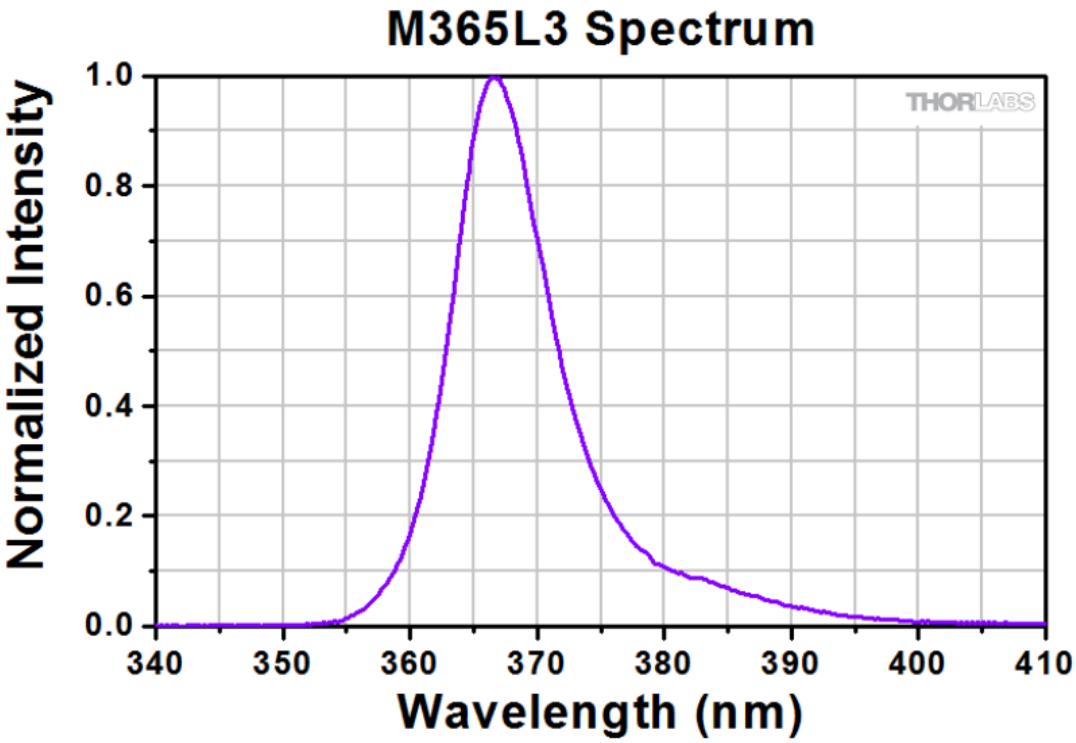

Figure S64: Spectral measurement of the 365 nm (UV) LED measured by Thorlabs. Reprinted with permission from Thorlabs.

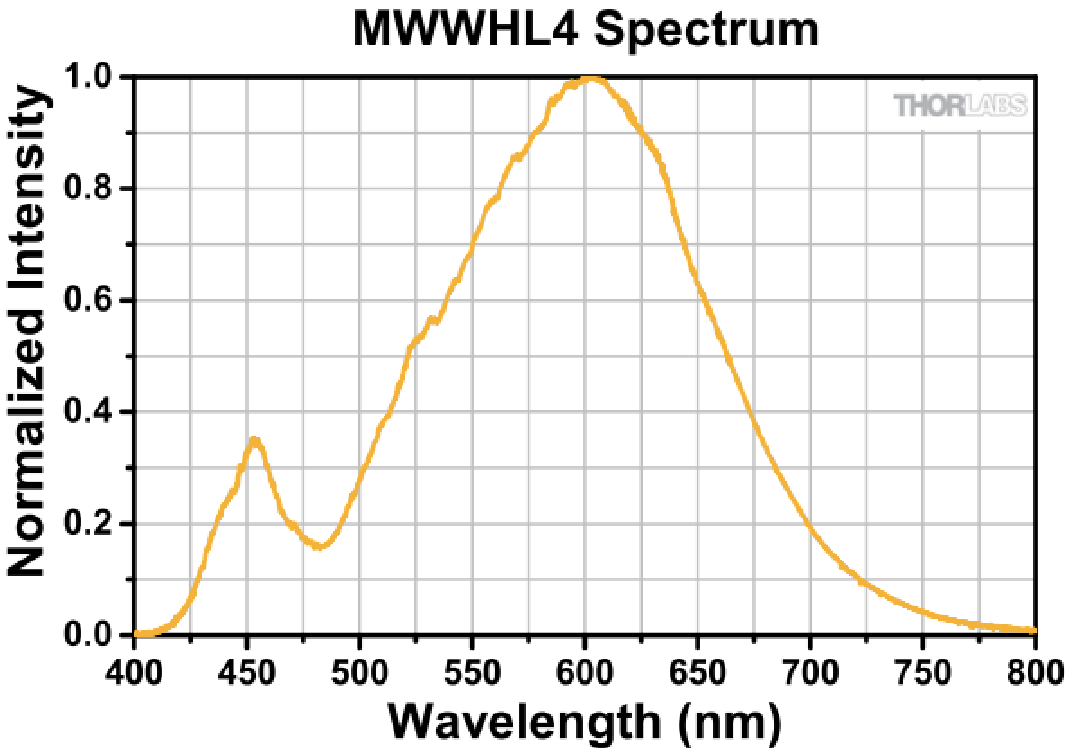

Figure S65: Spectral measurement of the 3000K LED ("warm-white") measured by Thorlabs. Reprinted with permission from Thorlabs.

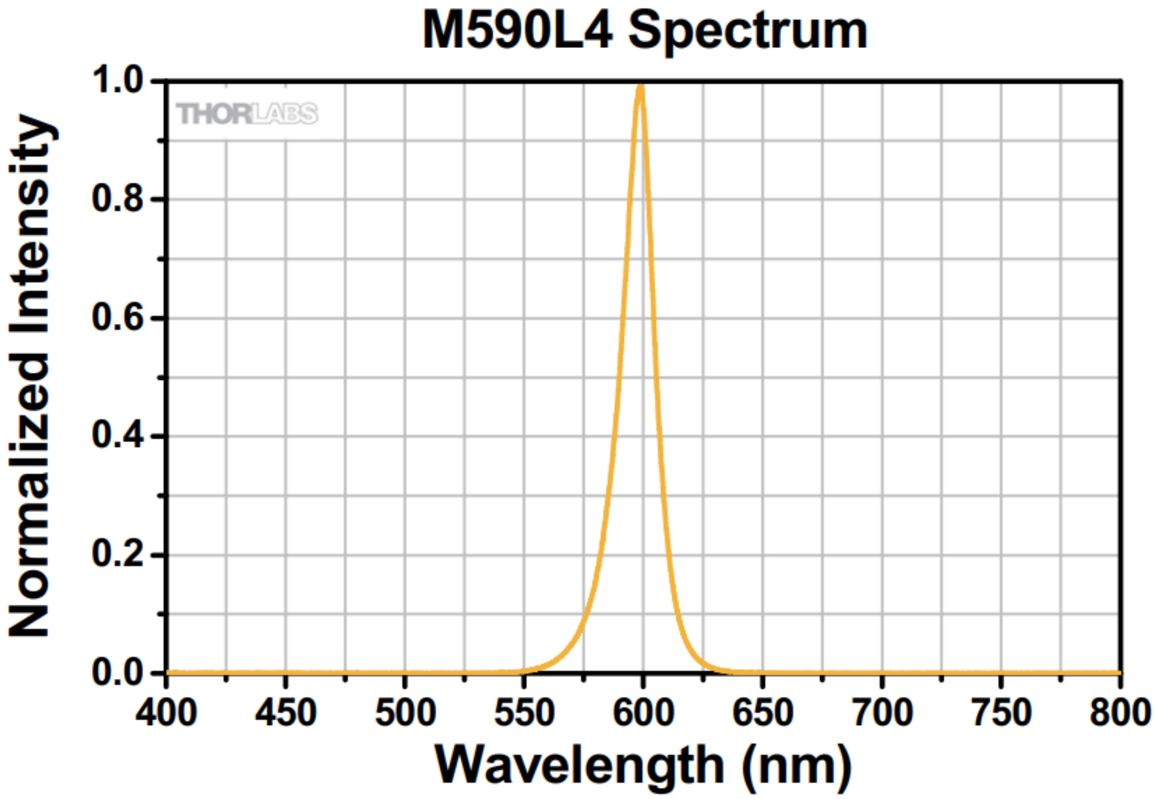

Figure S66: Spectral measurement of the 590 nm (orange) LED measured by Thorlabs. Reprinted with permission from Thorlabs.

8. Crystallographic Analysis

Table S5 : Crystal data and structure refinement for 5.

Formula	$C_{38}H_{38}F_4N_2O_4$
Formula weight, g/mol	662.7
Temperature, K	100
Crystal system	Triclinic
Crystal size, mm^3	0.57 × 0.32 × 0.25
Space group	P1
a, Å	5.4601(5)
b, Å	13.4307(12)
c, Å	22.397(2)
α, °	101.224(4)
β, °	95.268(4)
γ, °	91.462(4)
Volume, Å^3	1602.6(3)
Z	2
ρ_{calc}, g/cm^3	1.373
μ (Mo; Kα), 1/mm	0.104
F(000)	696.0
Θ range, deg	4.348 to 60.728
Index ranges	-7 ≤ h ≤ 7
	-19 ≤ k ≤ 19
	-31 ≤ l ≤ 31
No. of reflns collected	19199
Completeness to Θ_{max}	99.6%
No. indep. reflns	19199 [R_{int} = 0.0533, R_{sigma} = 0.0565]
Data/restraints/parameters	19199/3/870
GooF (F^2)	1.023
R_1 ($I > 2\sigma(I)$ for X-Ray)	R_1 = 0.0921
wR_2 ($I > 2\sigma(I)$ for X-Ray)	wR_2 = 0.2510
R_1 (all data)	R_1 = 0.1282
wR_2 (all data)	wR_2 = 0.2856
Largest diff. peak/hole, e/Å^3	0.68/-0.60
Flack parameter	-0.6(12)
CCDC number	2224454
